# Single‐Atom Photocatalyst as Floatable Artificial Leaf for Upcycling Oceanic Plastic Waste

**DOI:** 10.1002/adma.202519931

**Published:** 2026-03-17

**Authors:** Amin Talebian‐Kiakalaieh, Xin Xu, Wenzhong Ji, Yun Liu, Bingquan Xia, Jingrun Ran, Shi‐Zhang Qiao

**Affiliations:** ^1^ School of Chemical Engineering Adelaide University Adelaide SA Australia; ^2^ Research School of Chemistry ANU College of Science The Australian National University Canberra ACT Australia; ^3^ School of Chemistry and Environmental Engineering, Key Laboratory of Green Chemical Engineering Process of Ministry of Education Wuhan Institute of Technology Wuhan Hubei China

**Keywords:** floatable artificial leaf, in situ characterizations, plastic waste upcycling, single atom photocatalyst

## Abstract

Over ∼8 billion tons of plastic have been produced to date, with ∼80% of them ended up in landfills/oceans. Among them, polypropylene (PP) possesses the lowest global recycling rate (< 1%). To resolve this, Ru single atoms (SAs) loaded photocatalysts (ZnIn_2_S_4_/Ru SAs) in the forms of powder/floatable artificial leaf (AL) were prepared for direct conversion of raw PP plastic into valuable chemicals. The optimized photocatalyst exhibits exceptional performance with a total formic/acetic acid production of 1022.5 µmol g^−1^. In situ X‐ray photoelectron spectroscopy, in situ atomic force microscopy‐kelvin probe force microscopy, and in situ electron paramagnetic resonance (EPR) reveal efficient electron extraction from ZnIn_2_S_4_ nanosheets to Ru SAs, with subsequent electron capture by O_2_ molecules in air. Additionally, in situ transient‐state photoluminescence spectroscopy, transient photovoltage measurement, and in situ EPR unveil the electrolyte‐assisted polarization (induced by cations/anions in seawater) significantly enhancing charge separation/transfer. Finally, in situ EPR, in situ infrared (IR) spectroscopy, and quenching experiments corroborate the pivotal roles of reactive oxygen species (·O_2_^−^/·OH) for upcycling PP into valuable chemicals. These results highlight the transformative potential of floatable AL concept for converting plastic waste into high‐value chemicals, offering a sustainable solution to plastic contamination.

## Graphical abstract

To resolve the catastrophic disaster of oceanic plastic pollution, the novel Ru single atom loaded ZnIn_2_S_4_ photocatalysts, in the forms of powder or floatable artificial leaf, were prepared for direct conversion of raw polypropylene plastic into valuable chemicals. The optimized catalyst exhibits exceptional performance, with a total formic/acetic acid production of 1022.5 µmol g^−1^.

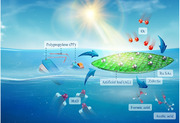

## 1 Introduction

Undoubtedly, our modern society is highly dependent on the production and application of plastics. Nevertheless, current inadequate plastic disposal and end‐of‐life management strategies have caused a catastrophic disaster of global plastic crisis [1, 2, 3]. Annually, ∼11 million tons of plastic waste enter into the oceans, accumulating around surface waters and coastlines (e.g., the great pacific garbage patch). The long‐lasting plastic waste in the environment progressively fragments into micro‐ and nano‐plastics, posing severe threats to marine ecosystems, food security, and human health [4, 5]. Among various plastic types, polyolefins, particularly polypropylene (PP), account for ∼57% of global plastic production and represent a dominant fraction of floating marine debris, owing to their low density and hydrophobic nature. Despite their massive production volume, PP plastics exhibit the lowest global recycling rate (<1%) among common plastic wastes, highlighting a critical gap in current waste‐management strategies [6, 10]. To date, ∼8 billion tons of plastics have been produced worldwide, of which ∼80% have been discarded into landfills or aquatic environments after single or short‐term use, resulting in an estimated annual economic loss of USD 80 to 120 billion [6, 7, 8, 9]. These alarming facts underscore the urgent need to reconceptualize plastic waste not as an environmental liability, but as a carbon‐rich feedstock for the sustainable production of value‐added chemicals. Nevertheless, current mechanical and chemical recycling techniques remain inefficient and economically unfavourable, because they typically rely on high temperature and pressure, energy‐intensive processes, or hazardous chemical reagents, often yielding downgraded or low‐value products [11, 12]. Thus, the development of an efficient, low‐energy, and environmentally benign photocatalytic upcycling strategy is particularly compelling. Such an approach is particularly attractive for floating low‐density plastics (e.g., PP), offering a unique opportunity to directly transform oceanic plastic waste into valuable chemicals/fuels under mild conditions.

**FIGURE 1:**
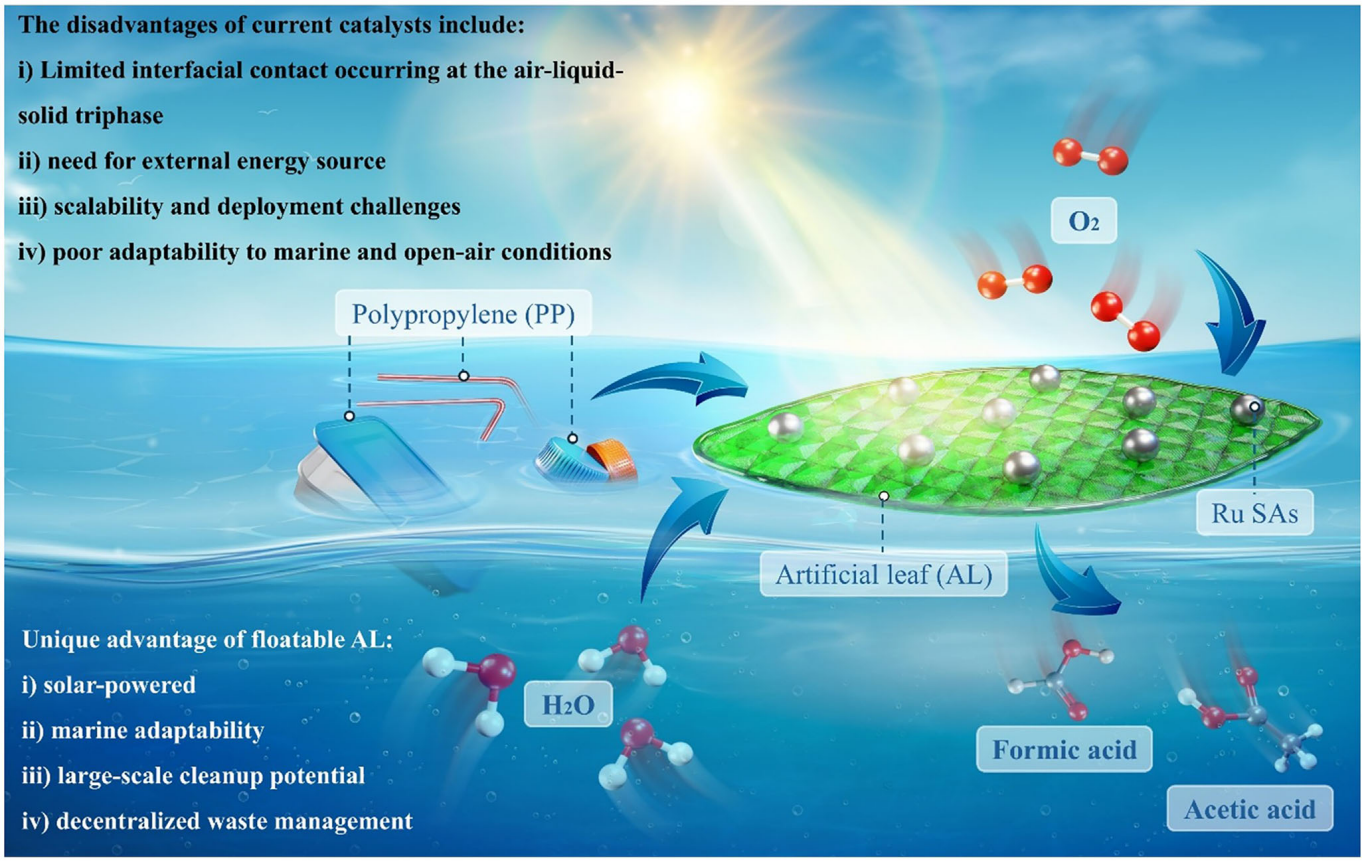
Overview of the floatable AL concept for photocatalytic upcycling of raw PP wastes into formic/acetic acids with the Earth's most abundant resources (sunlight, seawater and air). The disadvantages of current photocatalyst systems and the unique advantages of floatable AL system are highlighted in Figure 1.

Recently, photocatalysis route has emerged as a promising green technology, which can directly convert renewable solar energy into chemicals/fuels through a sustainable and environmentally benign process. Although pioneering studies exhibit the feasibility of photocatalytic upcycling of plastic wastes, the efficiency remains suboptimal. Additionally, these processes often necessitate energy‐intensive pretreatment steps using hazardous solutions (e.g., 10 M KOH), as well as controlled laboratory conditions (e.g., inert gas atmosphere or high‐pressure/temperature‐resistant reactors), limiting their scalability and realistic applicability [6, 13, 14]. In our previous work, we introduced the oceanic refinery (OR) concept as a novel approach to sustainable plastic upcycling [15]. Building upon this, we have now developed the new single‐atom photocatalyst as floatable artificial leaf (AL), designed to maximize interfacial contact among active catalytic sites, raw plastics, air and water at the gas‐liquid‐solid tri‐phase in photocatalytic process. This innovative system can operate without external energy input and only leverage the Earth's most abundant resources (e, g., sunlight, seawater, and air) to offer a scalable and sustainable route for plastic upcycling (Figure 1).

The floatable AL concepts offer transformative solutions for oceanic plastic waste management by addressing the four key limitations in conventional photocatalytic upcycling processes: i) the insufficient contact among the interfaces of catalyst, plastic, water and air for this solid‐liquid‐gas tri‐phase catalytic reaction. This is owing to the relatively low density and hydrophobic nature of most plastic wastes, apparently limiting their dispersion in the aqueous environment. Also, most reported photocatalysts are in powder form uniformly dispersed in the aqueous environment rather than only floating on the surface of water, thus greatly restricting the sufficient interfacial contact of catalyst, plastic and air. Although several floatable photocatalysts have been developed and explored, the costly and relatively instable materials (e.g., aerogels and polymer‐based materials) are usually applied [16, 17, 18, 19, 20]. Compared with these aerogel‐/polymer‐based floating systems, recent studies have revealed that floatable AL prepared from simple and low‐cost supports can provide comparable/higher photocatalytic activity while significantly improving scalability and environmental compatibility. In fact, glass‐/cellulose‐based floatable supports are capable to provide high light exposure, efficient tri‐phase contact, high level of recyclability, lower long‐term degradation, and lower material cost compared to polymeric floatable substrates. These works highlight a growing demand for floatable systems that use inexpensive, chemically inert, and structurally robust supports suitable for large‐scale operation [19, 21, 22]. ii) the insufficient absorption/utilization of the incident light, particularly the ample infrared (IR) light, from the solar spectrum, which accounts for ∼53% in the natural sunlight. This is not only owing to the strong absorption/blocking by the water molecules if the photocatalysts are uniformly dispersed in aqueous environment, but also resulting from the lack of insulation measure for reported photocatalyst systems to maintain the raised system temperature, caused by the absorbed IR light [23, 24]. Actually, the raised temperature of reaction system can not only accelerate the conversion of most plastic waste into monomer/oligomer, but also obviously boost the reaction kinetics; iii) the inadequate exploitation and knowledge of the charge polarization effect aroused by the abundant cations/anions in the seawater; iv) the lack of highly efficient photocatalyst developed and employed in current photocatalytic upcycling systems.

To resolve the above four bottlenecks in conventional photocatalytic plastic upcycling, significant advancements have been realized by the floatable AL technique one by one: i) a porous glass fiber paper was applied as a floatable platform for loading highly efficient photocatalyst developed. This loading strategy also provides clear advantages over other common catalyst immobilization methods (e.g., polymer membranes, binder‐coated films, ceramic substrates, or powder suspensions). Glass‐fiber filters offer a unique combination of high porosity, mechanical robustness, thermal stability, and chemical stability, allowing uniform catalyst deposition without polymeric binders which can block active sites. Their open 3D fibrous form enhances quick water‐air exchange and mass transfer, while their light weight enables natural floatation without the need for additional floatable materials. Recent studies on glass‐fiber‐supported TiO_2_ and related photocatalysts have similarly reported enhanced stability, simple recovery, and robust bonding between catalyst materials and fibers, making this approach particularly suitable for scalable and long‐term photocatalytic applications [25, 26, 27].; ii) this floatable AL enable the effective absorption of incident sunlight, because it is floating on the surface of water rather than in the water phase. Also, we employed an aluminium (Al) foil layer surrounding the reactor as an insulation layer to maintain the raised temperature (65°C) of whole reaction system, apparently increasing the plastic conversion rate; iii) our AL technique also realized the electrolyte‐assisted charge polarization effect by the abundant cations/anions in seawater, exhibiting much higher activities for plastic conversion compared to those in deionized water. This effect was also insightfully explored by the in situ characterization techniques; iv) finally, a novel and highly active photocatalyst, single atom (SAs) Ru loaded ZnIn_2_S_4_ nanosheets (NSs), was for the first time designed, prepared and loaded on the surface of glass fiber paper to acquire the floatable AL, which achieved the highly‐efficient conversion of raw PP plastic into valuable chemicals/fuels.

Overall, we have prepared the Ru SAs loaded ZnIn_2_S_4_ NSs photocatalysts (ZnIn_2_S_4_/Ru SAs) in the forms of powder/floatable AL with high efficiency for the direct conversion of raw PP plastic into valuable chemicals, by efficiently utilizing the whole spectrum of sunlight, seawater and air. The optimized ZnIn_2_S_4_/Ru SAs catalyst (R1.00) exhibit exceptional performances, achieving high yields of liquid (∼1022.5 µmol g^−1^) and gas (∼129.1 µmol g^−1^) products, ranking R1.00 among the most active catalysts for photocatalytic reforming PP [6, 13, 28, 29, 30]. The control experiments confirm the critical role of Ru SAs, seawater, air, or IR light. A range of advanced in situ/ex situ characterizations were employed to reveal the atomic‐level structure‐performance relationship and the reaction mechanism in realistic conditions. In situ X‐ray photoelectron spectroscopy, in situ atomic force microscopy‐kelvin probe force microscopy, and in situ electron paramagnetic resonance (EPR) reveal efficient photo‐generated electron pumping by Ru SAs from ZnIn_2_S_2_ NSs. And these electrons at Ru SAs can effectively reduce O_2_ molecules in air. Additionally, transient‐state photoluminescence spectroscopy, transient‐state photovoltage measurements, and EPR reveal that electrolyte‐assisted polarization (induced by cations/anions in seawater) apparently accelerate electron‐hole separation/transfer. Finally, in situ EPR, in situ IR spectroscopy, and quenching experiments confirm the pivotal roles of reactive oxygen species (·O_2_^−^/·OH) in oxidizing PP into valuable chemicals. The above results reveal the potential impact of floatable AL photocatalytic platform for upcycling plastic waste into valuable chemicals, a scalable and sustainable solution to resolve global plastic issue.

## 2 Results and Discussion

### 2.1 Morphology, Composition and Structure of Photocatalyst

Initially, ZnIn_2_S_4_ nanosheets (NSs) were prepared by a facile hydrothermal process. Then, a range of ruthenium (Ru) atoms were anchored on the surface of ZnIn_2_S_4_ NSs by an impregnation route. The as‐prepared catalysts are labelled as R0.25, R0.50, R1.00, and R2.00, respectively, based on the nominal weight ratios (0.25, 0.5, 1, and 2 wt%) of Ru element in the as‐prepared catalysts. Pure ZnIn_2_S_4_ NSs are labelled as R0.00. The preparation routes are recorded in supported information. The X‐ray diffraction (XRD) pattern of pure ZnIn_2_S_4_ (R0.00), along with the corresponding joint committee on powder diffraction standards (JCPDS) card (#72‐0773), are presented in Figure S1. These results reveal the hexagonal phase of ZnIn_2_S_4_ for R0.00. The TEM image of R0.00 (Figure S2a) confirm the ultrathin thickness and large lateral sizes (∼100–300 nm) of ZnIn_2_S_4_ NSs. The high angle annular dark field (HAADF)‐scanning transmission electronic microscopy (STEM) image of R0.00 are exhibited in Figure S2b. The rectangle area in Figure S2b were further explored and the corresponding atomic‐resolution differential phase contrast (DPC)‐STEM image (Figure S2c) of R0.00 further validate the hexagonal phase of ZnIn_2_S_4_ and confirm the presence of Zn and In atoms, though S atoms remain indistinguishable owing to their lower atomic contrast [31]. For the red‐lined region of R0.00 (Figure S2c), the corresponding modelled atomic structure of ZnIn_2_S_4_ (Figure S2d) and line analysis along the red dash line 1 in Figure S2c are exhibited in Figure S2e. Moreover, the high‐Resolution HAADF‐STEM image of R0.00 (Figure S2f) exhibits the lattice spacing values of 2.9 and 3.3 Å with an angle of 117.6°, corresponding to the (013) and (−100) facets of hexagonal ZnIn_2_S_4_, respectively. The EDX spectrum (Figure S2g), HAADF‐STEM image (Figure S2h), and corresponding elemental mapping images (Figure S2h) of R0.00 together confirm the successful preparation of ZnIn_2_S_4_ NSs, in accordance with the above TEM and HAADF‐STEM results [32].

**FIGURE 2:**
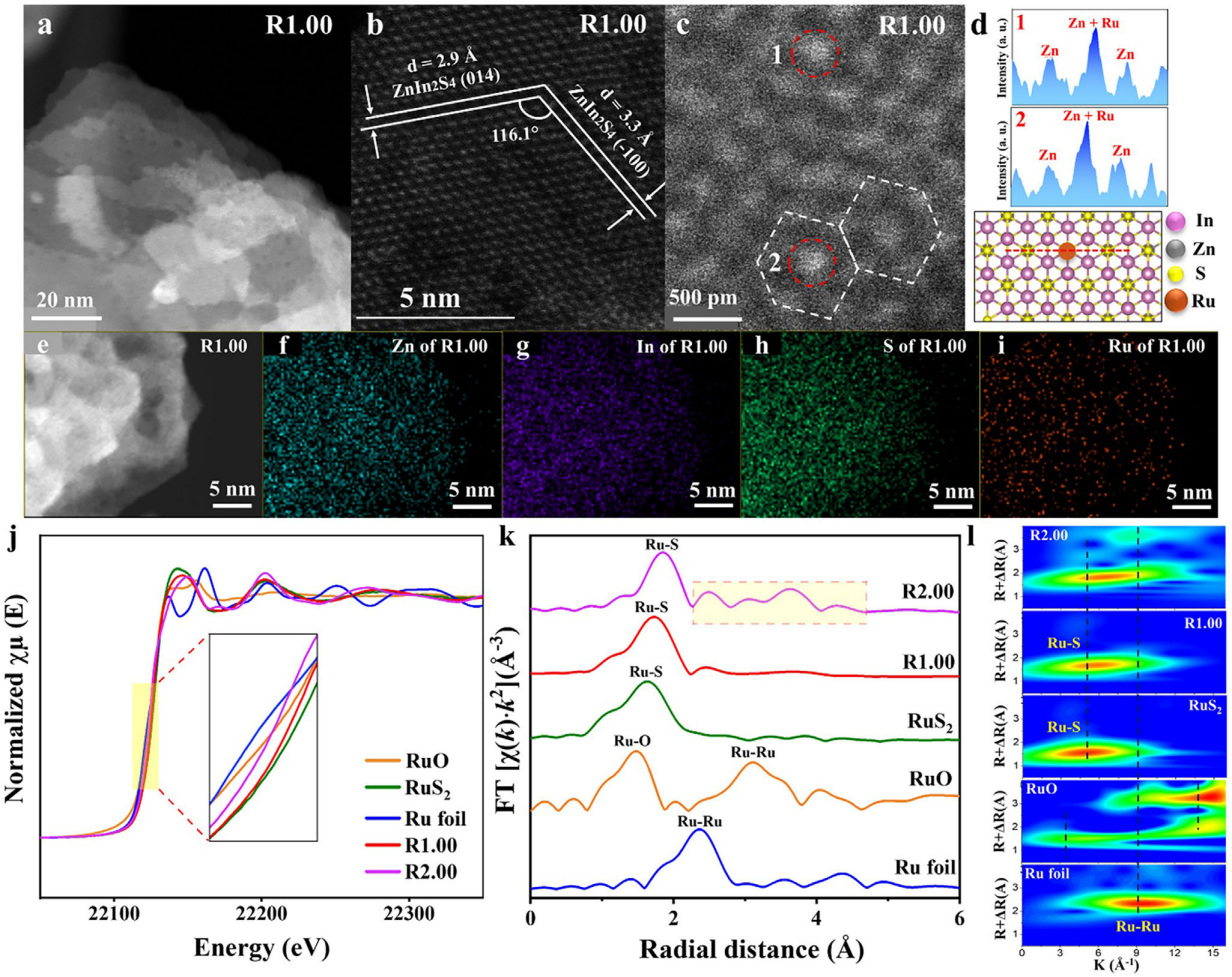
(a) HAADF‐STEM image and (b) Atomic‐Resolution HAADF‐STEM image of R1.00. (c) Atomic‐Resolution DPC‐STEM image and (d) the corresponding line analyses across region 1 and region 2 for Ru SAs on ZnIn_2_S_4_ and modelled atomic structure of R1.00. (e) HAADF‐STEM image and the corresponding elemental mapping images of (f) Zn, (g) In, (h) S, and (i) Ru elements for R1.00. (j) Synchrotron‐based Ru K‐edge XAS spectra for R2.00, R1.00, Ru foil, RuS_2_, and RuO. (k) Fourier‐transformed extended X‐ray absorption fine structure (EXAFS) spectra for Ru foil, RuO, RuS_2_, R1.00, and R2.00. (l) Ru K‐edge WT‐EXAFS spectra for R2.00, R1.00, RuS_2_, RuO, and Ru foil.

Then, we explored the morphology, composition and structure of R1.00 via advanced characterizations. The HAADF‐STEM image of R1.00 (Figure 2a) further confirm its layered sheet structure, in accordance with the TEM (Figure S2a) and HAADF‐STEM (Figure S2h) images of R0.00. These results reveal that the loading of Ru SAs doesn't arouse obvious change on the morphology of ZnIn_2_S_4_ NSs. The HAADF‐STEM image of R1.00 (Figure 2b) reveal the lattice spacings of 2.9 and 3.3 Å, with an angle of 116.3°, corresponding to the (014) and (−100) facets of ZnIn_2_S_4_ (Figure 2b). The DPC‐STEM image of R1.00 (Figure 2c) confirms the presence of isolated Ru atoms, appearing as bright spots on the surface of ZnIn_2_S_4_ matrix. This is further verified by intensity profile line analyses (Figure 2d) across region 1 and 2 in Figure 2c, which exhibits single peak characteristic of individual Ru atoms, with no evidence of Ru clusters/nanoparticles on the surface of ZnIn_2_S_4_. Furthermore, a modelled atomic structure of R1.00 is provided in Figure 2d. And the HAADF‐STEM image (Figure 2e) and corresponding elemental mapping images (Figure 2f–i) confirm the homogeneous dispersion of Ru atoms on the surface of ZnIn_2_S_4_. Additional HAADF‐STEM images (Figure S3a–e), along with the EDX spectrum (Figure S3f) of R1.00, are provided to further confirm the presence of isolated Ru atoms (bright spots) on the surface of ZnIn_2_S_4_ NSs in R1.00. Moreover, Figure S3g exhibits the electron paramagnetic resonance (EPR) spectrum of R1.00 with g = 2.005, confirming the presence of delocalized electrons (unpaired electrons) in the as‐prepared R1.00 [33]. This result reveals the presence of S vacancies (Vs) in R1.00. Furthermore, the successful anchoring of Ru atoms on ZnIn_2_S_4_ NSs is further supported by inductively coupled plasma‐atomic emission spectroscopy (ICP‐AES) result, revealing the actual content of Ru (0.816 wt%) in R1.00. This value is slightly lower than the nominal weight ratio (1 wt%) of Ru in R1.00.

The crystal/phase structures of all the as‐prepared catalysts were explored by XRD. The XRD patterns of all the as‐prepared catalysts (Figure S4) indicate the hexagonal phase structure of ZnIn_2_S_4_ (JCPDS#72‐0773). No other peaks are found in the XRD patterns of R1.00, implying the absence of Ru‐based clusters/nanoparticles in R1.00 [34]. Interestingly, R2.00 exhibits similar XRD patterns, compared with those of R1.00. This is probably because the Ru species amount is relatively low (< 2 wt%), which is below the detection sensitivity of the XRD technique [35]. The HAADF‐STEM images of R2.00 (Figure S5a–c) clearly exhibit the presence of bright cloudy regions on the surface of ZnIn_2_S_4_. Also, the corresponding EDX spectrum (Figure S5d) confirms the presence of Ru element, revealing the agglomeration of Ru atoms into Ru nanoparticles on the surface of ZnIn_2_S_4_ NSs in R2.00. These are further confirmed by the HAADF‐STEM image (Figure S5e) and corresponding elemental mapping images (Figure S5f–i), in which a bright region is highlighted by red circles (Figure S5e,f), implying the presence of Ru nanoparticles on the surface of ZnIn_2_S_4_ NSs. Th above results reveal the overloading of Ru in R2.00.

Furthermore, the synchrotron‐based X‐ray absorption spectroscopy (XAS) was employed to investigate the coordination environment and electronic structure of Ru species on R1.00. Figure 2j exhibits the XAS spectra of Ru K‐edge region for R1.00 and the reference samples (R2.00, Ru foil, RuO, and RuS_2_). The inset in Figure 2j indicates that the center peak energy is between that of RuO and RuS_2_, revealing that the oxidation state of Ru is between +2 and +4. The absorption threshold at 22 119 eV for Ru foil corresponds to the heteroatomic interactions between the metal center of the Ru atom on 5p orbital and the neighboring atoms on the 4d and 5p orbitals [36, 37]. The observed values of the absorption threshold for R1.00 (22 126 eV) and RuS_2_ (22 128 eV) were larger than that for Ru foil, indicating the increased transition energy from 1s to the outermost shell orbitals of Ru atoms. The main reason for the increasing of transition energy is the formation of Ru‐S bonds in R1.00 [38]. To further analyze the oxidation state of Ru in R1.00, the absorption edge energy (E_0_) is applied as a function of Ru oxidation state in the prepared sample. Thus, the average Ru oxidation state of R1.00 is determined to be +3 (Figure S6a,b) [39, 40]. The Fourier transform k^2^‐weighted extended X‐ray absorption fine structure (FT‐EXAFS) spectra are exhibited in Figure 2k. R1.00 exhibits a dominant peak at 1.7 Å in the R‐space (Figure 2k), attributed to the Ru‐S coordination. This observation confirms the atomically dispersed nature of Ru on the surface of R1.00. The strong similarity between the EXAFS spectra of R1.00 and the RuS_2_ reference (Figure 2k) further reveals that Ru is highly stabilized through Ru‐S bonding interactions with ZnIn_2_S_4_ [41]. Additionally, wavelet transform (WT) analysis of EXAFS spectra (Figure 2l) exhibits a single intensity maximum at ∼5.5 Å^−^^1^ in k‐space, assigned to Ru‐S bonding. The absence of Ru‐Ru coordination, as observed in Ru foil or RuO references, unequivocally confirms the atomically dispersed nature of Ru in R1.00. Furthermore, The XAS analysis was applied to clearly compare the Ru coordination environments of R1.00 and R2.00 with each other. In fact, the FT‐EXAFS spectrum (Figure 2k) of R2.00 exhibits multiple well‐resolved peaks between ∼2.2–4.5 Å (Figure 2k), clearly indicating that the Ru atoms are not isolated SAs. Indeed, there are two possible reasons for later phenomenon: (i) The majority of Ru atoms exist in the form of cluster/nanoparticles (NPs). Actually, the Ru‐Ru interactions extend beyond the first coordination shell, resulting in apparent second‐ and third‐shell scattering features in the EXAFS spectrum (Figure 2k). (ii) Typically, for Ru cluster/NP, multiple‐scattering pathways (e.g., Ru‐Ru‐Ru) become significant and generate additional EXAFS contributions at longer radial distances [42, 43]. Also, the WT‐EXAFS analysis (Figure 2l, top) of R2.00 reveals the apparent intensity contributions attributed to Ru‐S, Ru‐Ru, and Ru‐O coordination, further confirming the coexistence of multiple Ru chemical environments. In comparison, R1.00 exhibits only a single dominant Ru‐S peak in both FT‐EXAFS (Figure 2k) and WT‐EXAFS (Figure 2l) spectra, characteristics of atomically dispersed Ru single atoms. Altogether, the above XAS results reveal that Ru exists predominantly as isolated single atoms within R1.00. In comparison, aggregated Ru species exist in R2.00 in the form of clusters/NPs. High‐resolution X‐ray photoelectron spectroscopy (XPS) results were also collected to elucidate the chemical state of Ru single atoms (SAs) and their interactions with ZnIn_2_S_4_. As exhibited in Figure S7a,b, the Zn 2p and S 2p peaks of R1.00 exhibit the movement to lower binding energy direction upon Ru SA loading, compared to those of R0.00. In contrast, the In 3d peak of R1.00 (Figure S7c) remain unmoved, compared to that of R0.00. These results indicate that charge transfer between Ru SAs and ZnIn_2_S_4_ primarily occurs via Zn‐S‐Ru bonds, highlighting a strong electronic interaction between Ru SAs and ZnS layers rather than the InS_2_ layers in R1.00 [44, 45].

### 2.2 Photocatalytic Performances for Raw Plastic Conversion

**FIGURE 3:**
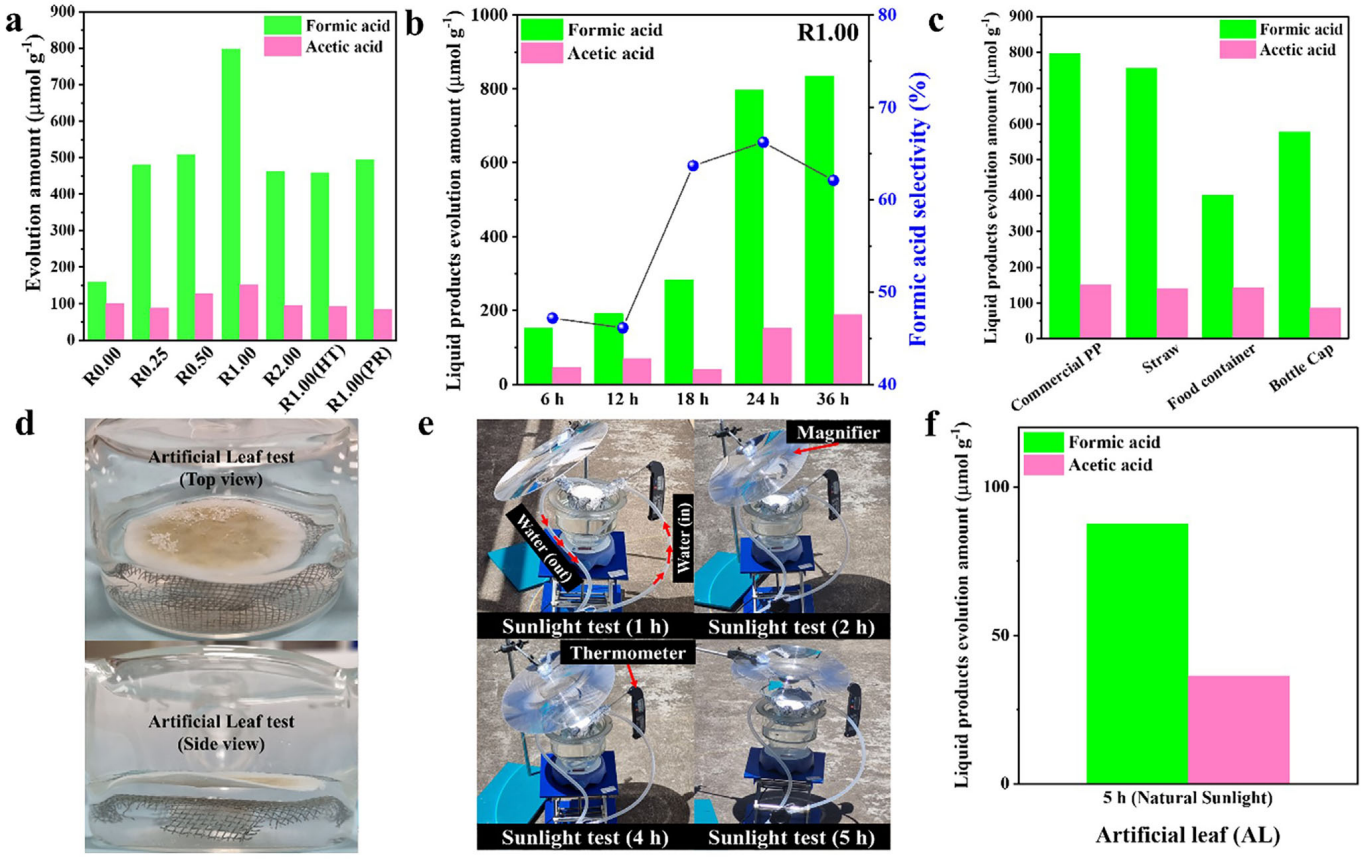
(a) Photocatalytic reforming raw PP utilizing R0.00, R0.25, R0.50, R1.00, R2.00, R1.00(HT), and R1.00(PR) in seawater with xenon light irradiation in air for 24 h. (b) Photocatalytic reforming raw PP utilizing R1.00 in seawater with xenon light irradiation in air for 36 h. (c) Photocatalytic reforming real PP plastic waste in the forms of straw, food container, and bottle cap after grinding by utilizing R1.00 in seawater with xenon light irradiation in air for 24 h. (d) The AL test from top and side views. (e) The test of AL photocatalyst under natural sunlight irradiation for 5 h. (f) The photocatalytic activities of AL under natural sunlight irradiation for 5 h.

Owing to its outstanding physicochemical properties, for example, excellent thermal resistance and strong chemical stability, PP is widely utilized in various daily/industrial applications. Nevertheless, its strong resistance to chemical‐ and bio‐degradation results in its gradual accumulation in nature and suffers from less than 1% global recycling rate. Hence, PP is selected as the substrate for this work. Remarkably, our research reports a facile one‐step photocatalytic reaction for the direct conversion of PP plastic waste into high value chemicals in realistic conditions. This is unique compared to all the previous reports in this area [6, 8, 13, 28, 29, 30]. The reaction is performed in the presence of seawater and a photocatalyst using xenon light irradiation to create the realistic oceanic condition with sunlight, seawater and air. Following our previously reported oceanic Refinery (OR) concept, a reflective insulation layer is applied to cover the reactor, leaving only the top exposed to maximize light absorption. This design not only utilizes light as the sole energy source but also effectively traps infrared (IR) radiation, enhancing photocatalytic efficiency and raising the reaction temperature to ∼67°C. As illustrated in Figure 3a, the presence of Ru SAs plays a crucial role in enhancing photocatalytic activity. Bulk ZnIn_2_S_4_ exhibits the lowest yields of formic acid (∼158.1 µmol g^−1^) and acetic acid (∼99.3 µmol g^−1^), whereas Ru SAs loaded catalysts (R0.25, R0.50, R1.00 and R2.00) present significantly raised performances. Notably, R1.00 achieves the highest activity, with a total liquid product yield of ∼948.2 µmol g^−1^ after 24 h (Figure 3a). This represents a 3.68‐fold increase compared to R0.00 (Figure 3a and Figures S8 and S9). As exhibited in Figure S9c, only formic acid and acetic acid exist as the major products for generated liquid products from R1.00 after 24‐h reaction. The other peaks are probably attributed to seawater impurities or other unknown products. These results reveal that by increasing the Ru SAs loading from 0.25 to 1.00 wt% on ZnIn_2_S_4_, the photocatalytic activities are also gradually raised. However, higher Ru loading (R2.00) results in a dramatic reduction in formic acid and acetic acid production amounts (Figure 3a). This reduction is attributed to Ru atom agglomeration on the ZnIn_2_S_4_ surface (as evidenced by Figures S5b,c,e,f), resulting in less efficient charge extraction and a reduced amount of accessible active sites. Additionally, two alternative R1.00 catalysts were prepared utilizing hydrothermal (HT) and photoreduction (PR) routes, respectively, to assess the impact of different Ru loading techniques. The resulting catalysts are labelled as R1.00(HT) and R1.00(PR), respectively. Interestingly, the above two catalysts exhibit the obviously lower photocatalytic activities (Figure 3a), compared to R1.00, prepared by the wet impregnation/stirring route. These results highlight the effectiveness of this route in achieving the optimal Ru dispersion and the highest catalytic performance, accordingly.

Moreover, to explore the influence of key reaction parameters on photocatalytic performance and to optimize reaction conditions, R0.00, R1.00, and all control catalysts underwent a 12‐h photocatalytic reaction. The control experiments results confirm the critical role of several reaction parameters, including Ru SAs, photocatalyst presence, light irradiation, air, elevated temperature, and seawater. As exhibited in Figure S10, negligible amounts of liquid products were detected in the absence of light, catalyst, air, or PP plastic. Furthermore, conducting the reaction at room temperature (without the insulation layer) drastically reduced the activity of R1.00 to a total yield of only ∼68.7 µmol g^−1^ for formic and acetic acid evolution. The impact of insulation layer in raising the reaction temperature was investigated by providing the reaction temperature curves for the reaction systems with and without insulation layer, respectively. As exhibited in Figure S11, the insulation layer can significantly increase the reaction temperature to 67°C, compared to only 38°C without insulation layer. The most striking observation was the key role of seawater: in its absence, only ∼127.6 µmol g^−1^ of liquid products were generated after 12 h. This dramatic enhancement is attributed to the abundant cations and anions in seawater, which facilitate the extraction of photoexcited charge carriers from the bulk to the catalyst surface. This effect will further be explored in the following sections [46]. Furthermore, the presence of sulphur vacancies (V_S_) plays a key role in enhancing the photocatalytic activity/efficiency. The EPR result of R1.00 (Figure S3g) confirm the formation of V_s_, which introduce localized defect states, capable of improving charge separation and suppressing electron‐hole recombination [47, 48]. These V_S_ also provide electronically unsaturated anchoring sites for Ru SAs. The EXAFS analysis result (Figure 2k) exhibits clear Ru‐S coordination, indicating the strong interaction between Ru atoms and sulphur sites adjacent to V_S_. This metal‐support interaction optimizes the electronic structure of Ru, facilitating hydrogen abstraction and radical formation on the polymer chains [49, 50]. In addition, V_S_ promotes the generation of reactive species and the visible‐light absorption [51, 52, 53]. Thus, these synergistic effects significantly enhance the catalytic activity of R1.00 for plastic bond activation and conversion.

The long‐term stability and robustness of R1.00 photocatalyst for the conversion of raw PP into high‐value chemicals were evaluated over a 36‐h reaction. As exhibited in Figure 3b, R1.00 exhibits the exceptional activity, achieving a total liquid products yield of ∼1022.5 µmol g^−1^ (formic acid and acetic acid) together with ∼129.3 µmol g^−1^ of gaseous products (Figure S12a), including hydrogen (H_2_), methane (CH_4_), ethane (C_2_H_6_), ethylene (C_2_H_4_), and carbon monoxide (CO). Notably, formic acid remains the major product throughout the reaction, reaching a maximum selectivity of 66.2% after 24 h reaction. The small increase of the products evolution amount (formic acid and acetic acid) from 24 h to 36 h light irradiation is likely attributed to various limiting factors: (i) The consumption/depletion of dissolved O_2_ in the reaction media (seawater) significantly reduces the availability of photoexcited electron acceptor, which is essential for the oxidation reaction. (ii) After converting most of the easily oxidizable fractions of PP in the initial 24 h, the remaining fractions of PP are more resistant to be oxidized and converted. This indicates the slower/harder degradation/upcycling. Actually, only 20 mg PP was applied in each test. (iii) The reaction environment becomes cloudy/less transparent owing to fragmented polymer particles, which reduce/block the light absorption required to drive the reaction. (iv) The accumulation of partially oxidized intermediates can block the reactive sites on the catalyst surface. (v) After prolonged light irradiation in seawater, the possible catalyst photo‐corrosion may also lead to a small increase of products generation after 24 h. Furthermore, the recyclability of R1.00 was investigated by three consecutive 12‐h tests and the results are exhibited in Figure S13. In detail, R1.00 was separated/filtered after each recycling test, washed, dried, and used for another 12 h test with new reactant to examine the recyclability of catalyst. R1.00 exhibits good stability in the first two 12‐h recycling tests. But its activity was reduced by ∼1.6 folds in the third recycling test. The superior performance of R1.00, ranking it among the most efficient photocatalysts for direct PP and other polyolefins such as polyethylene (PE) conversion reported to date (Figure S14 and Table S1) [13, 28, 30, 54, 55, 56, 57, 58]. In fact, the obtained results in our research work is over 43 times higher than that achieved by the Nb_2_O_5_ photocatalyst generating acetic acid [28], ∼6.2 times higher than that generated by the CN_x_‐Ni_2_P in the presence of 10 M KOH yielding H_2_ [13], and ∼1.6 times higher than that yielded by the VPoM/CNNS‐15 in the presence of acetonitrile producing formic acid (Figure S14) [30]. In addition, R1.00 exhibits higher activity compared to the floatable hybrid TiO_2_ photocatalyst in conversion of PE (< 38 µmol g^−1^ h^−1^) and slightly lower to its activity (< 55 µmol g^−1^ h^−1^) in PP conversion. It should be noted that over five times catalyst and plastic amount were used in case of the floatable hybrid TiO_2_ [58]. This remarkable performance can be attributed to the optimized charge carrier dynamics, enhanced surface reaction kinetics, and superior light‐harvesting abilities [6, 28, 29, 30]. In contrast, the long‐term stability of R0.00 without Ru SAs is apparently inferior, exhibiting a nearly fourfold lower activity after 24 h reaction, compared to R1.00 (Figure S15a). In addition, the recycling tests of R0.00, clearly exhibits significant reduction of formic acid and acetic acid production throughout three consecutive recycling tests from ∼198.5 to 48.53 µmol g^−1^ (Figure S15b), indicating the ∼4‐time reduction on the photocatalytic activity.

Then, real‐world PP plastics (e.g., straw, food container, and bottle cap) conversion to value‐added chemicals were investigated. Figure 3c and Figure S12b exhibit the exceptional activity of R1.00 in upcycling various real‐world PP plastic wastes in the 24 h reaction. As presented in Figure 3c, the photocatalytic conversion of PP straw waste exhibits liquid products evolution amount of ∼896.02 µmol g^−1^ with ∼64.8% selectivity for formic acid. These results are near to the commercial raw PP conversion results with liquid products amount of 948.2 µmol g^−1^ and formic acid selectivity of 66.2% (Figure 3c). However, in comparison, food containers and bottle caps exhibit the apparently lower performances for their photocatalytic conversion to both liquid and gas products (Figure 3c and Figure S12b). Particularly, the lowest liquid product amount of 544.9 µmol g^−1^ with only 51.1% selectivity for formic acid is achieved by the food container conversion utilizing R1.00. The main reasons for the lower performances of real‐world PP plastic (e.g., food container and bottle cap) compared to the commercial raw PP is primarily attributed to two factors: (i) the presence of additives (e.g., stabilizers, pigments, fillers, and plasticizers) and (ii) the crystallinity, molecular structure and morphological alterations of plastic during the manufacturing and chemical modification processes [59, 60]. These results (Figure 3c) highlight the critical influence of manufacturing processes on their end‐of‐life behaviors. Moreover, to evaluate the robustness of R1.00 after a 24 h reaction, the corresponding TEM, EDX, and XRD analyses (Figure S16) were conducted. These results reveal no significant alterations in morphology, size distribution and crystal/phase structure of R1.00 after the reaction, revealing the high robustness of R1.00.

Finally, application of the floatable AL concept was employed for the oceanic plastic waste conversion into value‐added chemicals. The floatable AL referred to a light and hydrophilic catalytic bed, which can float on the water and allow water to go through or have contact with both the photocatalyst and reactants (plastic and air). The design of this floatable AL is to maximize the tri‐phase interfacial contact among the photocatalyst, plastic waste, water and air under light irradiation to raise the photocatalytic efficiency. The comprehensive images presenting the selection, testing, and preparation routes of floatable AL have been reported in the supporting information (Figure S17a–g). We have utilized a spray‐vacuum filtration route (Figures S17d and S18) for preparing the floatable AL samples with different loading weights (5–50 mg) of photocatalysts to acquire the smooth surface without agglomeration of samples and the homogeneous distribution of photocatalyst on the surface of floatable AL. Also, the floatability of the prepared AL photocatalyst was tested and exhibited in Figure 3d and Figure S17e–g. Notably, the floatability of AL was significantly affected by the weight of loaded catalyst. ALs with more than >20 mg of loaded catalyst were unable to float on the seawater surface and sank. Also, similar amount of PP (20 mg) was used in the real sunlight test. As introduced in section S1.3 (Supplementary Information), a light concentrator was utilized to reveal the effectiveness of simulated solar‐concentrated photocatalytic conditions. Figure S19a,b clearly illustrate a significant increase of temperature after employing the light concentrator, reaching 243 °F = ∼117°C (outdoor) or 150 °F = ∼65°C (inside the reactor cooled by circulating water). Then, the as‐prepared floatable AL samples were tested under natural sunlight irradiation for 5 h with a light magnifier to concentrate the light on the surface of floatable AL and simultaneously raise the reaction temperature (Figure 3e). Finally, the quantitative analysis through High‐Performance Liquid Chromatography (HPLC) technique clearly confirms the production of formic and acetic acids in the first 5 h of the test under natural sunlight irradiation (Figure 3f). All the acquired results strongly confirm our main hypothesis that floatable AL photocatalysts is applicable in realistic photocatalytic oceanic plastic waste upcycling process by providing a more sustainable, scalable, and environmentally friendly route, compared to the conventional processes. In comparison to the conventional photocatalysts, the main benefits of our floatable AL photocatalyst can be summarized as follows: i) no need for external energy source other than natural solar energy, ii) adaptability for marine environments owing to its easy floatability on the ocean surface, iii) adaptability for contaminated coastlines or remote/underdeveloped regions, iv) customization into required size and shape.

### 2.3 Effect of Ru SAs on Light Absorption and Charge Kinetics

To investigate the reason for the excellent performances of R1.00, the light absorption, charge separation/transfer, and surface redox reactions of R1.00 were explored by various state‐of‐art characterizations. Initially, the light absorption abilities of all the as prepared catalysts were explored by UV–vis diffuse reflectance spectroscopy. According to the results, all the Ru loaded catalysts (R0.25, R0.50, R1.00 and R2.00) exhibit the significantly raised light absorption in the range of ∼450–800 nm, compared to R0.00 without Ru loading (Figure S20a). Notably, the band gaps of all the catalysts are reduced from 2.28 to 1.88 eV by increasing the loading of Ru elements from 0.25 to 1.00 wt%. This is attributed to the anchoring of Ru atoms onto the ZnIn_2_S_4_, resulting in the formation of Ru‐S bond and the reduced band gap of ZnIn_2_S_4_. Also, the as‐prepared catalysts exhibit the significant color change from light orange for R0.00 to dark green for R2.00. In fact, the catalyst colors become darker by increasing the Ru loading from 0.25 to 2 wt%. To investigate whether the improved light absorption by R1.00 in the range of ∼500–800 nm could boost the photocatalytic PP conversion, two 540‐nm and 680‐nm light emitting diode (LED) instead of xenon light were used (Figure S20b). The obtained results reveal that although significant absorption increase is observed between 500 and 800 nm for R1.00, this increase causes very limited contribution to enhance the photocatalytic activity [44, 45]. The conduction band (CB) and valence band (VB) edge positions of R0.00 and R1.00 are estimated by Mott‐Schottky analysis (Figure S21a,b). The flat band potentials for R0.00 and R1.00 are −0.75 and −1.1 V vs. the Ag/AgCl electrode (Figure S21a,b), corresponding to −0.12 and −0.47 V vs. the reversible hydrogen electrode (RHE), respectively. It ought to be noted that the CB edge position of a typical n‐type semiconductor is ∼0.2 V more negative than its Fermi level. Thus, the CB edge positions of R0.00 and R1.00 catalysts are estimated as −0.32 and −0.67 V vs. RHE, respectively. According to the band gap values of R0.00 (2.38 eV) and R1.00 (1.88 eV) exhibited in Figure S21c,d, the valence band (VB) position of R0.00 and R1.00 are calculated to be +2.06 and +1.21 V vs. RHE, respectively. Finally, the CB and VB edge positions of R0.00 and R1.00 are presented in Figure S21e,f.

**FIGURE 4:**
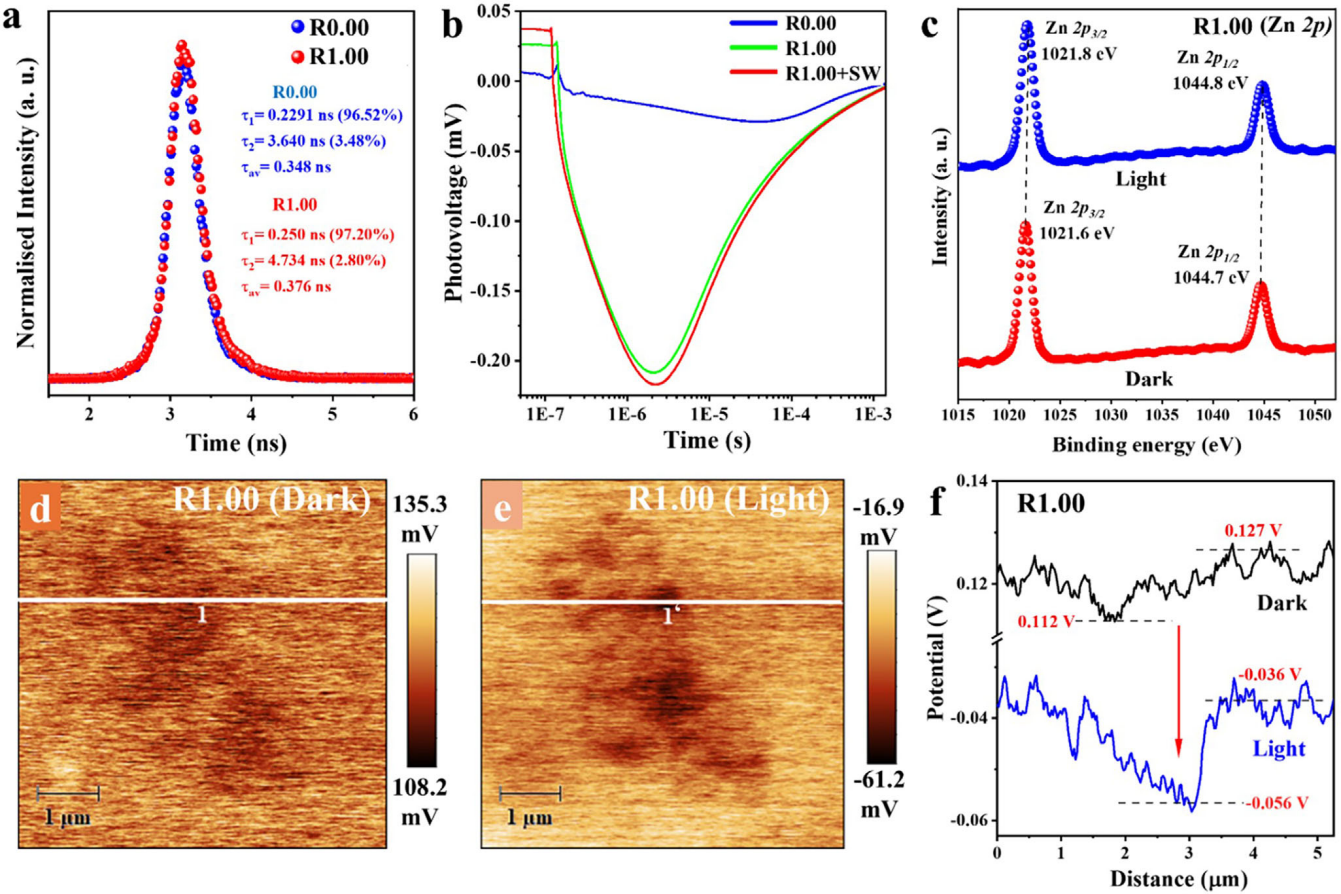
(a) TP spectra of R0.00 and R1.00. (b) TPV spectra of R.00, R1.00 and R1.00+SW. In situ high‐Resolution XPS spectra of (c) Zn 2p for R1.00 with light on and off, respectively. (d,e) AFM‐KPFM images of R1.00 along with (f) the corresponding line analysis of potential signals for R1.00 in dark and under light irradiation.

The photo‐generated charge separation/transfer in photocatalysts is explored by various ex/in situ and time‐resolved characterizations. The steady‐state photoluminescence (PL) spectra of R1.00 (Figure S22) reveals significant suppression of photo‐excited charge recombination in R1.00 compared to R0.00, owing to the obvious reduction on the PL peak intensity of R1.00 compared to that of R0.00. Figure 4a exhibits the transient‐state photoluminescence (TP) decay spectra of R.00 and R1.00. The slower decay of the PL spectrum, as well as the longer average charge carrier lifetime (τ_av_ = 0.376 ns) of R1.00 compared to that of R0.00 (τ_av_ = 0.348 ns), corroborate the effective repression of electron‐hole recombination by Ru SAs in R1.00 [15, 61]. Additionally, the transient‐state photovoltage (TPV) measurement was conducted to provide time‐resolved information about the dissociation, transfer, and recombination of photo‐excited charges on the surface of R0.00 and R1.00. Overall, R1.00 exhibits a significantly more negative SPV signal compared to R0.00 (Figure 4b), revealing that more photo‐generated electrons than holes migrate from the bulk to the surface of R1.00. The latter results clearly confirm the key role of Ru SAs in raising the extraction and transfer of photo‐generated electrons to the surface of R1.00. This increases the lifetime of photo‐excited electrons/holes through strong extraction and pumping of electrons by the Ru SAs. A detailed kinetic analysis further highlights the superior charge extraction capability of R1.00. Pristine R0.00 exhibits a slow decay to the most negative SPV signal (∼−0.0291 mV) at 40.549 µs, indicating sluggish electron transport from the bulk to the surface. This is followed by a rapid SPV decay to zero at 1.4 ms, owing to rapid electron‐hole recombination. In contrast, R1.00 exhibits a sharp decline to a much more negative SPV signal (−0.2087 mV) within ∼2.2111 µs, indicating substantially accelerated electron extraction regulated by Ru SAs. This is followed by a notably slower positive SPV decay to zero at 1.4 ms, further confirming the prolonged charge lifetime. Quantitatively, the Ru SAs enhance electron extraction efficiency by ∼18.34 times and accelerate charge separation by 7.2 times for R1.00, compared to that of R0.00. These results unequivocally validate the key role of Ru SAs in optimizing charge kinetics, thereby contributing to the superior photocatalytic performance of R1.00.

The electron transfer pathway was investigated by in situ XPS analysis in dark and under light irradiation. As exhibited in Figure 4c and Figure S23a,b, noticeable movements toward higher binding energy direction are observed for Zn 2p, In 3d, and S 2p peaks of R1.00, respectively. The high‐Resolution Ru 3d XPS spectrum for R1.00 overlaps with the C 1s peak [62, 63, 64, 65, 66]. Thus, the analysis is conducted for the Ru 3p peak of R1.00. As revealed by the ICP‐AES analysis, low Ru concentration (0.816 wt%) results in a very weak Ru 3p peak (Figure S23c). However, in situ XPS analysis (Figure S23c) clearly exhibit a movement to lower binding energy direction for the Ru 3p_3/2_ peak from 462.3 to 461.7 eV, indicating electron transfer from ZnIn_2_S_4_ NSs to Ru SAs [67, 68, 69, 70]. Since Ru SAs serve as the primary electron‐accepting sites compared to Zn, In, and S atoms on the surface, these results strongly support the claim that Ru SAs act as electron pumps, facilitating charge extraction from R0.00, while photo‐excited holes remain localized within the ZnIn_2_S_4_ framework. The results obtained are in good agreement with the results on transient SPV and EXAFS.

Then, in situ atomic force microscopy‐kelvin probe force microscopy (AFM‐KPFM) was conducted to reveal the distribution of photo‐excited electron/hole on the surface of R1.00 in the dark and under light irradiation. Figure S24a–d exhibits the AFM images and corresponding height profiles of R1.00 in the dark and under light irradiation, respectively. No obvious alteration of topographical morphology is found from the height profiles for R1.00 (Figure S24b,d). These results clearly confirm that any potential changes observed for R1.00 in the dark and under light irradiation purely arise from the differences of its surface charge distribution under these two conditions. Figure 4e–g exhibits the corresponding surface potential maps and line analysis in the same region for R1.00 in the dark and under light irradiation, respectively. As exhibited in Figure 4g, the surface potential is significantly reduced from +0.112 to −0.056 V in the negative region after light irradiation. In addition, compared to that in the dark condition, a significantly more negative surface potential under light irradiation is clearly exhibited for R1.00 in the 3D maps of surface potentials (Figure S25). Interestingly, a comprehensive exploration was conducted to investigate the impact of oxygen (O_2_) in the air on charge distribution. Thus, AFM‐KPFM analysis (Figure S26a–d) is conducted for R1.00 in air and nitrogen (N_2_) atmospheres, respectively, in the dark condition. The presence of a more negative potential in air (with the presence of O_2_ molecules) is clearly confirmed by a significant reduction in surface potential from −0.98 V (in N_2_) to −1.421 V (in air), as revealed by Figure S26c. The reason is that the O_2_ molecules from air adsorbed on the surface of R1.00 boost the attraction of more electrons to the surface of R1.00, resulting in the apparent reduction of surface potential. Moreover, Figure S24 presents the transient photocurrent (TPC) measurements of R0.00 and R1.00. R0.00 exhibits a TPC density value of ∼2.29 µA/cm^2^ under light irradiation, which is gradually reduced to ∼1.06 µA/cm^2^ within 60 s. In contrast, R1.00 exhibits a higher TPC density of ∼8.73 µA/cm^2^ under light irradiation compared to R0.00 (Figure S27), indicating that Ru SAs play a key role in effectively reducing photo‐excited electron‐hole recombination in R1.00. The gradual reduction of TPC density could be caused by various reasons, for example, surface blocking by reaction intermediates, formation of light activated trap, and early‐stage photo‐corrosion [71, 72, 73]. Moreover, the reduction of TPC density has been extensively reported for semiconductor photoelectrodes, owing to capacitive charging, electrolyte wetting, and surface reconstruction before reaching a steady‐state value [74, 75, 76, 77]. These collective results provide compelling evidence that Ru SAs act as efficient electron mediators, thereby significantly enhancing charge separation and photocatalytic activity of R1.00.

### 2.4 Effect of Seawater on Charge Kinetics

The undeniable positive impact of ionic species in seawater (e.g., Cl^−^ and Na^+^) has been reported and confirmed by state‐of‐art analytical techniques in recent studies [66]. Generally, ionic species in seawater can accelerate reaction kinetics and eventually improve catalytic efficiency. Particularly, Cl^−^ ions could react with photo‐generated holes (h^+^) or hydroxyl radicals (∙OH) through the following reaction pathways: i) 2Cl^−^ + 2h^+^→ Cl_2_; ii) 2Cl^−^ + 2∙OH + 2H^+^→ Cl_2_ + 2H_2_O. The resulting Cl_2_ then reacts with water to form hypochlorous acid (HClO; Cl_2_ + H_2_O → H^+^ + Cl^−^+ HClO), which readily decomposes into Cl^−^ with light irradiation (2HClO $\overset{\mathrm{hv}}{\to }$ 2H^+^ + 2Cl^−^ + O_2_). These redox interactions indicate that Cl^−^ ions mitigate charge recombination by interacting with photo‐generated holes, while simultaneously promoting electron extraction by Ru SAs. The synergistic effect of Cl^−^, mediated hole interactions and the exceptional electron‐extraction ability of Ru SAs are utilized to maximize charge separation and transfer, thereby accelerating photocatalytic performance. This hypothesis is systematically examined by in situ TP, TPV, XPS and electron paramagnetic resonance (EPR) analyses.

To explore the impact of seawater on photocatalytic performance, the concentrations of major ions in seawater were first analyzed, for example, sodium (Na^+^), sulphate (SO_4_^2^^−^), and chloride (Cl^−^). The results exhibit that the highest concentration (4.0 mg L^−^^1^) for Cl^−^, followed by SO_4_^2^^−^ (0.8 mg L^−^^1^) and Na^+^ (0.1 mg L^−^^1^). In comparison, calcium (Ca^2+^), magnesium (Mg^2+^), and potassium (K^+^) ions exhibit much lower concentrations, with ∼0.05 mg L^−^^1^ for each of the above ions. Overall, the ion concentrations on the seawater are as follows: Cl^−^ > SO_4_^2^^−^ > Na^+^ > K^+^ ≈ Mg^2^^+^ ≈ Ca^2^^+^. Furthermore, electrophoretic light scattering results (Figure S28) exhibit that the catalyst zeta potential is changed from −12 mV in Milli‐Q water to −21 mV in natural diluted seawater, and further to −30 to −40 mV in the presence of single‐salt electrolytes (NaCl: −30.5 mV, KCl: −30.9 mV, LiCl: −36.0 mV, Na_2_SO_4_: −37.6 mV, and NaNO_3_: −40.0 mV). These ion‐dependent negative shift reveal an apparent reorganization of the electrical double layer and enhanced interfacial polarization induced by seawater ions. The observed variation among different electrolytes, particularly the stronger negative shift in the presence of Li^+^, SO_4_^2^^−^ and NO_3_^−^, indicates the involvement of specific‐ion effects, for example, hofmeister‐type interactions and/or preferential anion adsorption within the helmholtz layer, rather than a simple ionic‐strength‐driven screening effect. Furthermore, this observed electrolyte‐induced interfacial polarization is consistent with previous reports on ion‐mediated enhancement of charge separation in seawater‐assisted photocatalytic systems and provide direct experimental support for the polarization‐driven mechanism raised in this work [66, 78, 79, 80, 81].

**FIGURE 5:**
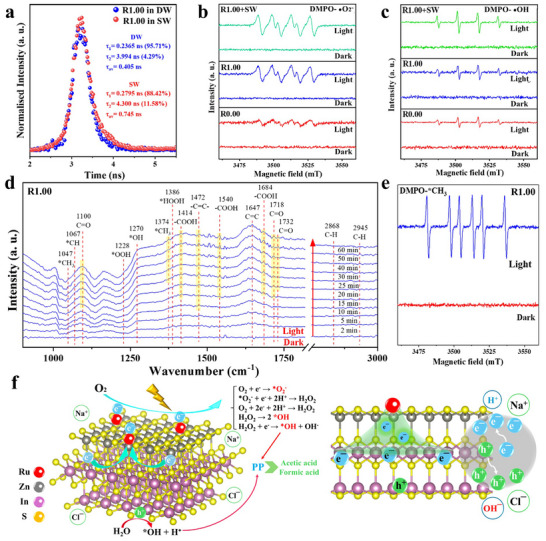
(a) In situ TP spectra results for investigating the effect of seawater on charge kinetics of R1.00. In situ EPR spectra of (b) DMPO‐∙OH and (c) DMPO‐∙O_2_^−^. (d) In situ IR spectra of photocatalytic reforming PP over R1.00 in the presence of PP and in air. (e) In situ EPR spectra for DMPO‐CH_3_ in dark and under light irradiation. (f) Reaction mechanism over R1.00 for photocatalytic upcycling of PP to value‐added chemicals (Left: overall and Right: effect of Ru SAs and seawater cations/anions in e^−^/h^+^ separation).

A pivotal characterization technique to validate the impact of seawater on charge kinetics is in situ TP. As exhibited in Figure 5a, in situ TP decay spectra of R1.00 are analyzed in both deionized water (DW) and seawater (SW) environments. The decay curves (Figure 5a inset) exhibit two distinct lifetime components. The short‐lived component (τ_1_), attributed to intrinsic exciton recombination within the bulk of R1.00, exhibits a minor increase in SW (τ_1_ = 0.2795 ns) compared to that in DW (τ_1_ = 0.2365 ns). This slight enhancement is attributed to the local electric field generated by the cations and anions from SW, which primarily influences charge kinetics in the bulk region rather than the surface. However, the long lifetime component (τ_2_) is more obviously affected by the solution environment of DW (τ_2_ = 3.994 ns; 4.29%) or SW (τ_2_ = 4.300 ns; 11.58%). In fact, the second decay is attributed to electron‐hole recombination, which is significantly affected by the local electric field near the surface of R1.00, owing to the cations and anions from SW. The prolonged second decay (τ_2_) in the presence of SW significantly raises the average charge lifetime from τ_av_ = 0.405 ns in DW to τ_av_ = 0.745 ns in SW for R1.00 (Figure 5a). The electrolyte‐assisted charge polarization effect of SW results in a remarkable raise on the average charge lifetime by 84% for R1.00, compared to that in DW. The influence of SW on charge separation is further corroborated by in situ TPV measurements. R1.00 exhibits a more pronounced negative SPV signal in SW compared to that in DW, confirming enhanced electron‐hole separation/migration facilitated by electrolyte interactions (Figure 4b). Particularly, R1.00 in SW reaches a negative SPV signal of −0.2178 mV at 2.2111 µs. In comparison, R1.00 in DW exhibits a slightly less negative signal of −0.2087 mV at 2.2111 µs. This 4.4% improvement in the negative SPV signal highlights the impact of SW on charge polarization and charge kinetics.

Extra in situ XPS tests were performed on R1.00 and seawater‐immersed R1.00 (R1.00‐SW) to investigate the impact of seawater on the electronic and surface characteristics of R1.00 under dark and light irradiation. The results in Figure S29a,b reveal the apparent movement (∼0.4 to 0.5 eV) of S 2p and Zn 2p peaks for R1.00‐SW under light irradiation, compared to those under dark condition. In contrast, the movement (∼0.1 to 0.2 eV) of S 2p and Zn 2p peaks for R1.00 are less apparent under light irradiation, compared with those under dark conditions (Figure S29c,d). Considering that the in situ XPS technique is sensitive to both surface potential variations (e.g., band bending) and chemical‐state changes, the more apparent peak movements for R1.00‐SW probably arise from synergistic electrostatic and chemical effects induced by dissolved ions. Specifically, ion adsorption at the solid‐liquid interface and light‐assisted surface reactions collectively varies the near‐surface electronic environment. Under light irradiation, the presence of electrolytes enhances interfacial carrier dissociation and modifies the charge extraction/recombination kinetics, resulting in increased photo‐induced local potential difference. These are reflected by the more apparent peak movements for R1.00‐SW, compared to those for R1.00, as revealed by in situ XPS test results [82, 83, 84, 85, 86].

To further validate the role of SW in accelerating charge separation, in situ EPR measurements were conducted in the photocatalytic conversion of raw PP plastic utilizing R1.00. The primary objective is to detect the generation of reactive oxygen species (ROSs), for example, superoxide radical (∙O_2_^−^) and ∙OH. As exhibited in Figure 5b,c, neither R0.00 nor R1.00 exhibits any DMPO‐∙O_2_^−^ or DMPO‐∙OH signal in the dark condition. However, after 9 min of light irradiation, R1.00 in seawater (R1.00 + SW) exhibits the apparently stronger DMPO‐∙O_2_^−^ and DMPO‐∙OH signals than R1.00 without SW, which in turn exhibits higher radical signals than pristine R1.00. These results reveal that SW can obviously enhance the efficiency of charge separation and radical generation in R1.00. This enhancement is attributed to the synergistic effect of Ru SAs and the electrolyte‐assisted charge polarization effect of SW, which collectively facilitate efficient electron extraction and promote robust electron‐hole separation under light irradiation.

### 2.5 Reaction Mechanism

The surface reaction pathway is investigated by in situ infrared (IR) spectroscopy. As exhibited in Figure 5d, in the presence of air, R1.00 exhibits the three peaks at 1047, 1270, and 1718 cm^−1^, attributed to the CH_3_ rocking, OH bending, and C = O stretching of acetic acid, respectively [15, 28, 29, 30]. From 0 to 60 min light irradiation, the intensities of these three peaks are raised gradually, particularly after 20 min light irradiation. However, it should be noted that the peak at 1047 cm^−^^1^ varies because the *CH species forms only in very low and short‐lived amounts, so its signal sometimes falls below detection. This region (1000–1100 cm^−^^1^) is also crowded with other vibrations (e.g., *CH_3_ at 1067 cm^−1^ and C‐O at 1100 cm^−1^), which can mask a weak peak. In addition, the intermediate (*CH_3_) is involved in a dynamic adsorption–desorption process, so its surface concentration naturally fluctuates during the reaction [87, 88, 89]. Notably, the peak at 1718 cm^−1^ is attributed to the C = O stretching of aldehyde intermediates, obtained in the presence of R1.00 and air [90]. Furthermore, by increasing the irradiation time from 0 to 60 min, two peaks (1067 and 1732 cm^−1^) exhibit a significant rise in their intensities. The latter peaks correspond to the CH bending and C = O stretching of formic acid, respectively. Particularly, the peak at 1732 cm^−1^ exhibits the presence of carboxyl groups in aldehydes and esters [30]. The above results clearly reveal that acetic acid and formic acid are generated on R1.00 in air within 60 min irradiation. Moreover, the peaks at 1414 and 1540 cm^−1^ are attributed to the symmetric and asymmetric COOH stretching, which confirms the generation of *COOH intermediates [28]. A distinct peak at 1647 cm^−1^ along with a peak at 1684 cm^−1^, are attributed to C = C stretching of unsaturated alkyl intermediates and carboxylic groups (‐COOH) from substrate adsorption [55]. Figure 5d also exhibits two relatively weak bands at 1228 and 1388 cm^−1^, attributed to the O‐O stretching mode of surface adsorbed superoxide (*OOH) and the OOH bending mode of surface adsorbed hydroperoxide (*HOOH), respectively [15, 91, 92, 93]. In addition, a gradual reduction in absorption intensities at 2874 and 2937 cm^−1^ (Figure 5d), is attributed to the asymmetric and symmetric C‐H stretching vibration of the ‐CH_2_‐ group, respectively [30, 94]. Also, a peak at 1374 cm^−1^, is assigned to the weak symmetric deformation of the ‐CH_3_ group [30, 95].

To further elucidate the critical role of Ru SAs and O_2_ in the reaction process, comparative in situ IR exploration were conducted under controlled conditions. As exhibited in Figure S30a,b, R0.00 in air primarily exhibits a peak at 1685 cm^−1^, attributed to the C = C stretching of unsaturated alkyl intermediates and carboxyl groups. Notably, the absence of peaks in the 1700–1750 cm^−1^ region of the R1.00 Argon (Ar) system highlights the key role of Ru SAs and O_2_ molecular in facilitating the formation of final oxidation products. While R0.00 in air exhibits a slight increase in *OOH peak intensity at 1220 cm^−1^ owing to peroxide group formation, R1.00 in the argon system exhibits no detectable peroxide species, reinforcing the importance of Ru SAs for promoting selective oxidation pathways. Furthermore, to better understand the role of R1.00 on the conversion of PP, the IR spectra of PP before and after the reaction were investigated. As exhibited in Figure S31, all PP plastic loaded catalysts before and after the reaction exhibit five peaks at 843, 1374, 1455, 2868, and 2949 cm^−1^, attributed to the C‐C, C‐H, ‐C = C‐, C = C, and C‐H bonds, respectively. Nevertheless, the residual PP after 12 h reaction exhibits several new peaks at 1110, 1641, and 3383 cm^−1^, corresponding to the carbohydrates, C = C stretching of alkenes/conjugated alkenes, and OH groups, respectively [30]. The intensities of these three new peaks are obviously raised after the reaction temperature increased to ∼ 67°C after 24 h reaction, and in the presence of R1.00. These results clearly reveal the formation of new bonding, owing to the conversion of PP and the generation of new products, for example, formic acid, acetic acid and other intermediate products. To further reveal the reaction mechanism, species quenching experiments were conducted to identify the key reactive species, including ∙OH, ∙O_2_^−^, hole (h^+^), and electron (e^−^) (Figure S32). The selective removal of h^+^ and ∙OH by ammonium oxalate and isopropanol, respectively, results in a drastic reduction in the evolution of formic acid by approximately two times and acetic acid by ∼1.4 times, compared to the evolution without any additions. These results confirm the primary role of h^+^ or ∙OH in the photocatalytic reaction. In comparison, when e^−^ and ∙O_2_^−^ were selectively scavenged utilizing silver nitrate and p‐benzoquinone, respectively, the evolution reduction for formic acid or acetic acid are less. These results reveal that e^−^/∙O_2_^−^ plays a less important role compared to h^+^/∙OH for the evolution of formic acid/acetic acid in the reaction. Overall, the importance of these species in this reaction is ranked as: ∙O_2_^−^ < e^−^ < ∙OH < h^+^.

On basis of all the above results, we raise a comprehensive photocatalytic mechanism (Figure 5e,f, Figures S33 and S34) for converting PP into value‐added chemicals over R1.00 in seawater and in air. This process is divided into four stages: (i) The full‐spectrum xenon light or concentrated natural sunlight irradiation, coupled with localized photothermal heating (∼67°C) facilitated by a reflective insulation layer, activates and partially degrades PP, initiating hydrogen atom transfer (HAT) [96, 97, 98]. The HAT process mediates interaction between the excited photocatalyst and hydrocarbon substrate, generating organic radicals, which subsequently react with O_2_ to yield peroxyl radical intermediates (Figures S33 and S34) [99, 100, 101]. In situ EPR results (Figure 5e) clearly confirm our hypothesis regarding the impact of HAT process in β‐scission of PP to methyl radical (·CH_3_), which is the key component in generation of final products, for example, formic acid. (ii) Ru SAs, in synergy with electrolyte‐assisted polarization from seawater, enhance charge carrier separation and extraction, with Ru SAs effectively trapping photo‐generated electrons while retaining holes on the catalyst surface. (iii) The generated charge carriers (e^−^/h^+^) promote the formation of ROS, including ∙OH and ∙O_2_^−^, which drive selective oxidation reactions (Figure 5f inset). (iv) These reactive species facilitate oxidation of PP‐derived radicals, resulting in the production of formic acid, acetic acid, and gaseous byproducts, for example, H_2_, CH_4_, C_2_H_4_, C_2_H_6_, and CO.

A comprehensive reaction pathway is introduced based on the raw PP conversion route through photocatalytic C‐H oxidation of the tertiary carbons in the plastic backbone (Figure S34), similar to the recent studies on photocatalytic conversion of polystyrene (PS) and polyethylene (PE) plastics conversion to value‐added chemicals [102, 103, 104]. In fact, the application of the HAT process and formation of the peroxyl radical intermediate are similar to the small molecule (‐CH_2_‐) oxidation. However, the absence of the second ‐H_2_‐ in the PP induces its C‐C bond cleavage, result in the degradation/oxidation products. In detail, PP consists of repeating units of CH_2_‐CH(CH_3_)‐. The HAT process absorbs the hydrogen from the tertiary carbon, resulting in the formation of a carbon cantered radical of CH_2_‐C*(CH_3_)‐. Since the experiment is performed in the presence of O_2_ from the air, the oxygen species (O*) could interact with the radical to produce an oxygen cantered intermediate (CH_2_‐C(O)‐CH_3_*), followed by β‐scission for C‐C bond cleavage, generating methyl radical (CH_3_*) and ketene (CH_2_ = C = O). On basis of on the quenching tests, ∙OH and h^+^ are the most influential factors in the photocatalytic conversion of raw PP to formic and acetic acids. Thus, two possible reaction pathways are proposed for formic acid generation as the main product. The CH_3_* undergoes multiple oxidation steps. Initially, CH_3_* reacts with O_2_ to form methyl peroxyl radicals (CH_3_O_2_*). Then, CH_3_O_2_* reacts with ∙OH to form methoxy radicals (CH_3_O*) [105, 106]. In fact, the CH_3_O_2_* is a common intermediate in oxidation reaction and ∙OH is a highly reactive species, which can react with CH_3_O_2_* to form CH_3_O* and HO_2_*. Followed by further oxidation of CH_3_O* to formaldehyde (HCHO). Finally, formic acid is generated either by oxidation or hydration of HCHO. Similarly, three possible pathways are raised for acetic acid generation in the presence of ∙OH and h^+^. Initially, ketene (CH_2_ = C = O) reacts with H_2_O to form CH_3_COOH directly. Second, CH_2_ = C = O generates an acetyl radical (CH_3_CO*) in the presence of O_2_. Then, CH_3_CO* reacts with ∙OH to generate acetic acid. Finally, ketene reacts with water in the presence of h^+^ to form CH_3_COOH. Further influence of ∙OH or h^+^ in the generation of other minor gas products, for example, H_2_, CO, CH_4_, C_2_H_4_, and C_2_H_6_, are reported in Figure S34.

The origin of the oxygen atoms involved in the formic acid and acetic acid generation are attributed to molecular O_2_ in air and H_2_O, as no other oxygen‐containing species are present in our reaction system. This conclusion is supported by multiple independent lines of experimental and analysis evidence: (i) The control experiments in the presence of argon (Ar) (Figure S10) led to production of only negligible amounts of acetic acid and formic acid, revealing that O_2_ in air is vital for C–C scission and subsequent oxygenation steps. (ii) The in situ EPR measurement (Figure 5e) confirmed the generation of methyl radicals (*CH_3_) under irradiation in the presence of air, seawater, and the catalyst, indicating that O_2_ plays a key role in stabilizing and transforming these radical intermediates. (iii) The radical‐scavenger experiments (Figure S32) exhibited that quenching •OH significantly reduce formic acid and acetic acid formation, while removal of other oxygenated radicals (e.g., •O_2_^−^) similarly decreases yields, highlighting the critical impact of reactive oxygen species derived from O_2_/H_2_O. (iv) The in situ IR spectroscopy results (Figure S30b) indicate that the characteristic C = O peaks at 1718 and 1732 cm^−1^, corresponding to formic acid and acetic acid species, appear only in the presence of O_2_ and completely disappear in Ar atmosphere, further confirming that oxygen uptake is essential for product generation. These results are fully consistent with high‐quality isotope‐labelling studies reported in the literature, where ^18^O_2_ and H_2_^18^O tracing clearly showed that the O atoms in formic acid, acetic acid, and related oxygenates originates from molecular O_2_ and/or H_2_O during photocatalytic oxidation of small hydrocarbons and model organics [107, 108]. Isotope labelling studies on TiO_2_ and related systems have similarly exhibited incorporation of ^18^O from O_2_ into key C_2_ intermediates such as oxalate [109, 110], while plastic photo‐reforming literature similarly confirms the central role of oxygenated species in product formation [15]. Thus, our experimental results and these mechanistic precedents provide strong evidence that O_2_ and H_2_O are the exclusive oxygen sources responsible for the formation of formic acid and acetic acid in our photocatalytic plastic conversion pathway.

In this work, only C_1_ and C_2_ products, including formic acid and acetic acid, were detected and no alcohols and aldehydes were obtained. The obtained product types can be explained by several factors related to the reaction environment and the chemical structure/degradation of PP, as follows: (i) Owing to the IR‐light absorption, the application of an insulation layer can increase the reaction temperature to ∼65°C. This raised reaction temperature (∼65°C) is one important reason for further oxidation of intermediates species (e.g., alcohol and aldehydes), which are typically short‐lived intermediate products, producing more stable products, for example, formic acid and acetic acid [111, 112]. (ii) The reaction was performed in the presence of air (aerobic environment). In fact, oxygen (O_2_) in the air can significantly enhance the oxidation (over‐oxidation) of intermediate species (e.g., methanol, ethanol, and aldehyde) to the more thermodynamically stable carboxylic acids (e.g., formic and acetic acid) products [113, 114]. (iii) The relatively long reaction times (e.g., 12 to 36 h) resulted in more oxidation (over‐oxidation) of reaction intermediates (e.g., alcohols and aldehydes) to C_1_ and C_2_ products [115]. (iv) The tertiary carbon centers of PP facilitate hydrogen‐abstraction (HAT) reaction pathway, which is suitable for initial C‐C scission into small radicals, resulting in the generation of C_1_ and C_2_ products instead of larger oxygenate molecules [116]. The produced small units undergo rapid oxidation to generate final stable products under aerobic conditions in the presence of Ru SAs.

## 3 Conclusion

To resolve the oceanic plastic waste pollution crisis, we have prepared the Ru single atoms (SAs) loaded ZnIn_2_S_4_ photocatalysts (ZnIn_2_S_4_/Ru SAs) in the forms of powder or floatable artificial leaf (AL) with high efficiency for the direct conversion of raw PP plastic into value‐added chemicals, by efficiently utilizing the whole spectrum of sunlight, seawater and air. The optimized ZnIn_2_S_4_/Ru SAs catalyst (R1.00) have achieved remarkable evolution amounts of 1151.8 µmol g^−1^ for liquid and gas products, with more than 65% selectivity to formic acid. Various advanced in situ characterizations were employed to elucidate the atomic‐level structure‐performance relationships and the reaction mechanism in realistic conditions. In situ X‐ray photoelectron spectroscopy, in situ atomic force microscopy‐kelvin probe force microscopy, and in situ electron paramagnetic resonance (EPR) reveal efficient photo‐electron extraction from ZnIn_2_S_4_ nanosheets to Ru SAs, followed by subsequent electron capture by O_2_ molecules in air. Additionally, transient‐state photoluminescence spectroscopy, transient photovoltage measurements, and EPR demonstrate that electrolyte‐assisted polarization (induced by cations and anions in seawater) significantly enhances charge separation and transfer. Finally, in situ EPR, in situ infrared (IR) spectroscopy, and quenching experiments confirm the pivotal roles of reactive oxygen species (∙O_2_^−^/∙OH) in oxidizing PP into valuable chemicals. These findings underscore the high potential of floatable AL photocatalytic platform for reforming plastic waste into high‐value chemicals, offering a scalable and sustainable solution to plastic pollution around the globe.

## 4 Experimental section

Initially, ZnIn_2_S_4_ NSs were prepared using a hydrothermal route. In the next step, a series of Ru single atoms (SAs) loaded photocatalysts (ZnIn_2_S_4_/Ru SAs) were prepared via a stirring method at 70°C for 4 h for direct conversion of raw polypropylene (PP) plastic mixed in seawater into valuable chemicals. The photocatalytic upcycling test of PP was conducted in a glass flask with sealed silicone rubber septa under atmospheric pressure. To maintain a specific reaction temperature (∼65°C), a reflective insulation layer is wrapped around the reactor, leaving the top side uncovered as the light window. A 300 W Xenon lamp (PLS‐SXE 300, Beijing Perfect Light) emitting full‐spectrum light was utilized as the light source. Seawater was collected from Glenelg Beach in Adelaide (South Australia). At specified intervals, 100 µL of headspace gas and 250 µL of the reaction solution were sampled from the reactor and analyzed using gas chromatography and high‐performance liquid chromatography (HPLC) to detect possible gaseous and liquid products, respectively. Finally, the floatable AL sample was prepared using the best prepared photocatalyst (R1.00) by the spray‐vacuum filtration approach to load the catalyst on glass fiber. For realistic outdoor tests, a light concentrator/magnifier was utilized to concentrate the natural sunlight on the photocatalyst surface and simultaneously enhance the reaction temperature to an acceptable level. Detailed experimental procedures are reported in the Supplementary Information.

## Conflicts of Interest

The authors declare no conflicts of interest.

## Supporting information




**Supporting File**: adma72812‐sup‐0001‐SuppMat.docx.

## Data Availability

The data that support the findings of this study are available from the corresponding author upon reasonable request.
