# Supplementary material for: Single‐Atom Photocatalyst as Floatable Artificial Leaf for Upcycling Oceanic Plastic Waste
Source: Adv Mater. 2026 Mar 17;38(21):e19931. doi: 10.1002/adma.202519931 (PMC13073120; doi:10.1002/adma.202519931)
Supplement: Supplementary file 1 — Supporting File: adma72812‐sup‐0001‐SuppMat.docx. [file ADMA-38-e19931-s001.docx]

Supplementary Information

**Single-Atom Photocatalyst as Floatable Artificial Leaf for Upcycling Oceanic Plastic Waste**

*Amin Talebian-Kiakalaieh^1^, Xin Xu^1^,* *Wenzhong Ji^2^, Yun Liu^2^, Bingquan Xia^3^, Jingrun Ran^1*^, Shi-Zhang Qiao^1*^*

^1^ School of Chemical Engineering, University of Adelaide, Adelaide, SA 5005, Australia

^2^ Research School of Chemistry, ANU College of Science, The Australian National University, Canberra, ACT 2601, Australia

^3^ Key Laboratory of Green Chemical Engineering Process of Ministry of Education, School of Chemistry and Environmental Engineering, Wuhan Institute of Technology, Wuhan 430074, Hubei, China

*Correspondence to: [jingrun.ran@adelaide.edu.au](mailto:jingrun.ran@adelaide.edu.au); [s.qiao@adelaide.edu.au](mailto:s.qiao@adelaide.edu.au)

**1. Materials**

**1.1. Preparation of ZnIn_2_S_4_ nanosheets (NSs)**

ZnIn_2_S_4_ NSs were synthesized using a hydrothermal route. Specifically, a mixture comprising 0.277 g of Zn(NO_3_)_2_·6H_2_O, 0.560 g of In(NO_3_)_3_·xH_2_O, and 0.559 g of thioacetamide was dissolved in 140 mL of deionized water under continuous stirring. Subsequently, 21.1 mL of 1.0 M HCl aqueous solution were added to the afore-mentioned solution. The resulting solution was then transferred into a 200 mL hydrothermal autoclave and maintained at a temperature of 160 °C for a duration of 12 h.

**1.2. Preparation of Ru single atoms (SAs) loaded on In_2_ZnS_4_ photocatalyst**

The fabrication of the Ru SAs loaded on ZnIn_2_S_4_ photocatalyst was achieved via a stirring method at 70 °C for 4 hours. Specifically, 100 mg of the synthesized ZnIn_2_S_4_ NSs were introduced into a vial, followed by the addition of 15.0 mL of deionized water. Then, the obtained mixture was sonicated (30 min) and stirred for 30 min before addition of required Ru aqueous solution. Afterward, required amount of Ru (RuCl_3_·xH_2_O) was dissolved in deionised water by sonication for 30 min, added to the afore-mentioned solution drop by drop under vigorous stirring. The resulting solution was subsequently stirred at 70 °C for about 4 hours and washed 5 times with deionized water and ethanol before drying overnight at room temperature.

**1.3.** **Preparation of Ru SAs loaded on ZnIn_2_S_4_ photocatalyst by hydrothermal method**

The Ru SAs loaded on ZnIn_2_S_4_ NSs were prepared by a hydrothermal route. Particularly, a mixture of 0.277 g Zn(NO_3_)_2_·6H_2_O, 0.560 g In(NO_3_)_3_·xH_2_O, 0.559 g thioacetamide and the required amount (1 wt%) of (RuCl_3_·xH_2_O) were dissolved in 140 mL deionized water under continuous stirring. Followed by addition of 21.1 mL 1.0 M HCl aqueous solution, the final solution was then transferred into a 200 mL hydrothermal autoclave and maintained at a temperature of 160 °C for a duration of 12 h. After the hydrothermal step, the obtained sample was washed 5 times with deionized water and ethanol before drying overnight at room temperature. The final sample was denoted as R1.00(HT).

**1.4.** **Preparation of Ru SAs loaded on ZnIn_2_S_4_ photocatalyst by photo-reduction method**

In a typical procedure of photoreduction loading of Ru on ZnIn_2_S_4_, 100 mg ZnIn_2_S_4_ photocatalyst was dispersed in 100 mL H_2_O and then required amount (1 wt%) of Ru (RuCl_3_·xH_2_O) was added to the aqueous solution under light irradiation and stirring for 1 h. After light irradiation for 1 h, the sample was filtered and washed 5 times by deionized water and ethanol before drying overnight at room temperature. The catalyst obtained was denoted as R1.00(PR).

**1.5. Preparation and testing of the floatable artificial leaf (AL) photocatalyst**

In this research, we used various types of filter papers including regular Watman filter paper, nylon and glass fibre filter papers. The floatability test of different filter papers (Figure S17a) exhibits that Watman filter papers sink right after contacting with water, owing to adsorbing water and significant increase in their weight. In contrast, the nylon and glass fibre filter papers float on the water surface, however, nylon possess a hydrophobic nature and made of plastic, which renders it an unqualified choice for our research. Finally, the glass fibre filter paper is chosen, because it’s light, hydrophilic and highly temperature resistant. To prepare the floatable AL sample, enough R1.00 catalyst is mixed with deionized water (5 ml), followed by sonication for ~1 hour before transferring to the spray nozzle. The spray-vacuum filtration approach is utilized for loading of catalyst on glass fibre, since it can provide a smooth and highly distributed catalyst surface on the filter paper (Figure S17b-d). For realistic outdoor tests, a light concentrator/magnifier (300 mm large diameter solar concentrator with a 125 mm focal length) is utilized to concentrate the natural sunlight on photocatalyst surface and simultaneously enhance the reaction temperature to the acceptable level. The test was performed for 5 h, from 9:30 am to 2:30 pm, on the 24th of January 2025 at the University of Adelaide with the maximum air temperature of 26 °C. Nevertheless, to obtain an average temperature of 65 °C during the test, a cooling water (15 °C) circulation system was utilized to avoid the over-heating of the whole reaction system. This obtained average temperature (65 °C) reflects the combined effect of concentrated sunlight irradiation and thermal accumulation in the reaction cell, simulating practical solar-concentrated photocatalytic conditions. To enhance contact between plastic particles and floatable AL (photocatalyst surface), continuous stirring (400 rpm) is applied. However, to avoid the formation of a stirring vortex during reaction, which can cause the AL to sink and result in the separation of plastic particles from AL surface, a stainless steel mesh is applied to eliminate stirring vortex and create a smooth stirring during the reaction (Figure S17g). The liquid products were analysed with HPLC and H-NMR.

**2. Characterizations**

Powder X-ray diffraction (XRD) patterns were acquired using a Miniflex X-ray diffractometer (Rigaku) equipped with a Cu Kα radiation source. Transmission electron microscopy (TEM), high-resolution transmission electron microscopy (HRTEM), high-angle annular dark-field scanning transmission electron microscopy (HAADF-STEM), energy-dispersive X-ray spectroscopy (EDX), and EDX elemental mapping images were obtained using FEI Titan S/TEM and FEI Tecani G2 Spirit TEM instruments (Thermo Fisher Scientific, USA). Atomic force microscopy (AFM) and Kelvin probe force microscopy (KPFM) images, along with corresponding height/potential profiles, were acquired using a dimension icon system (Bruker, USA). In addition, the AFM-KPFM analysis conducted in the air and N_2_ atmospheres, respectively, to investigate the impact of various environments on photo-generated electron/hole separation and transfer. Inductively coupled plasma atomic emission spectroscopy (ICP-AES) was conducted using an Optima 8000 ICP-OES instrument (Perkin Elmer). X-ray photoelectron spectroscopy (XPS) characterization was carried out on a K-Alpha plus XPS system (Thermo Fisher Scientific, USA), with photocatalysts excited by a light-emitting diode in in-situ XPS measurements. UV-Vis diffuse reflectance spectra and UV-Vis absorption spectra were obtained using a UV-Vis spectrophotometer (UV2600, Shimadzu, Japan). Steady-state photoluminescence (PL) spectra at room temperature were acquired using an RF-5301PC spectro-fluorophotometer (Shimadzu, Japan), while transient-state PL decay curves were obtained using an FLS1000 fluorescence lifetime spectrophotometer (Edinburgh Instruments, UK). In situ IR spectra were collected using a Nicolet iS20 spectrometer equipped with a HgCdTe (MCT) detector. Synchrotron-based X-ray absorption near-edge structure (XANES) and extended X-ray Absorption Fine Structure (EXAFS) measurements were conducted in an ultrahigh vacuum chamber of the undulator soft X-ray spectroscopy beamline at the Australian Synchrotron. Steady-state surface photovoltage (SPV) spectra were acquired using a home-built apparatus as described by Kang *et al.* ^[1]^, while transient-state SPV spectra were obtained using another device introduced in the referenced work. Electron paramagnetic resonance (EPR) spectra under visible light irradiation were obtained using an EPR spectrometer (Bruker model A300). The impact of various seawater ions was studied by the Electrophoretic light scattering (ELS) analysis (ZETA SIZER, Nano series).

3. **Photoelectrochemical and electrochemical measurements**

Surface charge transfer efficiencies were evaluated through transient photocurrent (TPC) measurements conducted on a CHI 660C electrochemical workstation employing a standard three-electrode system. ZnIn_2_S_4_ (R0.00) and R1.00 were coated onto F-doped SnO_2_-coated glass (FTO glass) to serve as the working electrodes, while a Pt wire and Ag/AgCl (saturated KCl) were employed as the counter electrode and reference electrode, respectively. The preparation of the working electrodes involved dispersing 10 mg of the catalyst along with 15 mg of poly(ethylene glycol) (PEG; molecular weight: 20000, Sigma-Aldrich) in 1 mL of ethanol. After sonication for 30 min to ensure uniform distribution, 50 µL of the solution were drop-cast onto a 0.7×1.6 cm^2^ FTO glass electrode using a pipette. The resulting electrode was then calcined at 350 °C in an argon atmosphere for 30 min in a short tube furnace. For TPC density measurements, 0.5 M Na_2_SO_4_ served as the electrolyte containing hole scavengers, which rapidly captured photo-induced holes on the surface of the catalysts, thus mitigating surface charge recombination. Consequently, the recorded TPC density provided insights into the charge separation efficiency within the bulk of the catalysts. Additionally, Mott-Schottky plots were generated using the same three-electrode system, employing the as-synthesized samples coated on FTO glass as the working electrodes in a 0.5 M Na_2_SO_4_ aqueous solution.

4. **Photocatalytic plastic upcycling**

The photocatalytic upcycling test of Polypropylene (PP) plastic was conducted in a glass flask with sealed silicone rubber septa under atmospheric pressure. To maintain a specific reaction temperature and ensure temperature stability throughout the reaction duration, a reflective insulation layer is wrapped around the reactor, leaving the top side uncovered as the light window. Temperature control was achieved by adjusting the intensity of a Xenon lamp, resulting in a reaction temperature of ~65 °C during the reaction period. After the reaction, the reactor was allowed to cool naturally to ambient temperature. A 300 W Xenon lamp (PLS-SXE 300, Beijing Perfect Light) emitting full-spectrum light was utilized as the light source. For each trial, 10 mg of photocatalyst and 20 mg of PP plastic were added into 10 mL of seawater and subjected to sonication for 30 min, followed by 30 min of purified air gas bubbling, prior to light irradiation for the designated duration. In case of real-world plastic waste tests, ~35 mg of straw, food container and bottle caps were applied in each test. Continuous stirring was maintained during irradiation. Seawater, collected from Glenelg Beach in Adelaide (South Australia), underwent basic filtration to remove sand and particulates before use. At specified intervals, 100 µL of headspace gas was sampled from the reactor and analyzed using gas chromatography (Agilent 8890) to detect possible gaseous products (C_1_-C_4_). Additionally, 250 µL of the reaction solution was withdrawn from the reactor, filtered using Millex syringe filters of 0.22 micrometers to remove photocatalyst and impurities, and then analyzed for substrate and product concentrations in the supernatant using high-performance liquid chromatography (HPLC) and 600 MHz NMR spectrometer (Bruker). Furthermore, a stability test was conducted over a duration of 36 hours.

$Selectivity to Formic acid=\frac{X \times n(Formic acid)}{\sum X\times n(all obtained products)}\times100\%$ (S1)

It is important to note that in the context of this experiment, X and n are referred to the number of the carbon atoms and moles of each product.


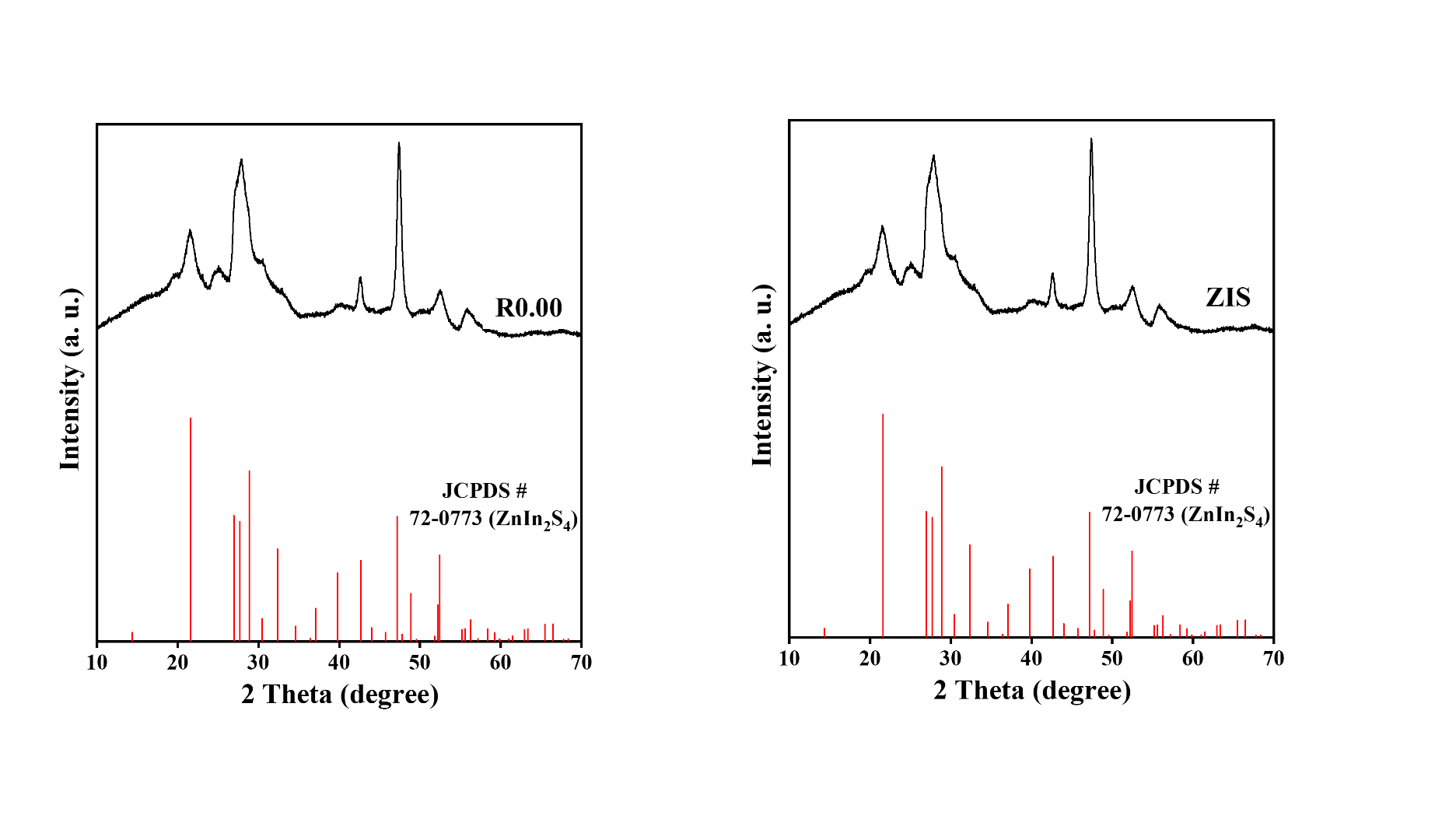


**Figure S1**. XRD pattern of ZnIn_2_S_4_ NSs (R0.00).


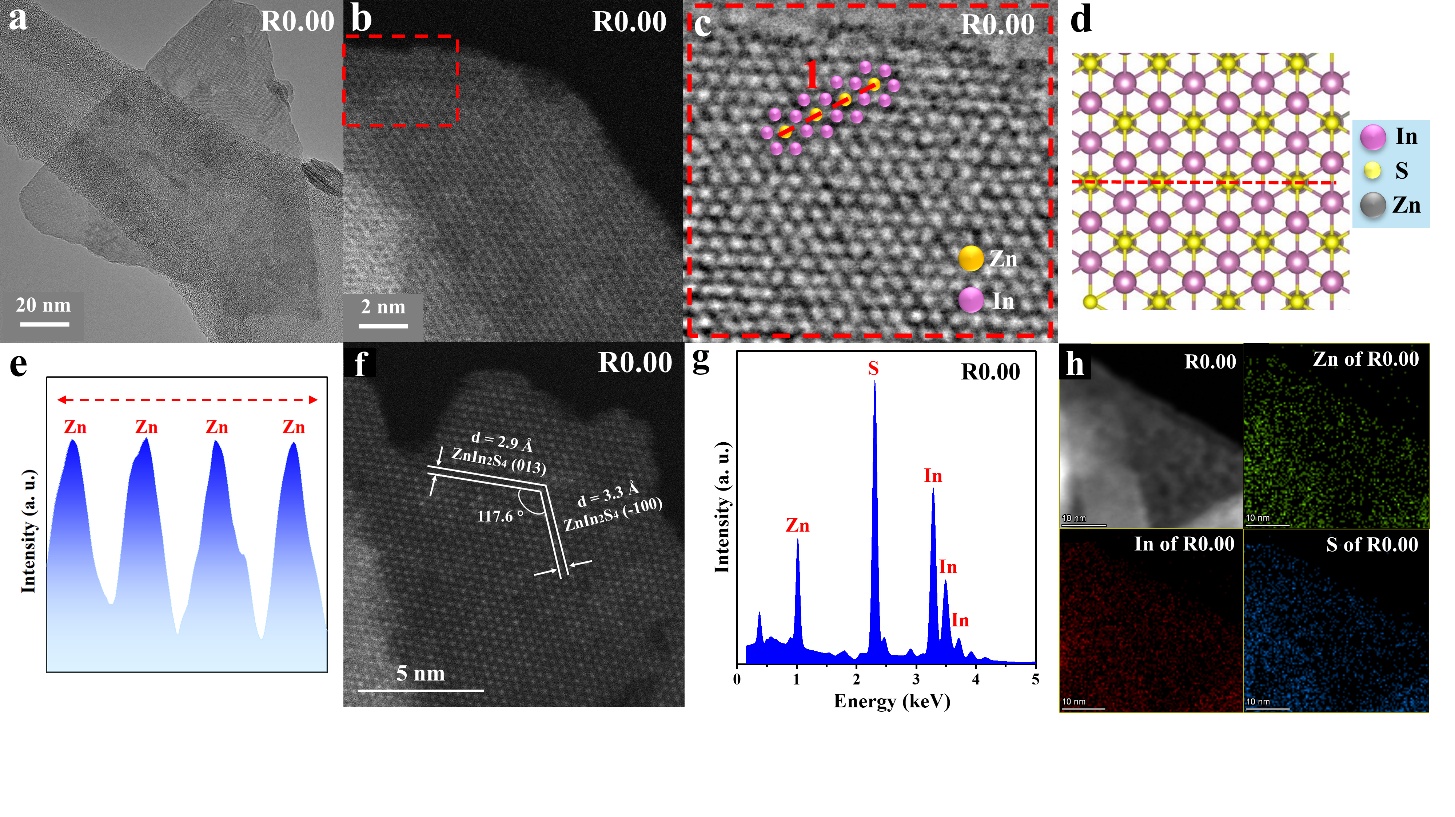


Figure S2. (a) TEM image, (b) Atomic-Resolution HAADF-STEM image and (c) DPC-STEM image of ZnIn_2_S_4_ (R0.00). (d) The modelled atomic structure of R0.00 and (e) the corresponding line analysis profile along line 1 in Figure S2c for R0.00. (f) Atomic-Resolution HAADF-STEM image, (g) EDX spectrum and (h) HAADF-STEM image and the corresponding elemental mapping images of Zn, In, and S for R0.00.


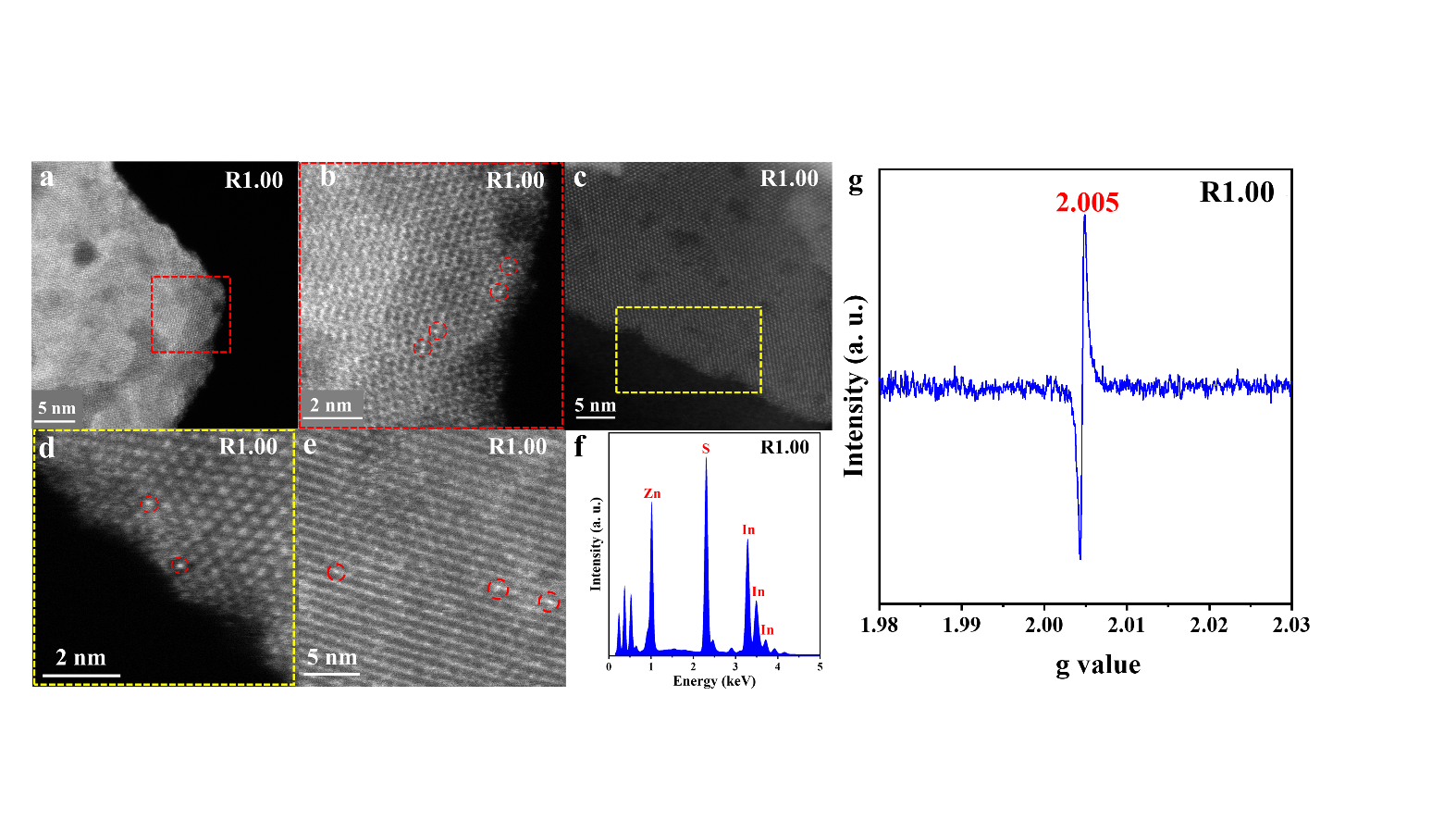


**Figure S3**. (a-e) Atomic-Resolution HAADF-STEM images of R1.00. Figure S3b, d and e exhibit the highlighted Ru SAs anchored on the surface of ZnIn_2_S_4_ NSs. (f) EDX spectrum and (g) EPR spectrum of R1.00.


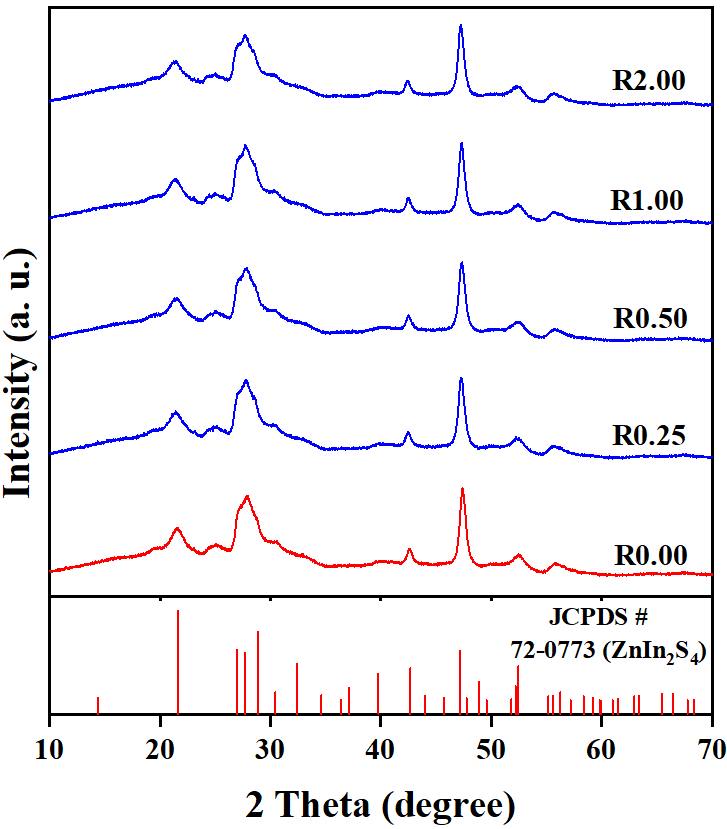


Figure S4. XRD patterns of R0.00, R0.25, R0.50, R1.00 and R2.00.


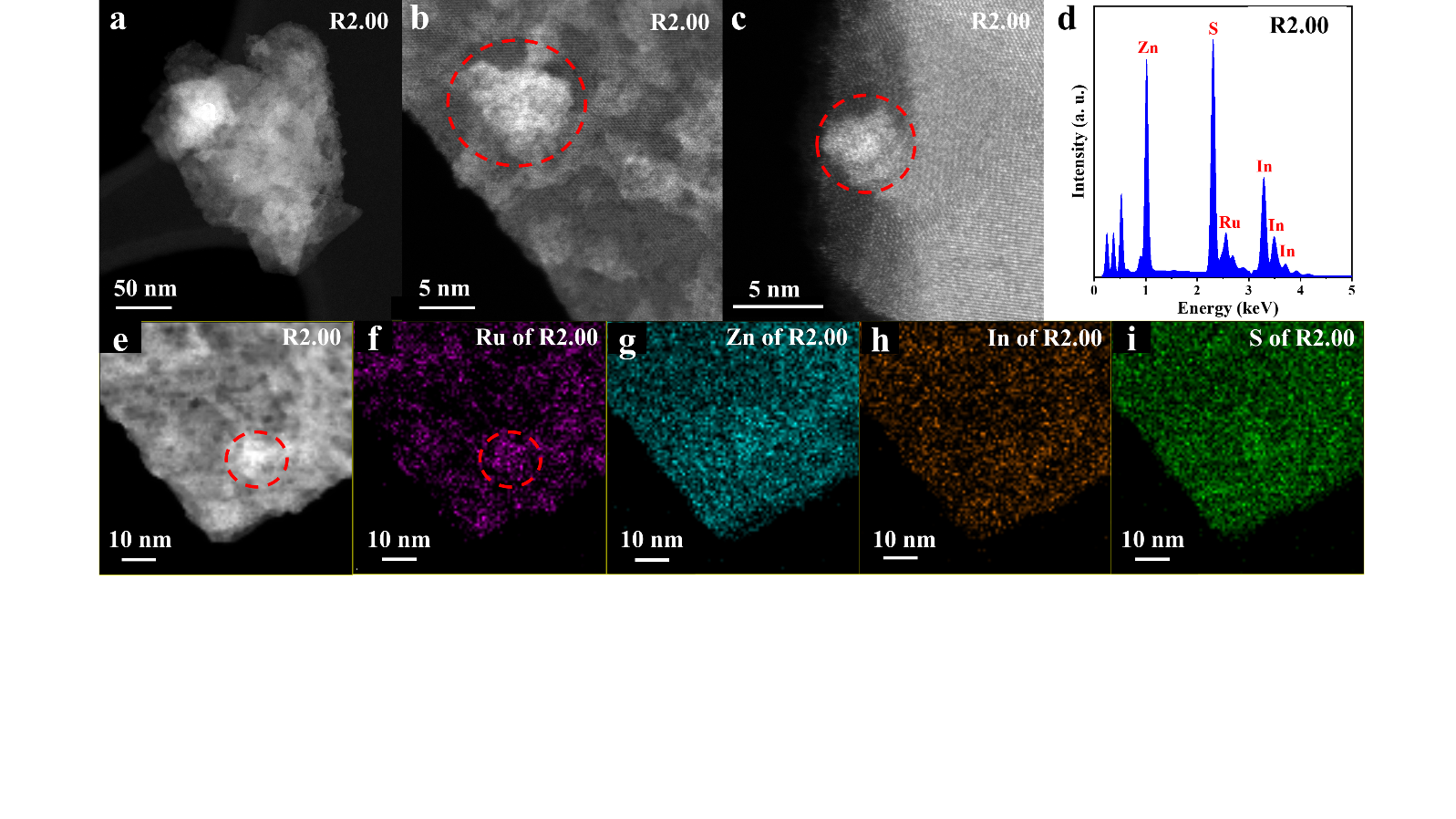


**Figure S5.** (a) HAADF-STEM image, (b-c) High-Resolution HAADF-STEM images of R2.00. (d) EDX spectrum of R2.00. (e) HAADF-STEM image and the corresponding elemental mapping images of (f) Ru, (g) Zn, (h) In, and (i) S for R2.00.


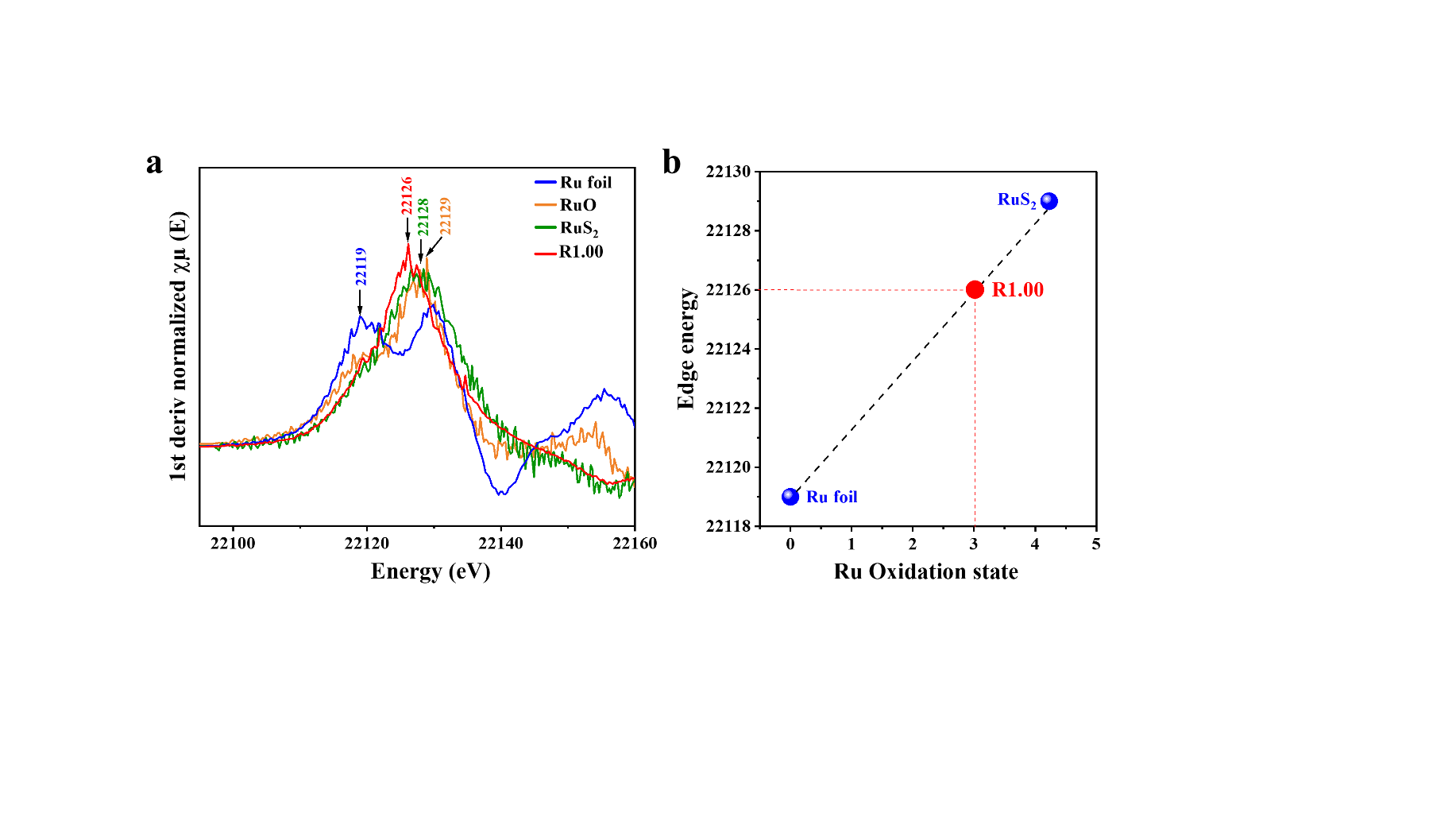


**Figure S6.** (a) Synchrotron-based Ru K-edge XANES spectra of R1.00, Ru foil, RuO and RuS_2_. (b) Ru oxidation state in R1.00 revealed by applying the absorption edge energy (E_0_).


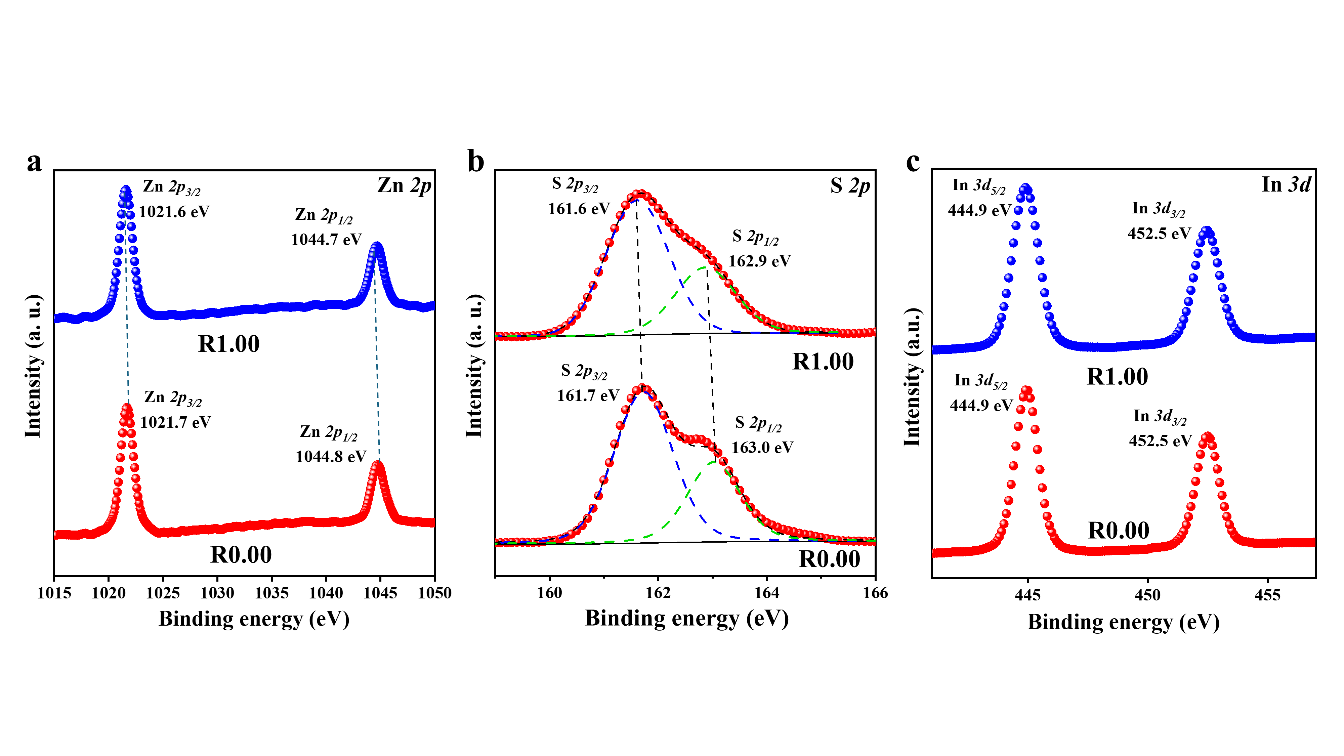


**Figure S7.** High-Resolution XPS spectra of (a) Zn 2p, (b) S 2p, and (c) In 3d for R1.00 and R0.0.


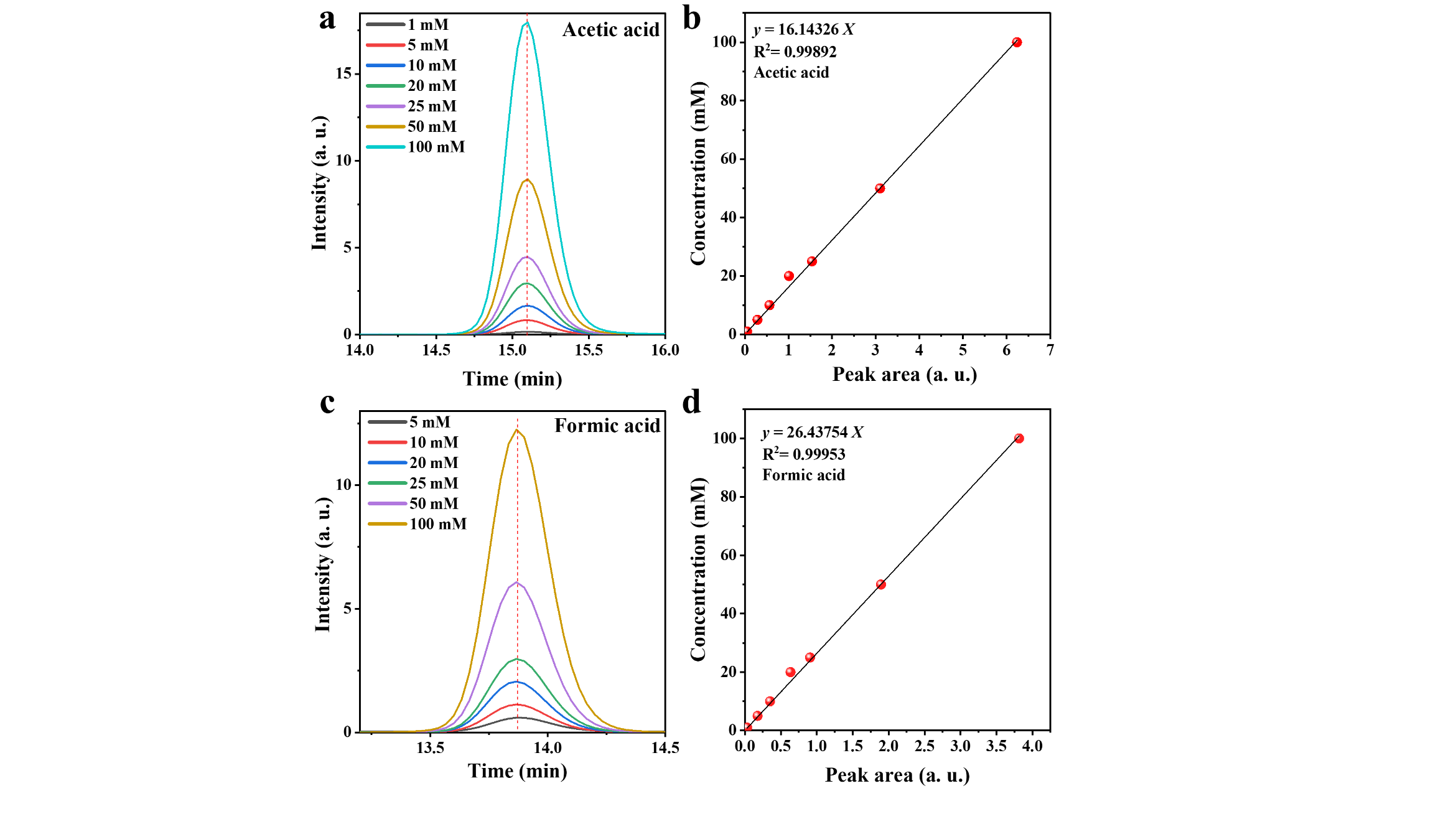


**Figure S8.** HPLC calibration curves for (a-b) acetic acid and (c-d) formic acid.


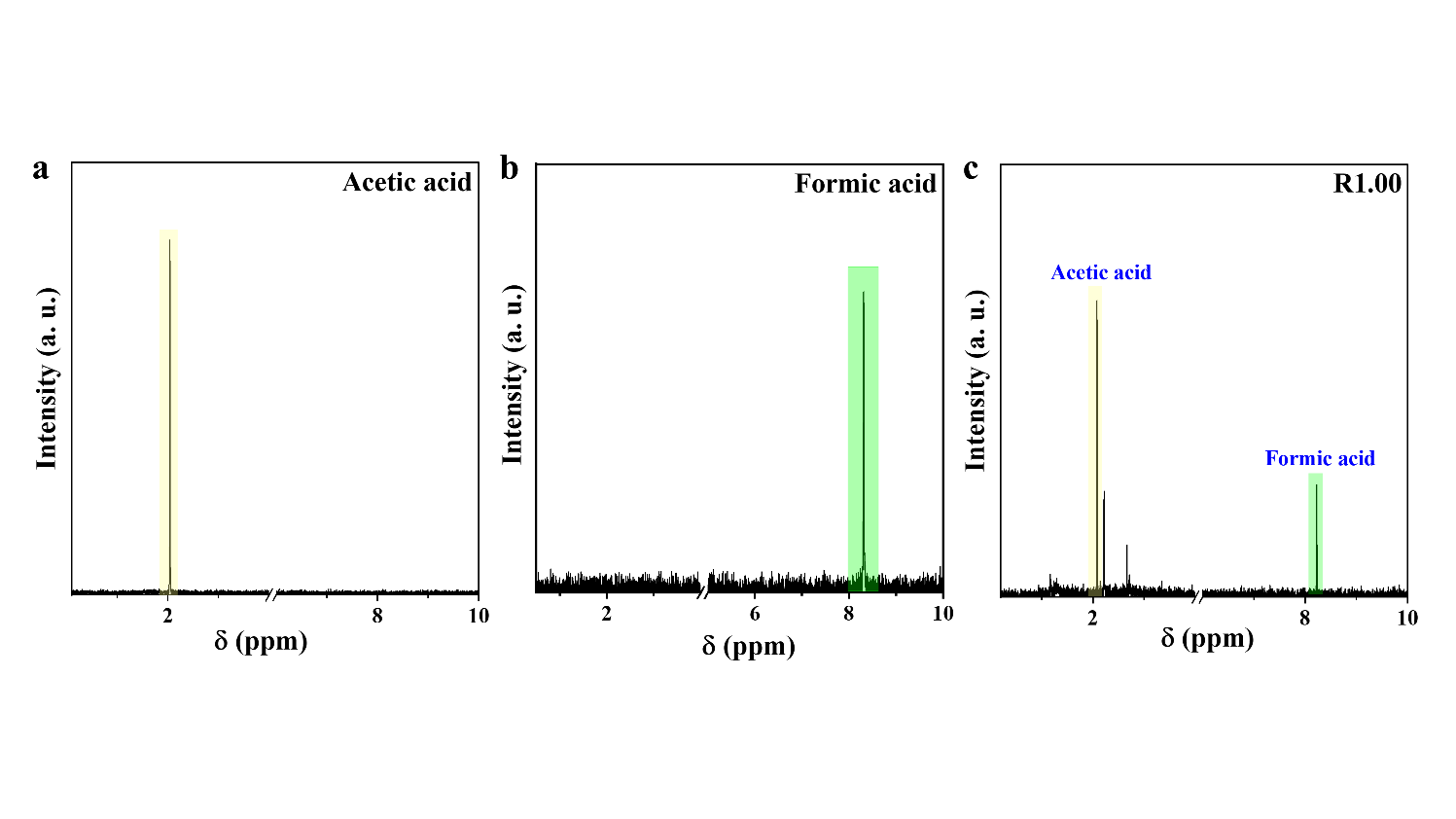


**Figure S9.** ^1^H NMR spectra for standard chemicals of (a) acetic acid and (b) formic acid, and (c) liquid products generated over R1.00 after 24 h.


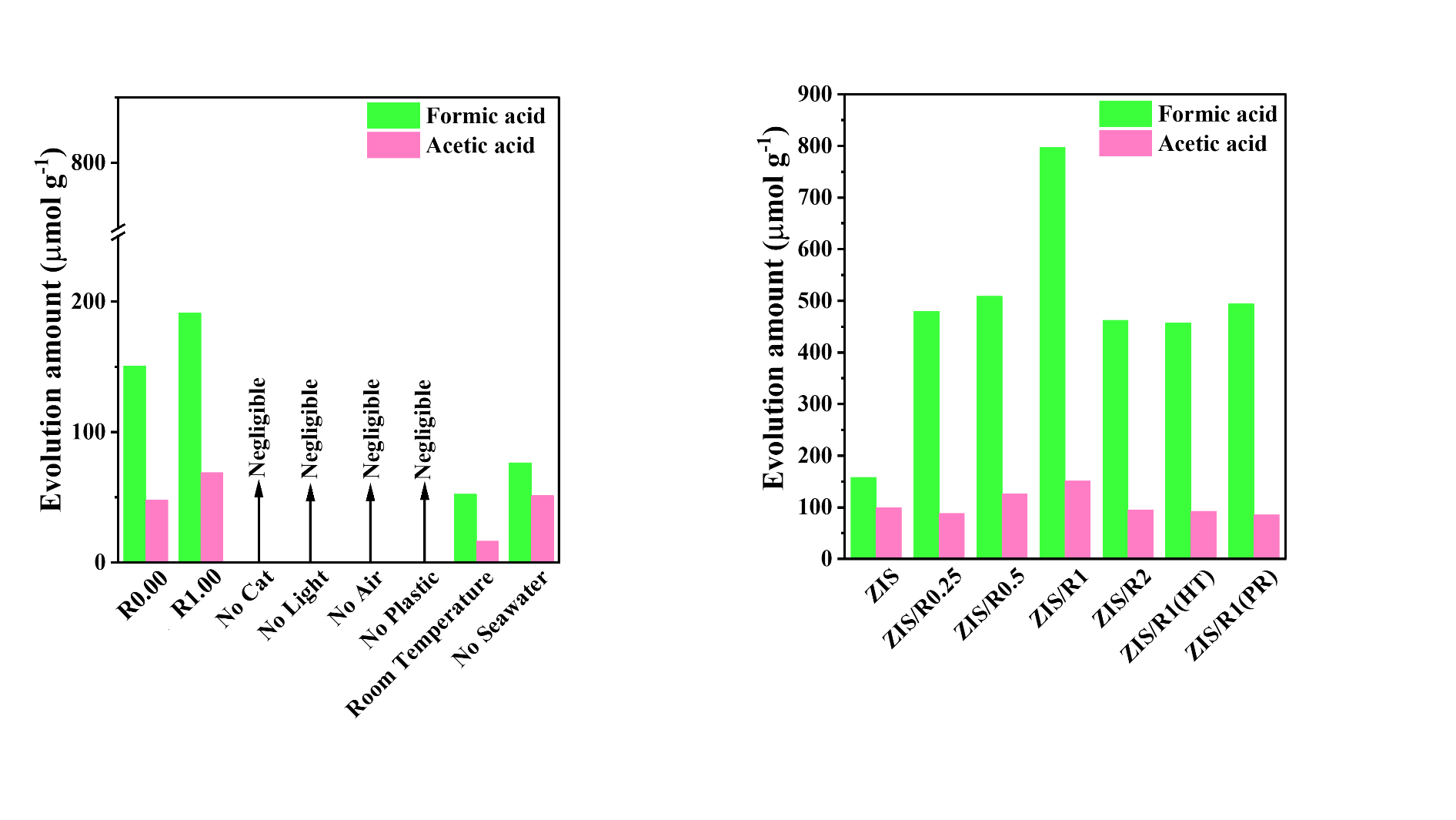


**Figure S10.** Results on control experiments of photocatalytic reforming raw PP using R1.00 with xenon light irradiation for 12 h. The above experiments were conducted in the absence of catalyst, light, air (in Argon), PP plastic, seawater (in deionized water) and at room temperature (without insulation layer), respectively.


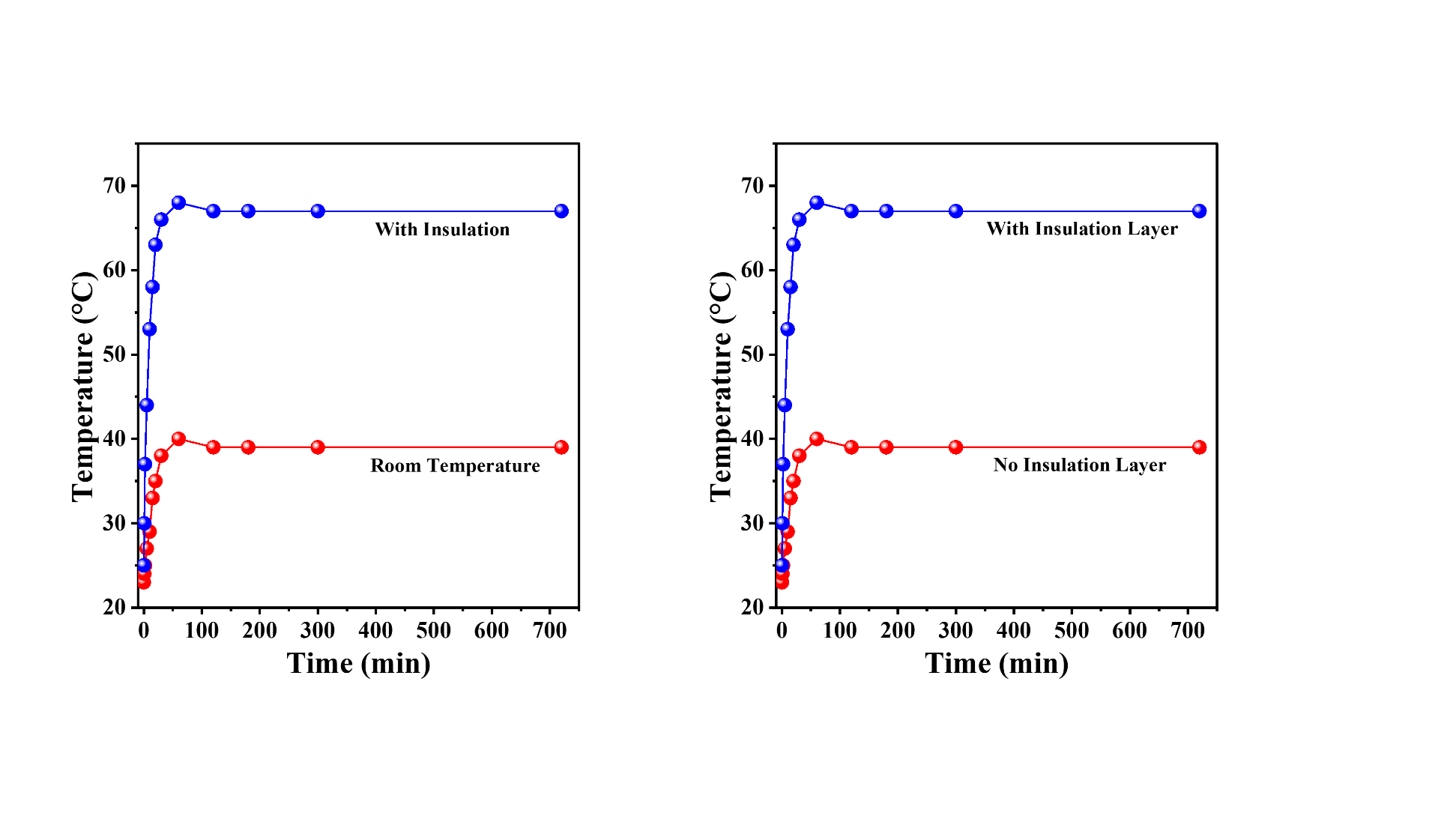


**Figure S11.** Reaction temperature curves for with and without insulation layer conditions, respectively.


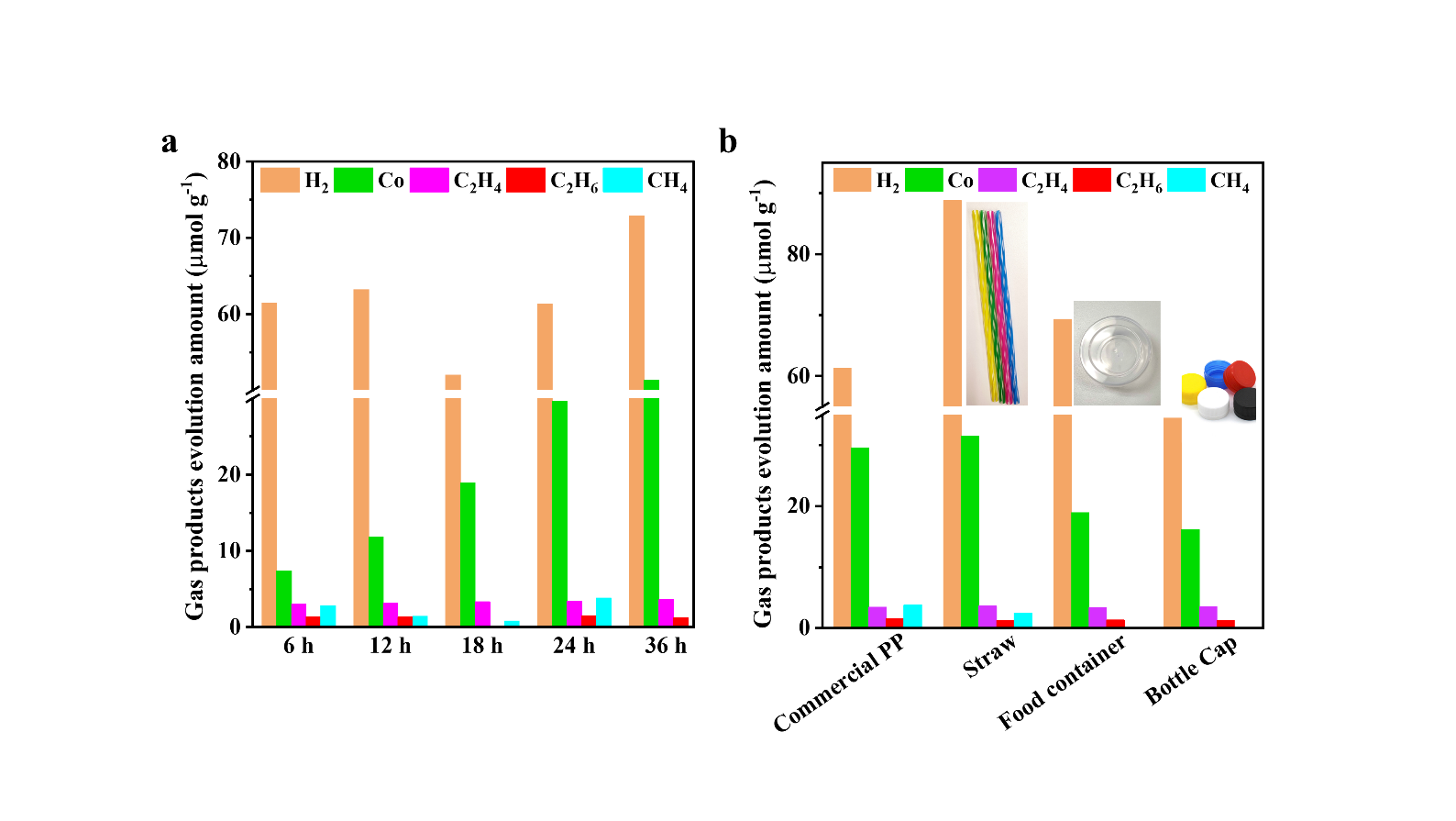


**Figure S12.** The produced gas products (a) over R1.00 in a 36-h reaction and (b) over R1.00 for photocatalytic conversion of real-world PP-based plastic wastes in the forms of straw, food containers, and bottle caps, respectively.

*
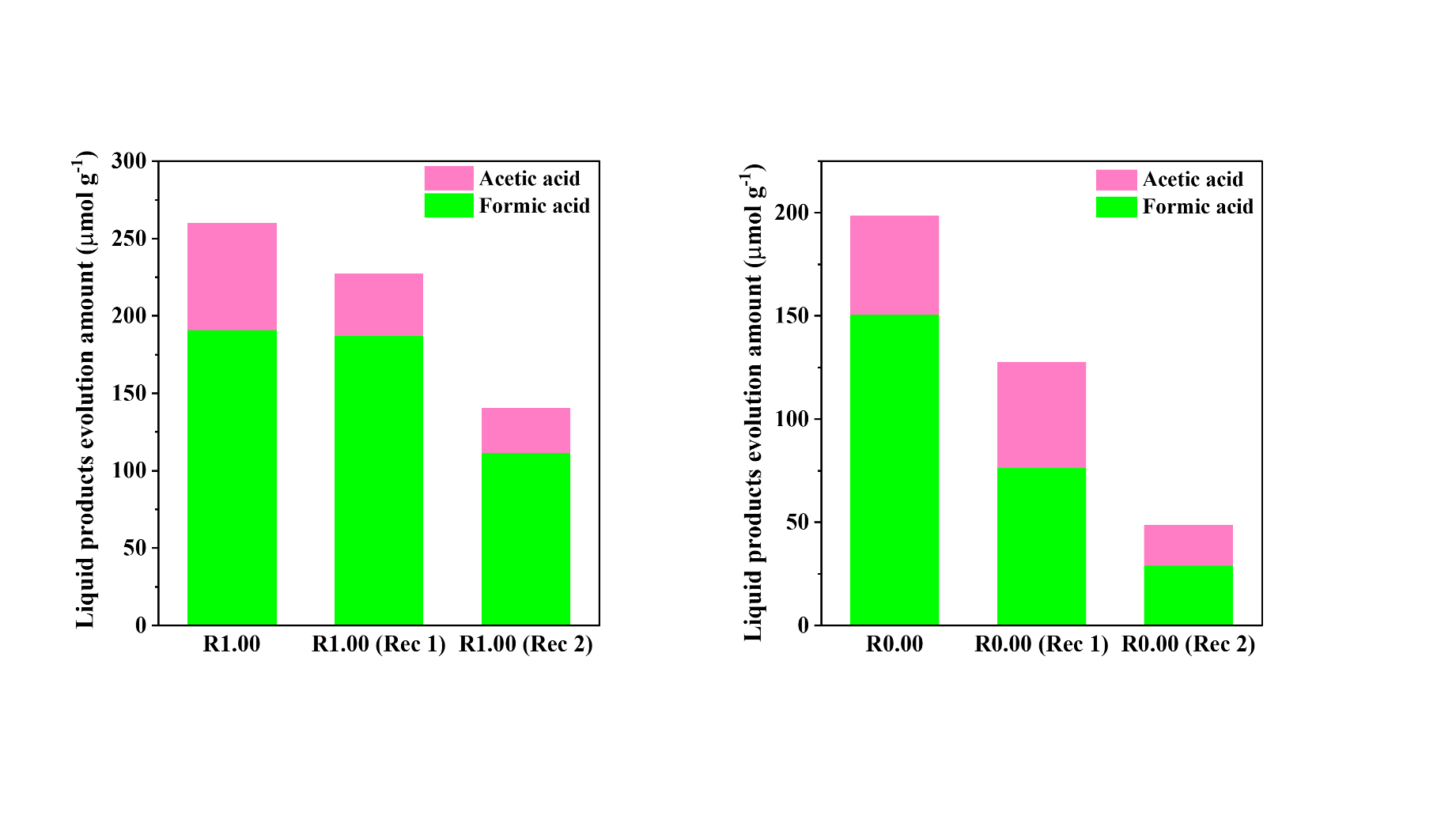
*

**Figure S13.** The recyclability of R1.00 throughout three consecutive 12 h tests in the presence of air and seawater.


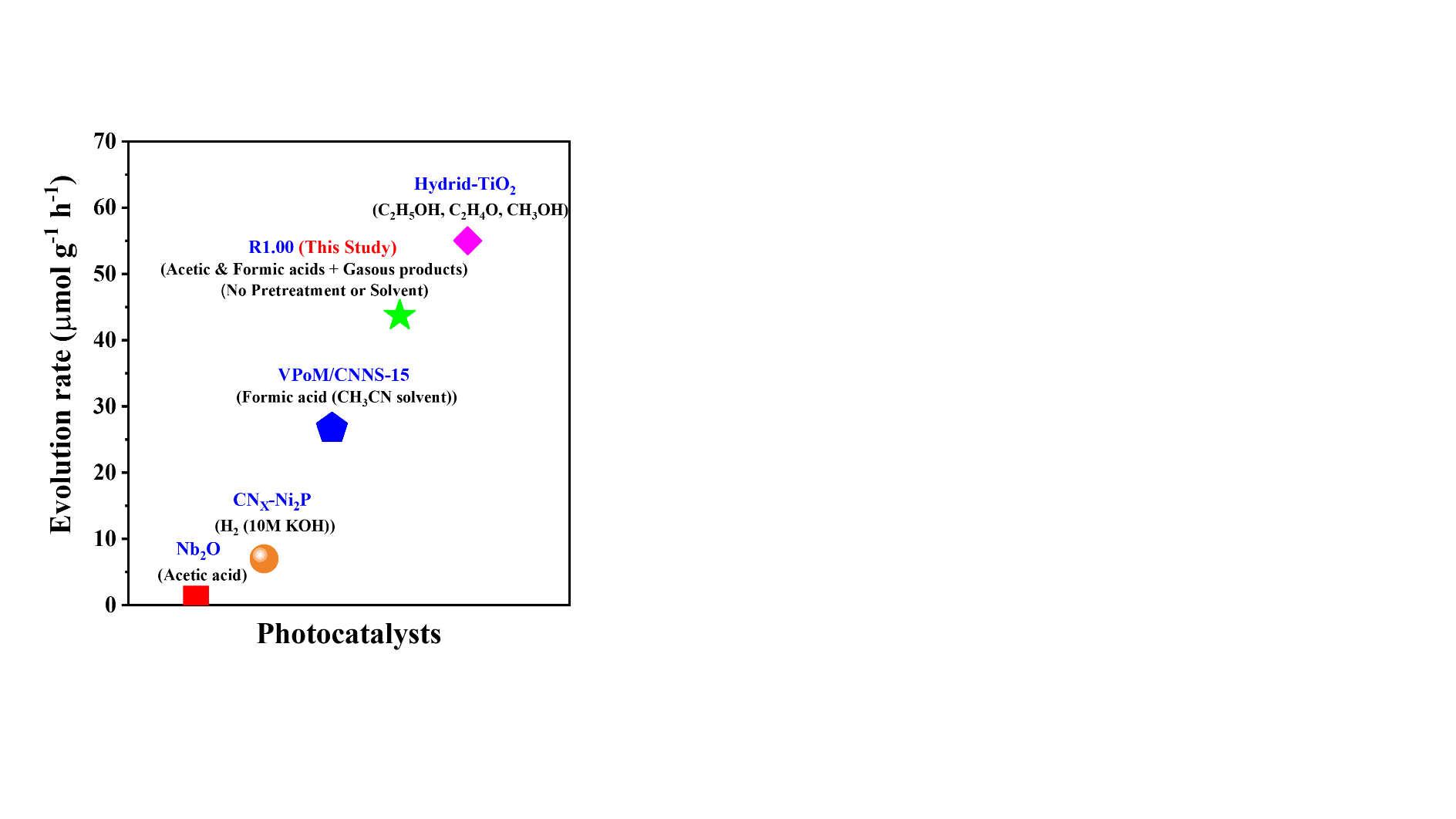


**Figure S14.** The acquired liquid product amounts in our research and other references reporting photocatalytic PP conversion.


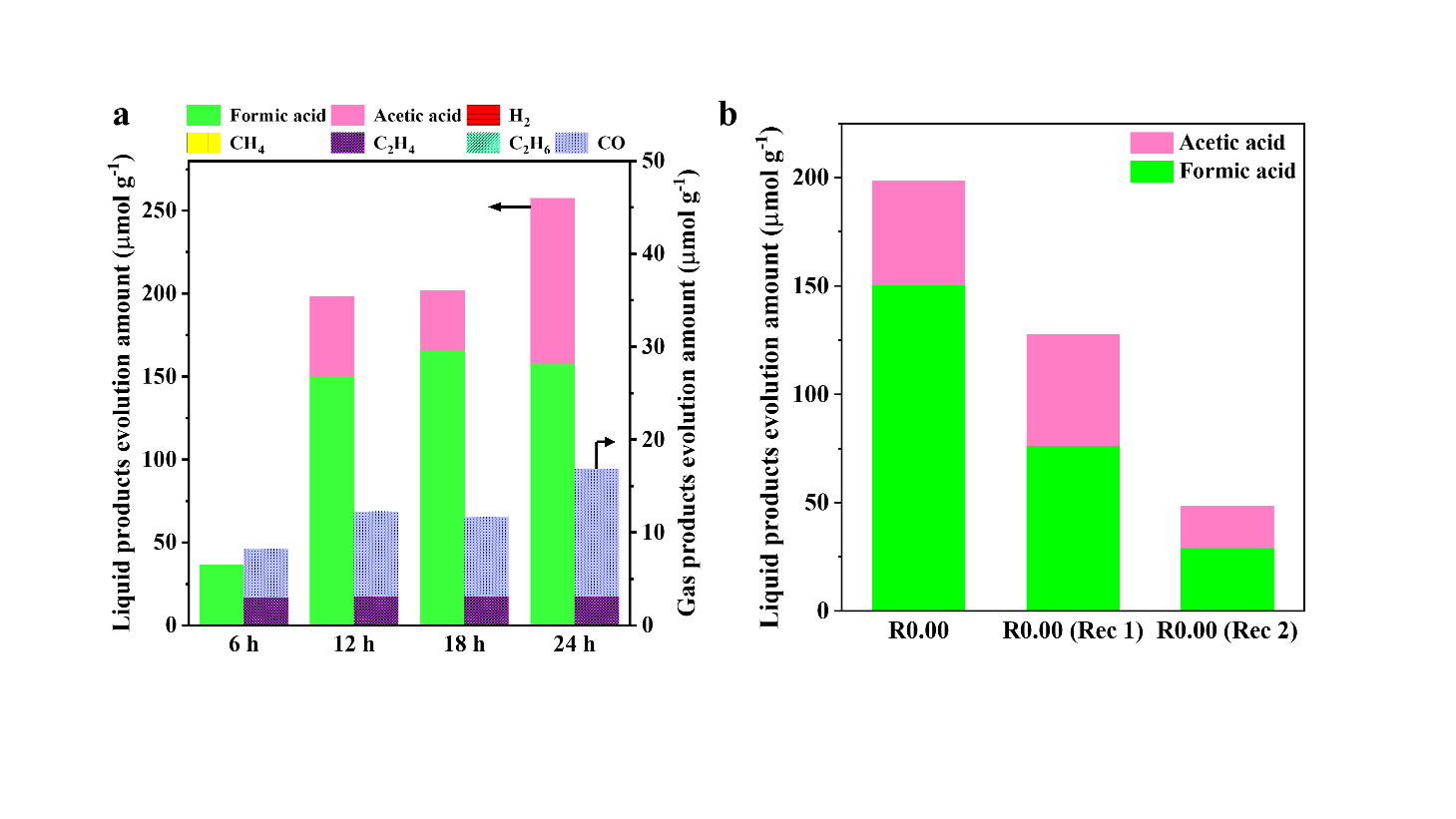


**Figure S15.** (a) Photocatalytic reforming of raw PP utilizing R0.00 for 24 h reaction in seawater, with xenon light irradiation and in air and (b) recyclability of R0.00 throughout three consecutive 12 h tests in seawater.


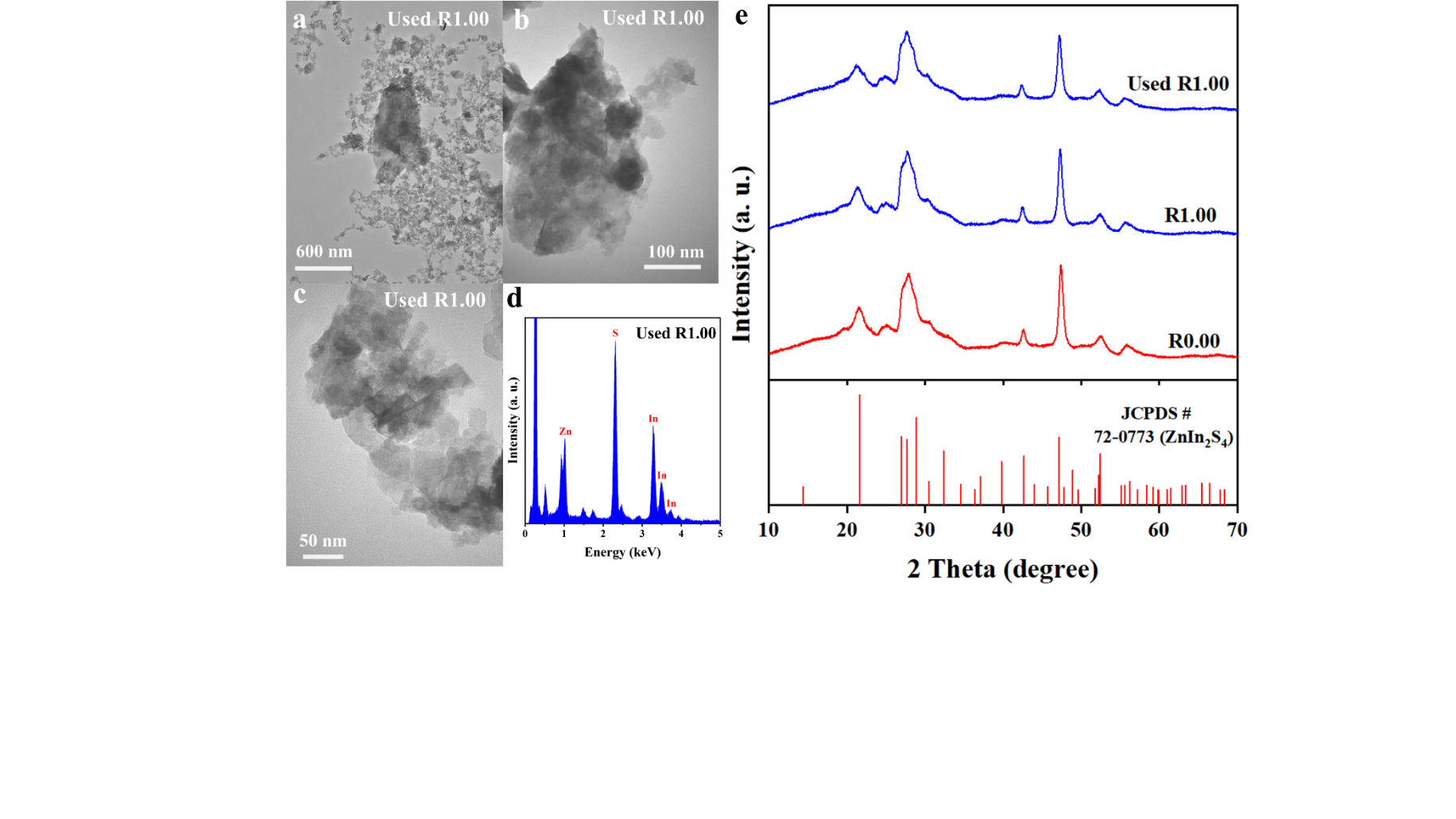


**Figure S16.** (a-c) TEM images and (d) EDX spectrum of used R1.00. (e) XRD patterns of R0.00, R1.00 and used R1.00.


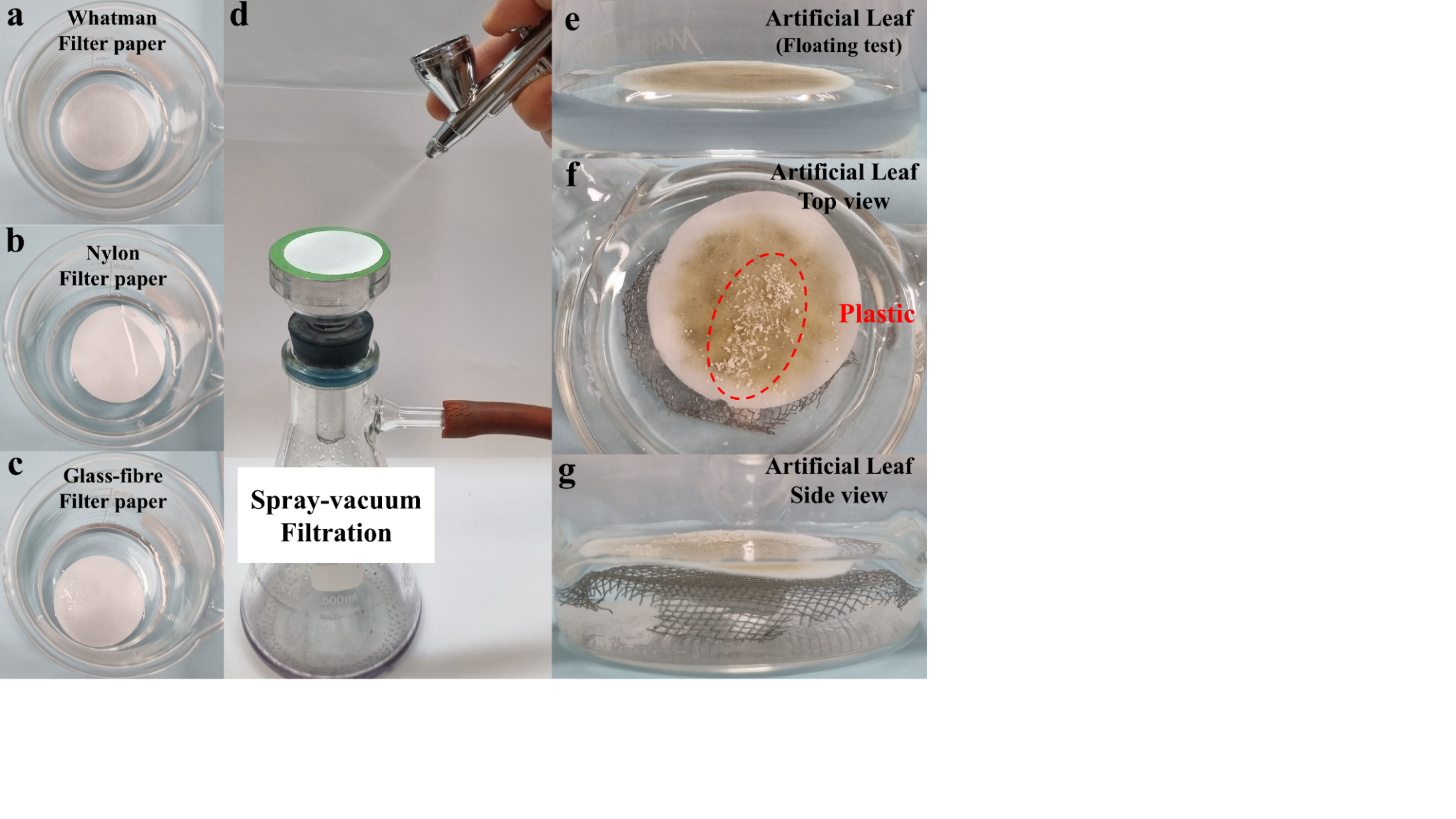


**Figure S17.** (a-c) The floatability test of different filter papers. (d) The preparation of the floatable artificial leaf (AL) photocatalyst by the spray-vacuum Route. (e) The AL floatability test, and (f-g) The AL in test conditions from top and side views.


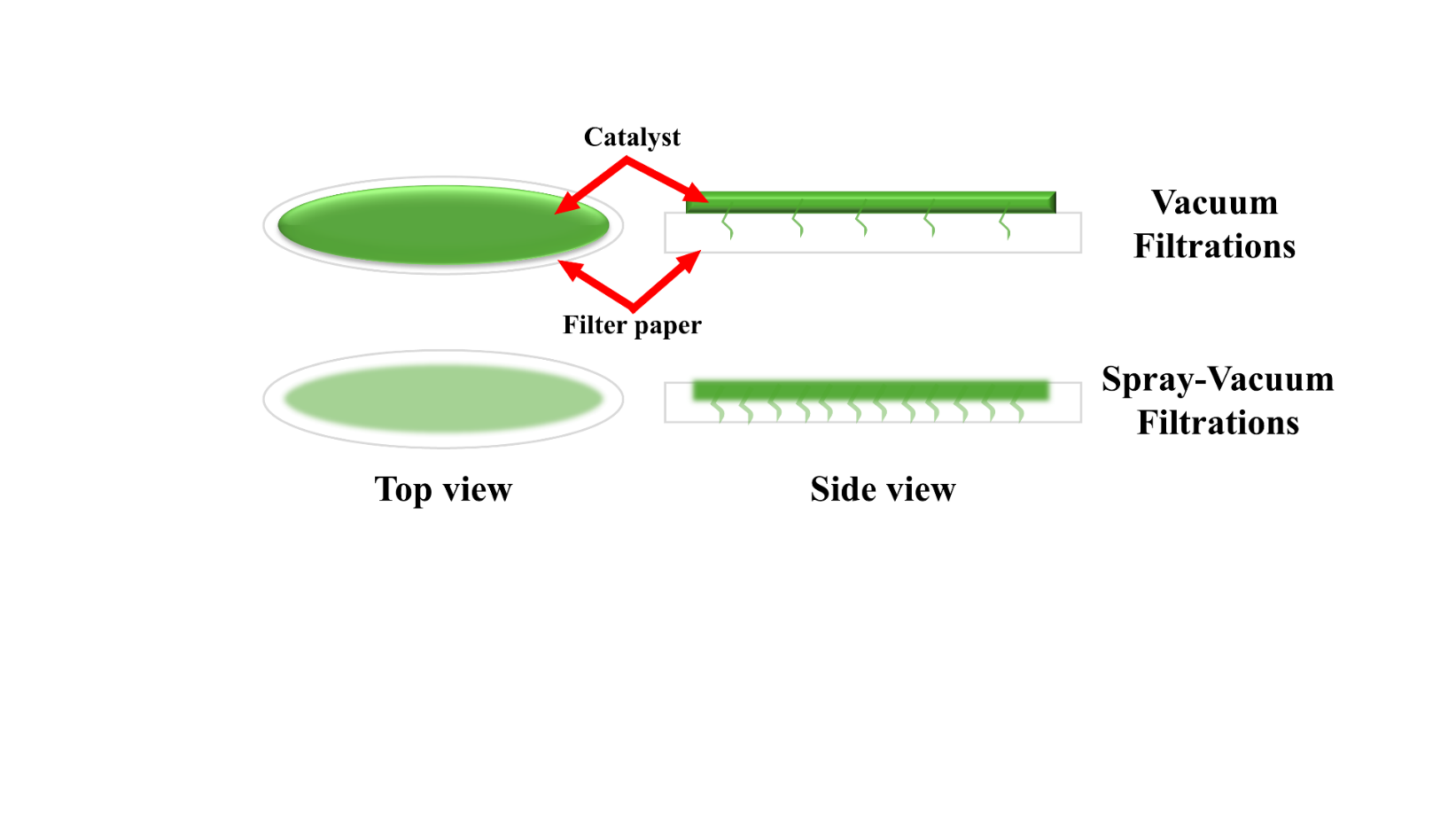


**Figure S18.** The comparison between regular vacuum-filtration and spray-vacuum filtration approaches.


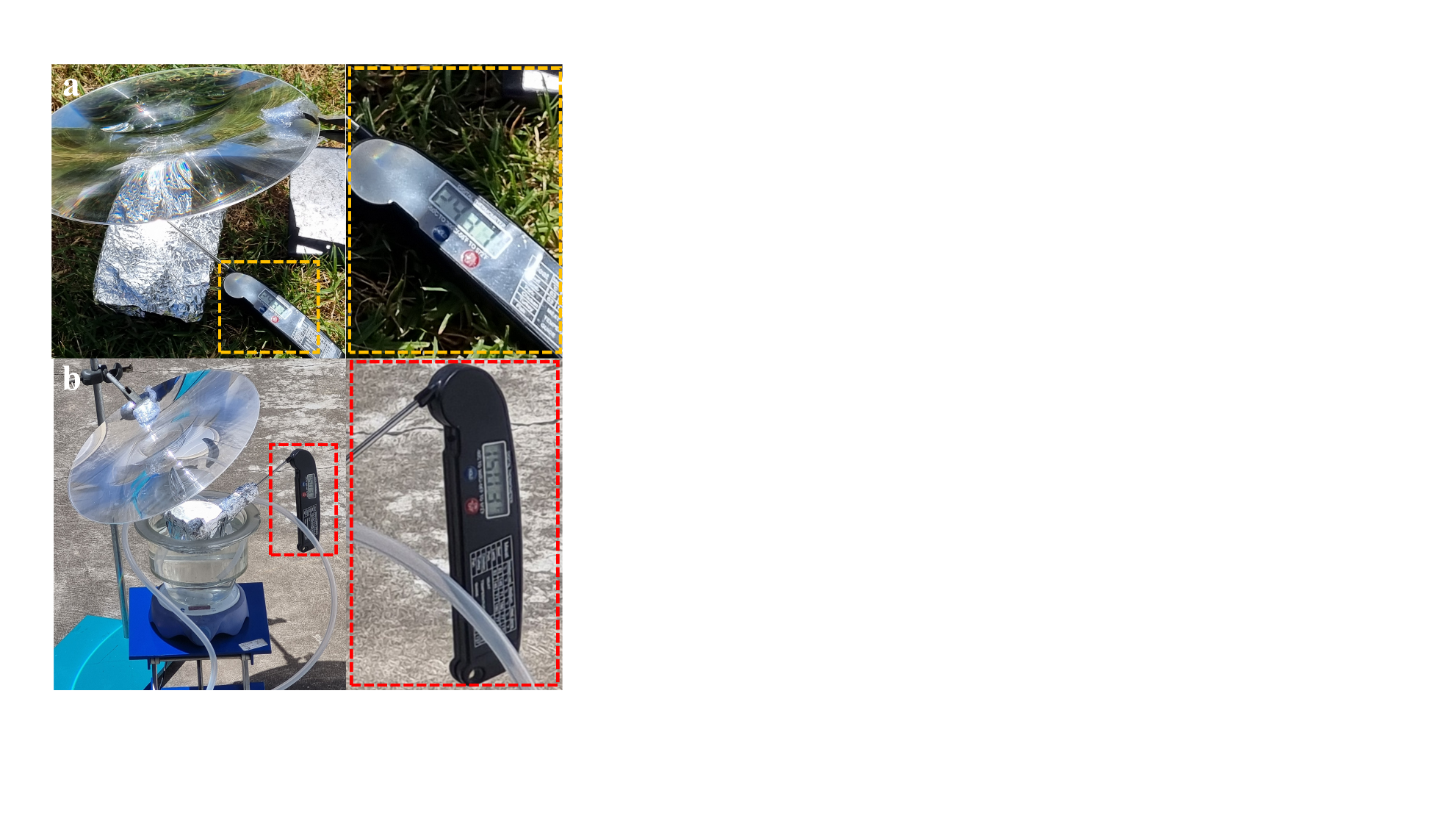


**Figure S19.** The effect of solar light concentrator on reaction temperature.


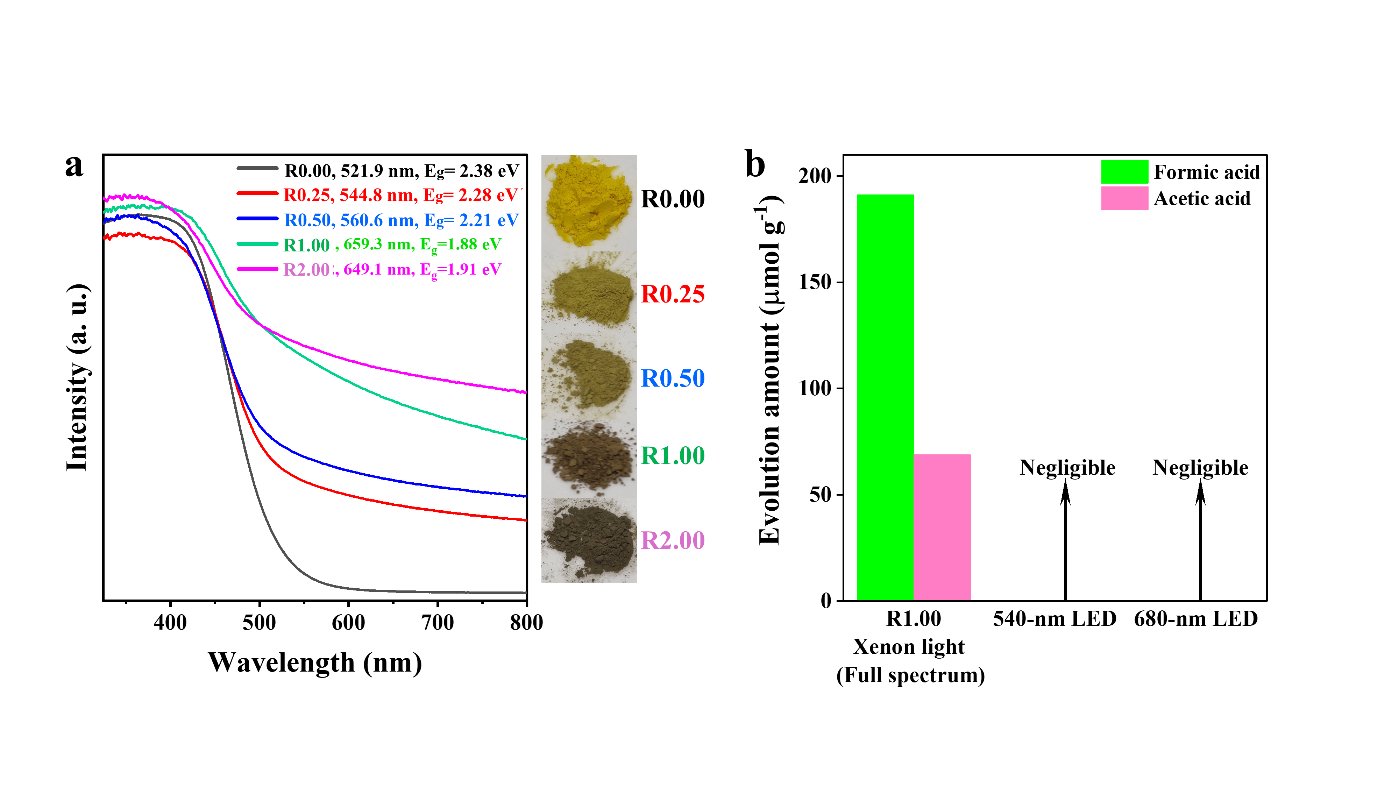


**Figure S20.** (a) UV-vis diffuse reflectance spectra of R0.00, R0.25, R0.50, R1.00, and R2.00 and (b) Photocatalytic PP conversion performances of R1.00 with xenon light irradiation (full spectrum), 540-nm LED irradiation, and 680-nm LED irradiation, respectively.


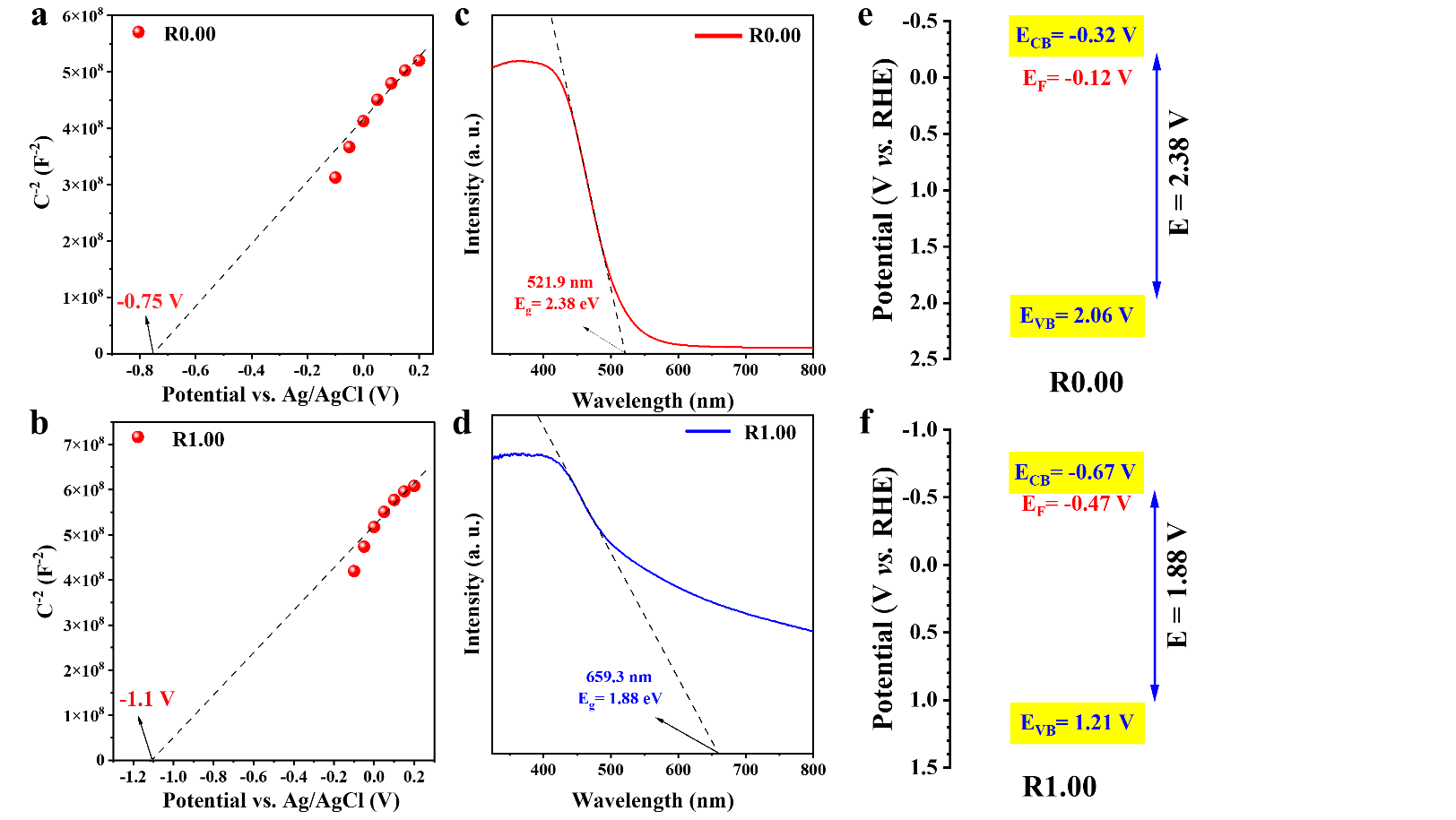


**Figure S21.** Mott-Schottky plots for (a) R0.00 and (b) R1.00. UV-vis diffuse reflectance spectra for (c) R0.00 and (d) R1.00. The CB and VB band positions of (e) R0.00 and (f) R1.00.


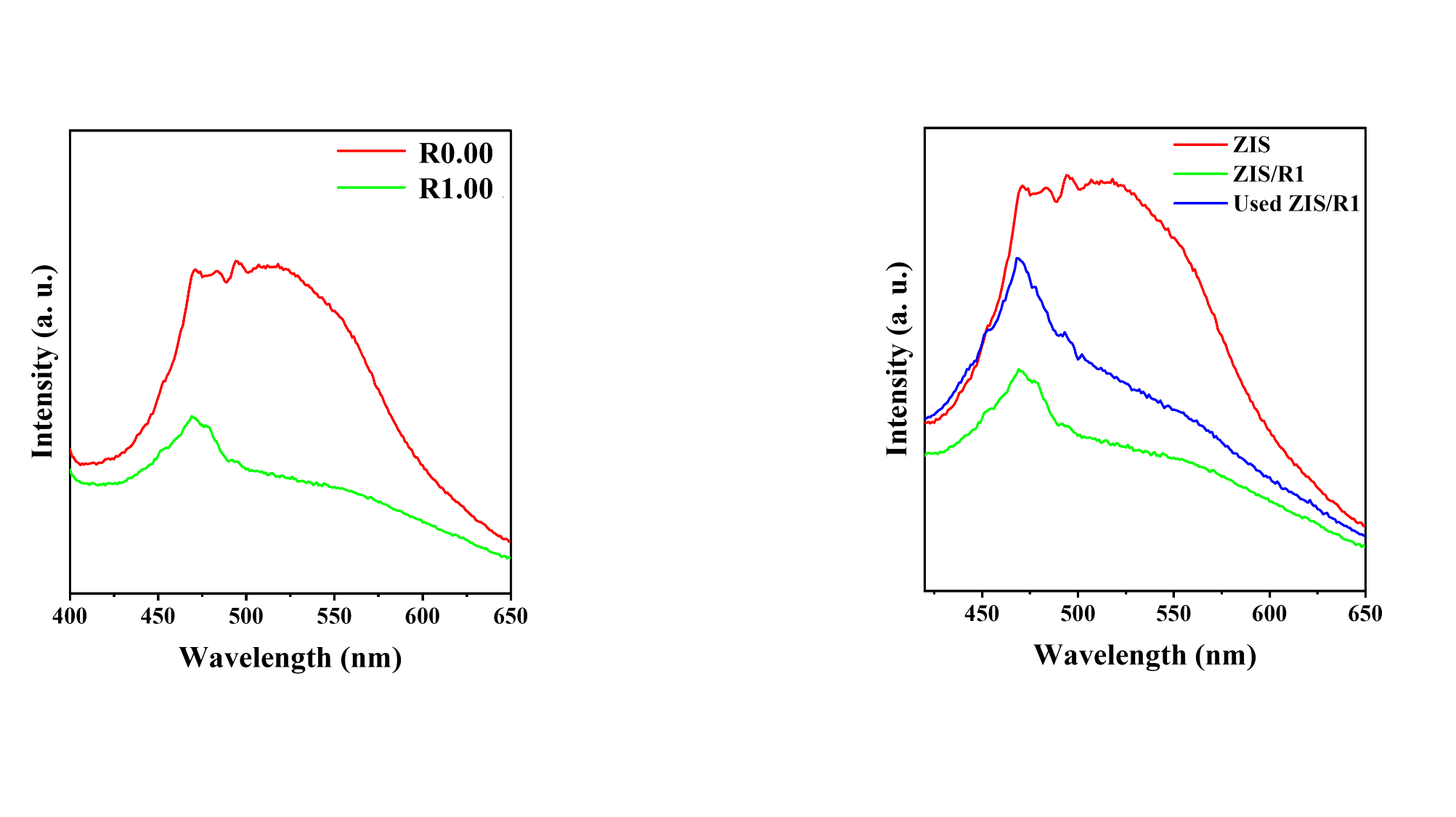


**Figure S22.** The steady-state PL spectra of R0.00 and R1.00.


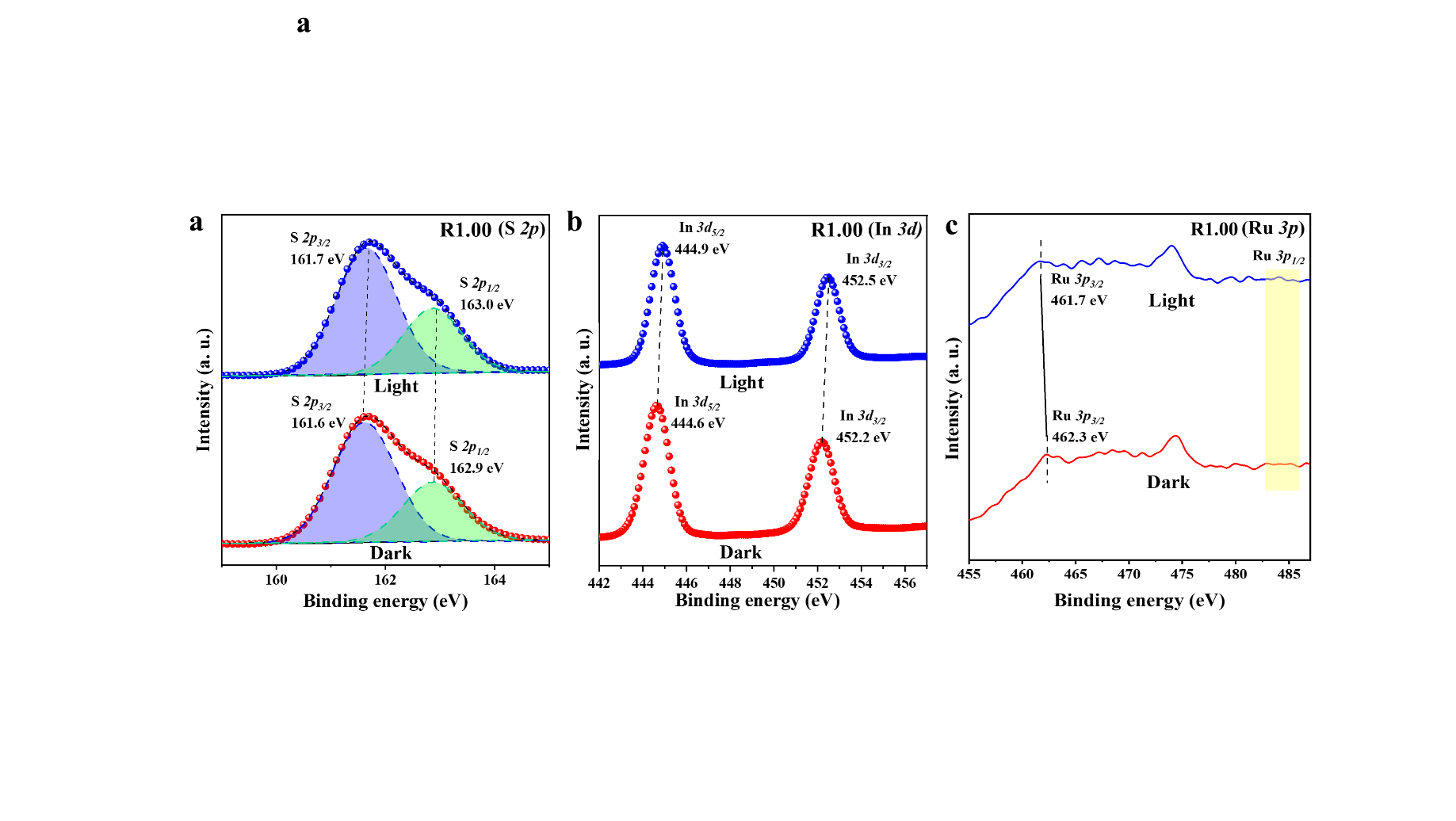


**Figure S23.** High-Resolution XPS spectra of (a) S 2p, (b) In 3d, and (c) Ru 3p for R1.00 in dark and under light irradiation.


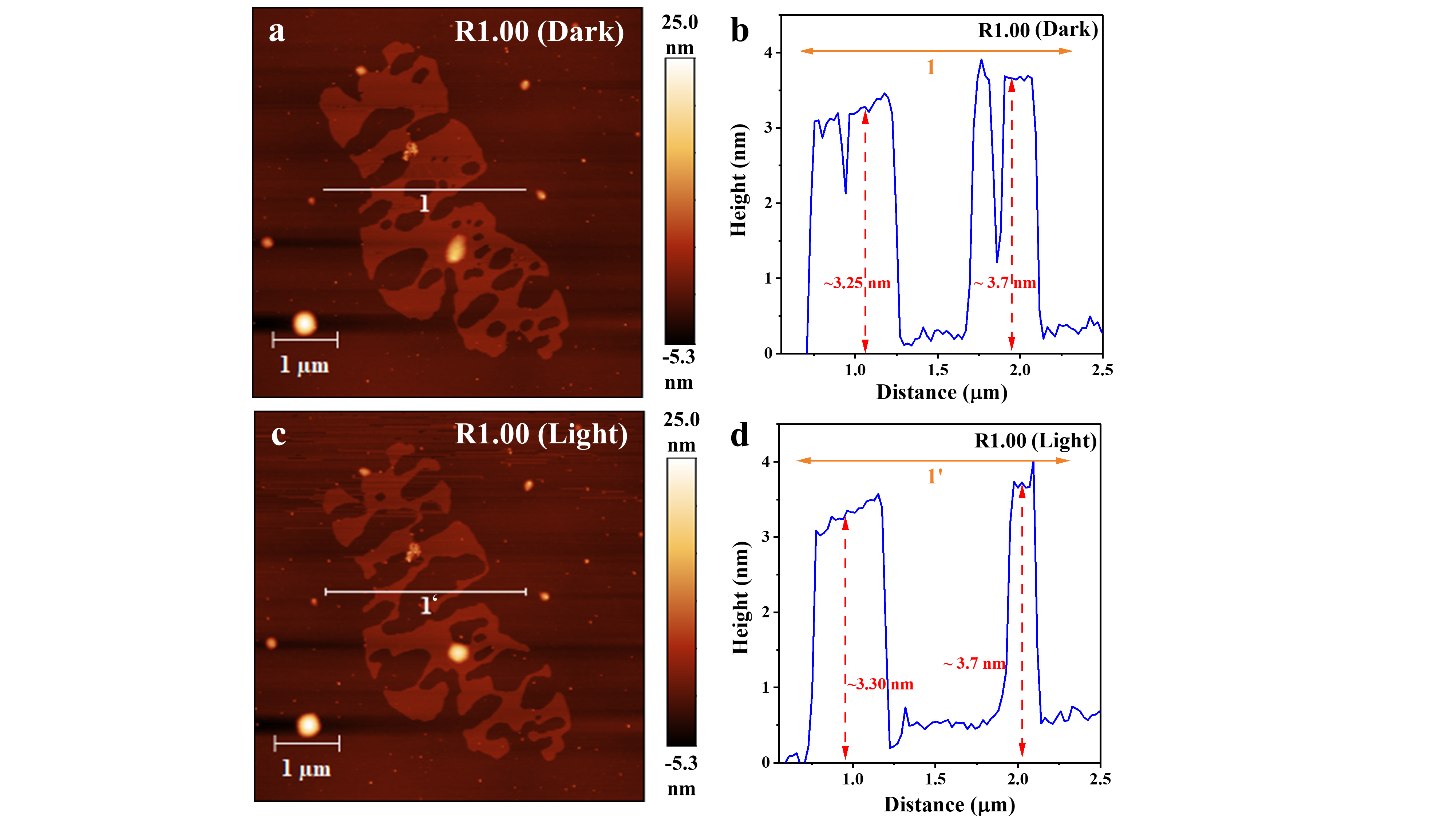


Figure S24. (a) AFM image and (b) corresponding height profile of R1.00 without light irradiation. (c) AFM image and (d) corresponding height profile of R1.00 with light irradiation.

**
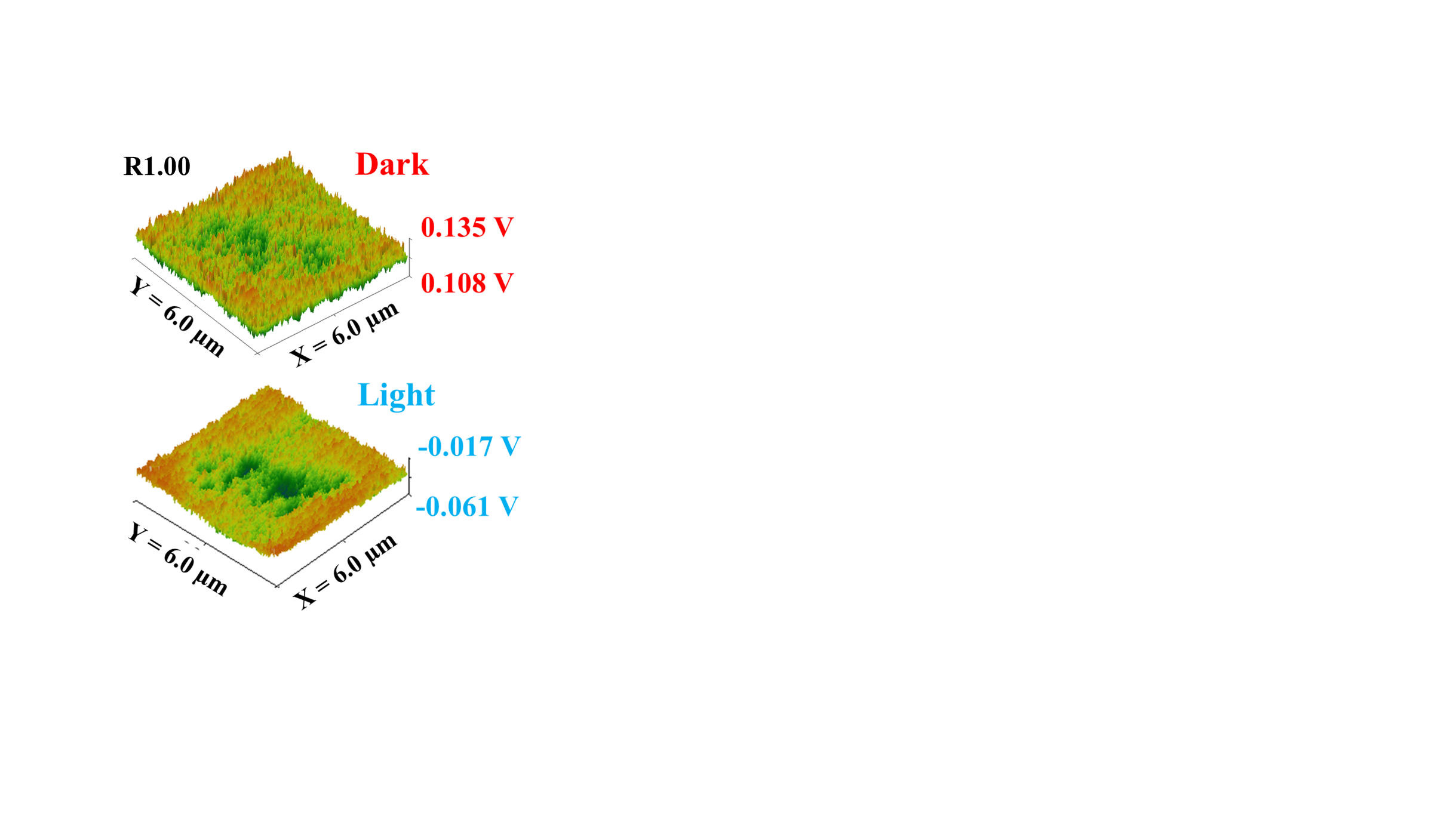
**

**Figure S25.** 3D images of corresponding potential signals for R1.00 in dark and under light irradiation.


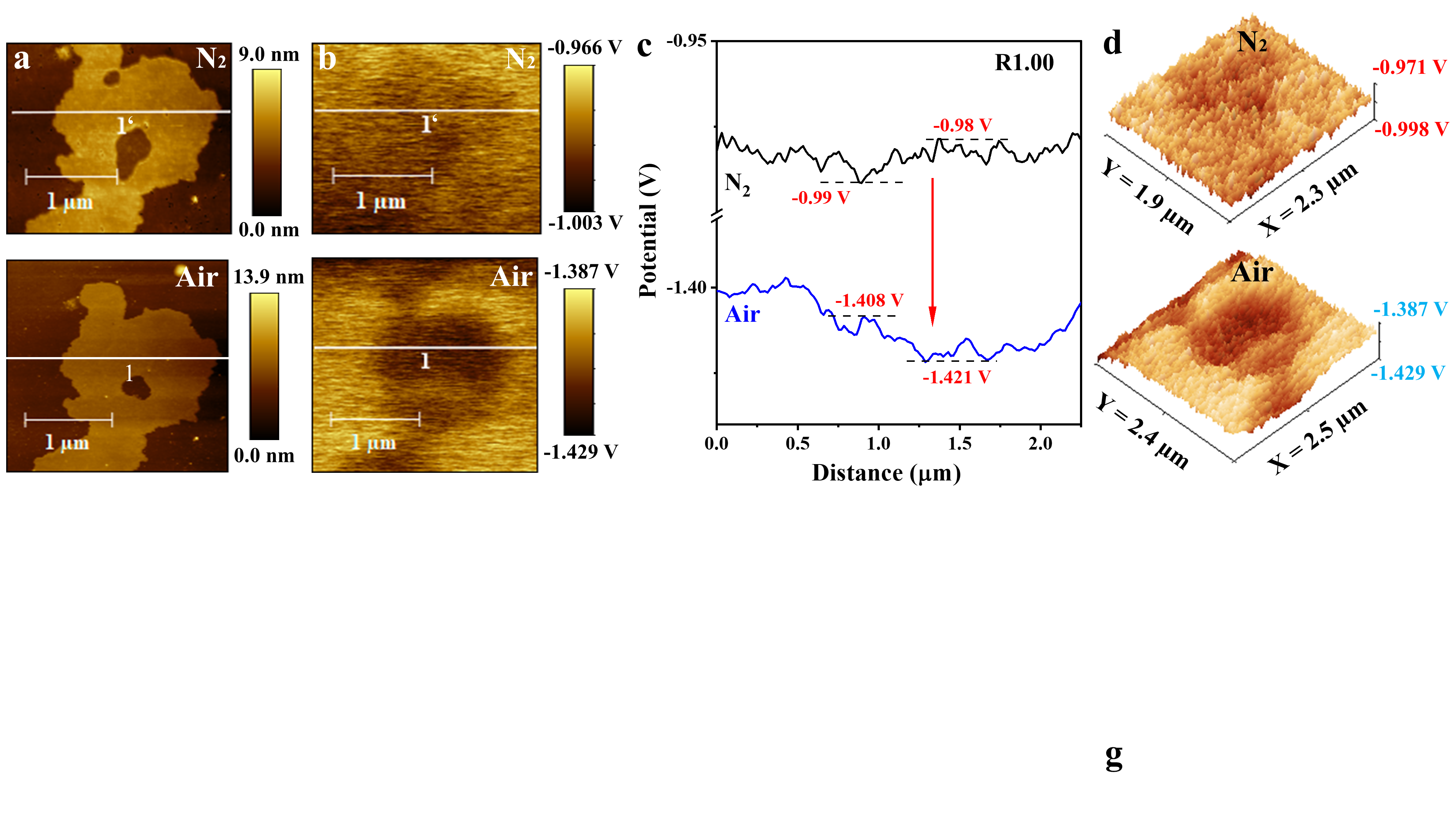


**Figure S26.** (a) AFM images and (b) KPFM images of R1.00 in N_2_ and air atmospheres, respectively. (c) The corresponding line analysis of potential signals for R1.00 in N_2_ and air atmospheres, respectively, along line 1 and 1’. (d) 3D image of potential signals for R1.00 in N_2_ and air atmospheres, respectively.


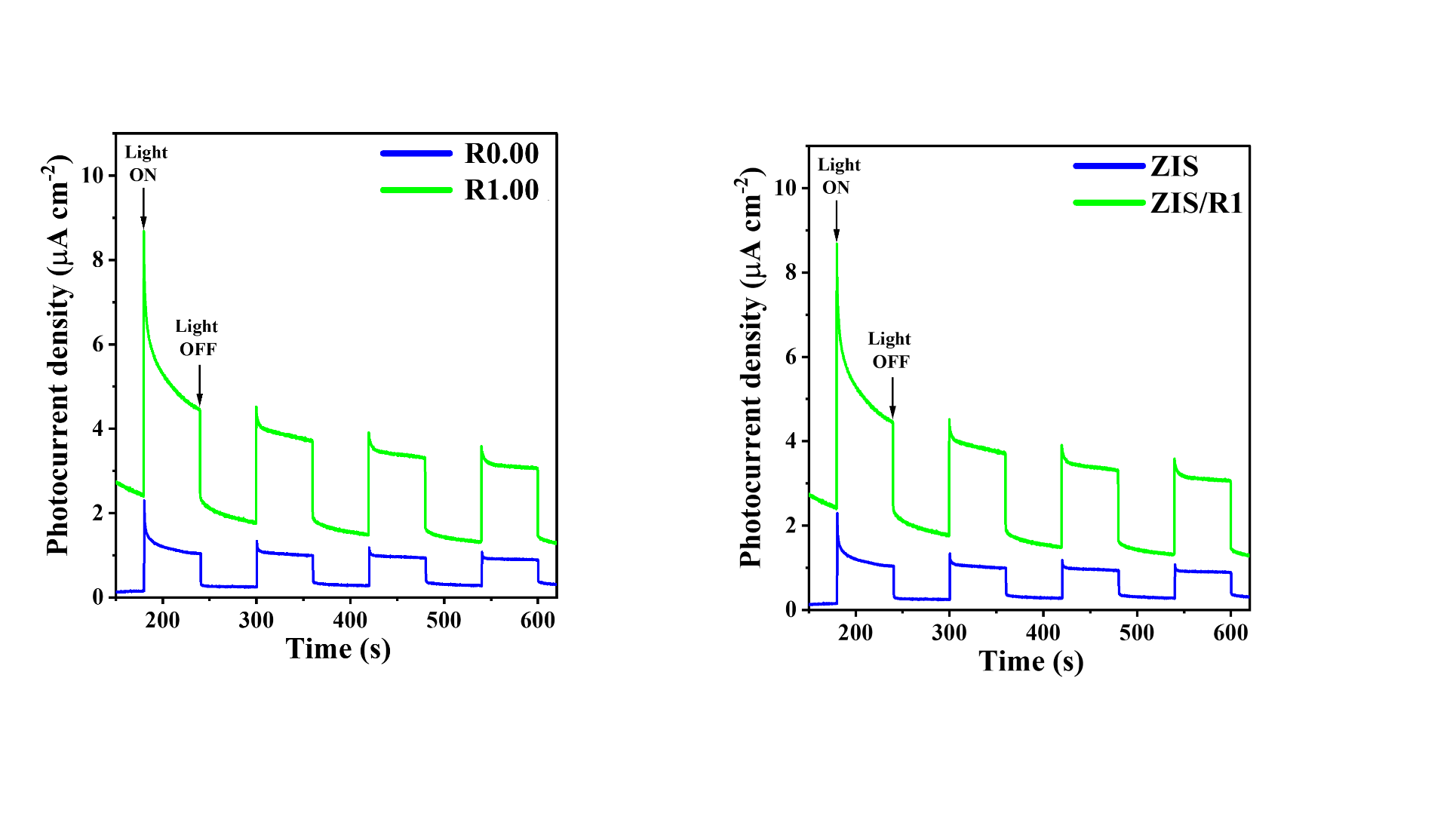


**Figure S27.** TPC density measurements of R0.00 and R1.00 in 0.5 M Na_2_SO_4_ aqueous solution.


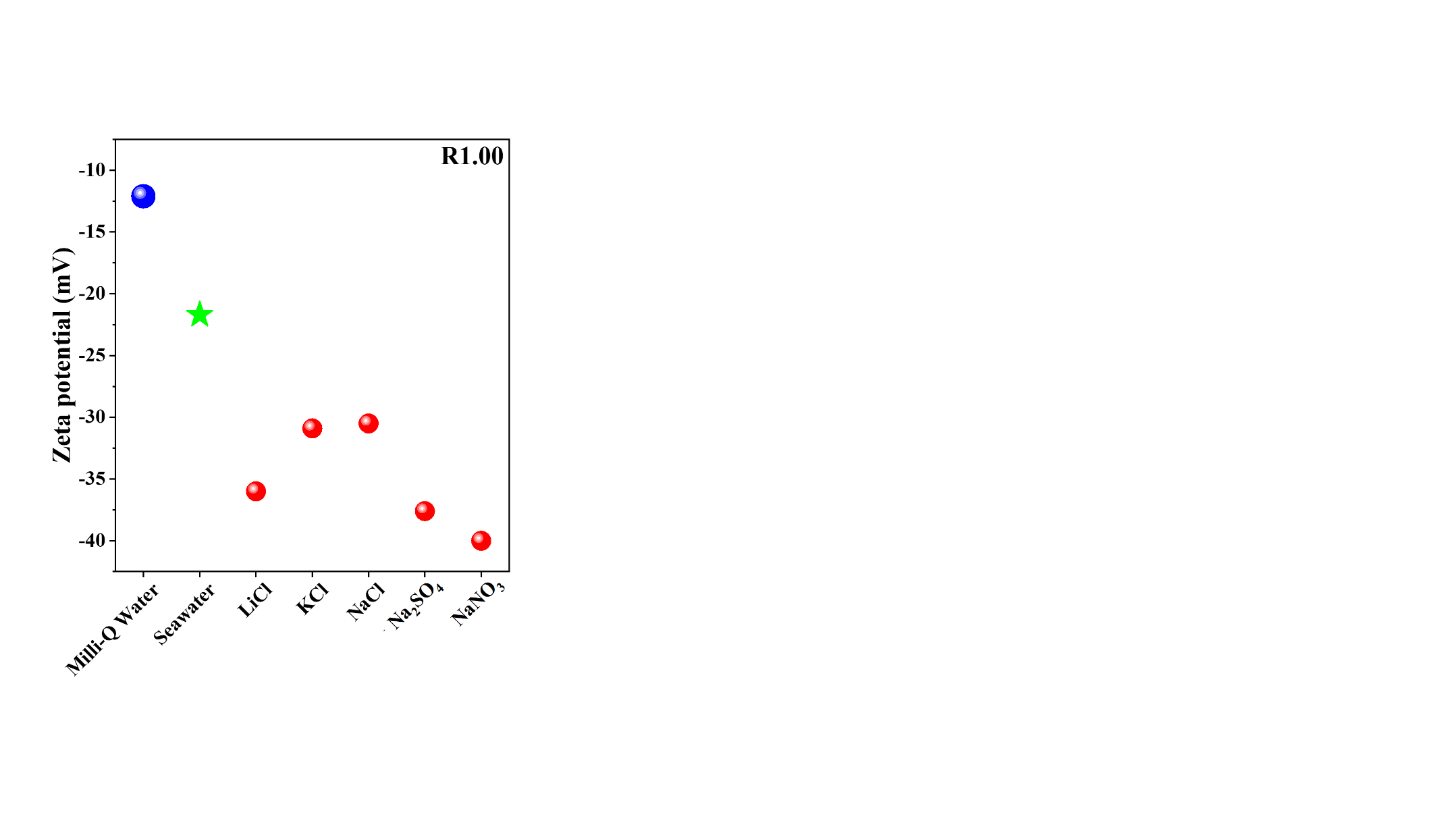


**Figure S28.** The impact of various seawater ions on zeta potential.


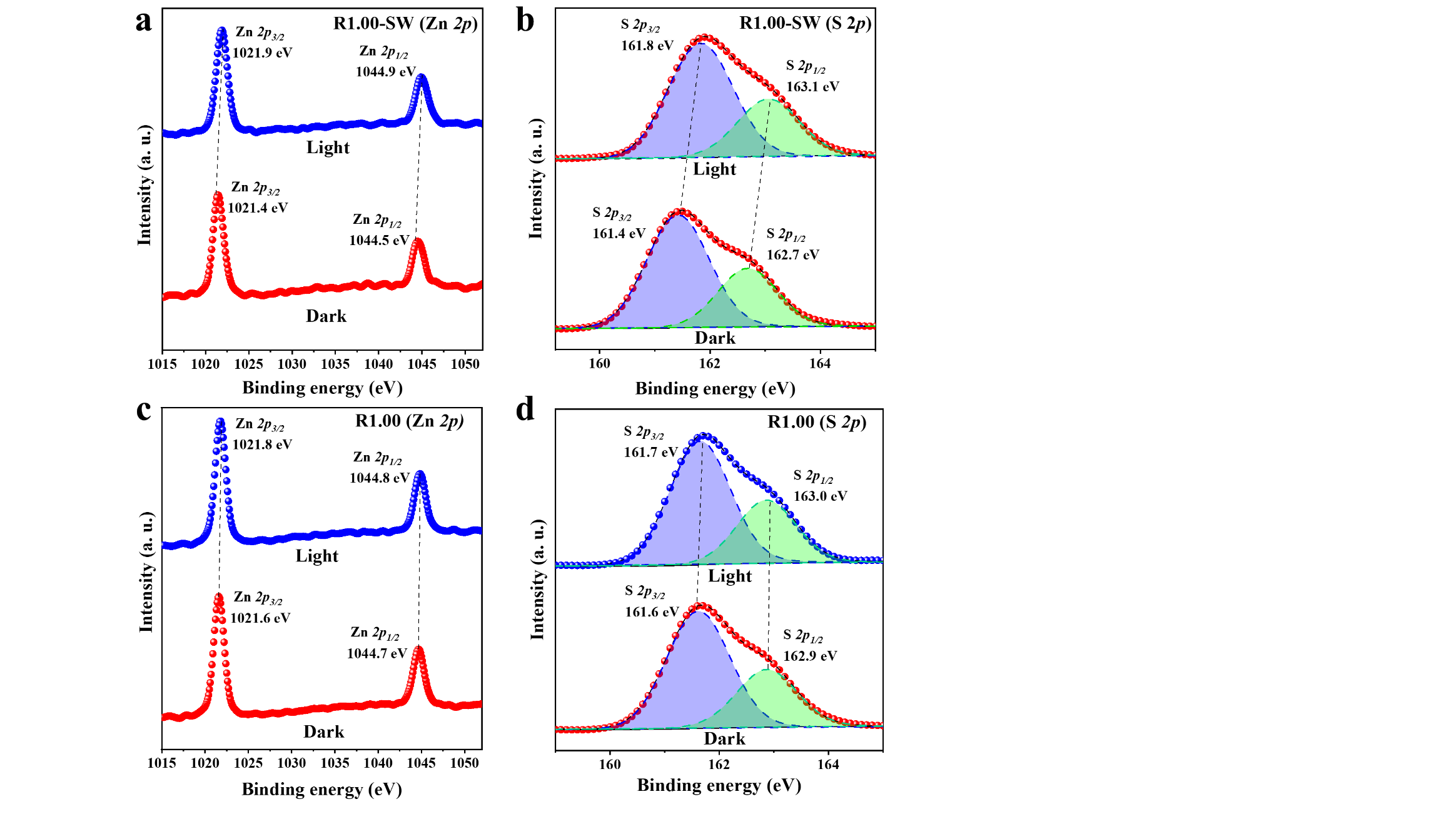


**Figure S29.** High-Resolution XPS spectra of (a) Zn 2p, (b) S 2p for R1.00-SW and (c) Zn 2p, and (d) S 2p for R1.00 in dark and under light irradiation.


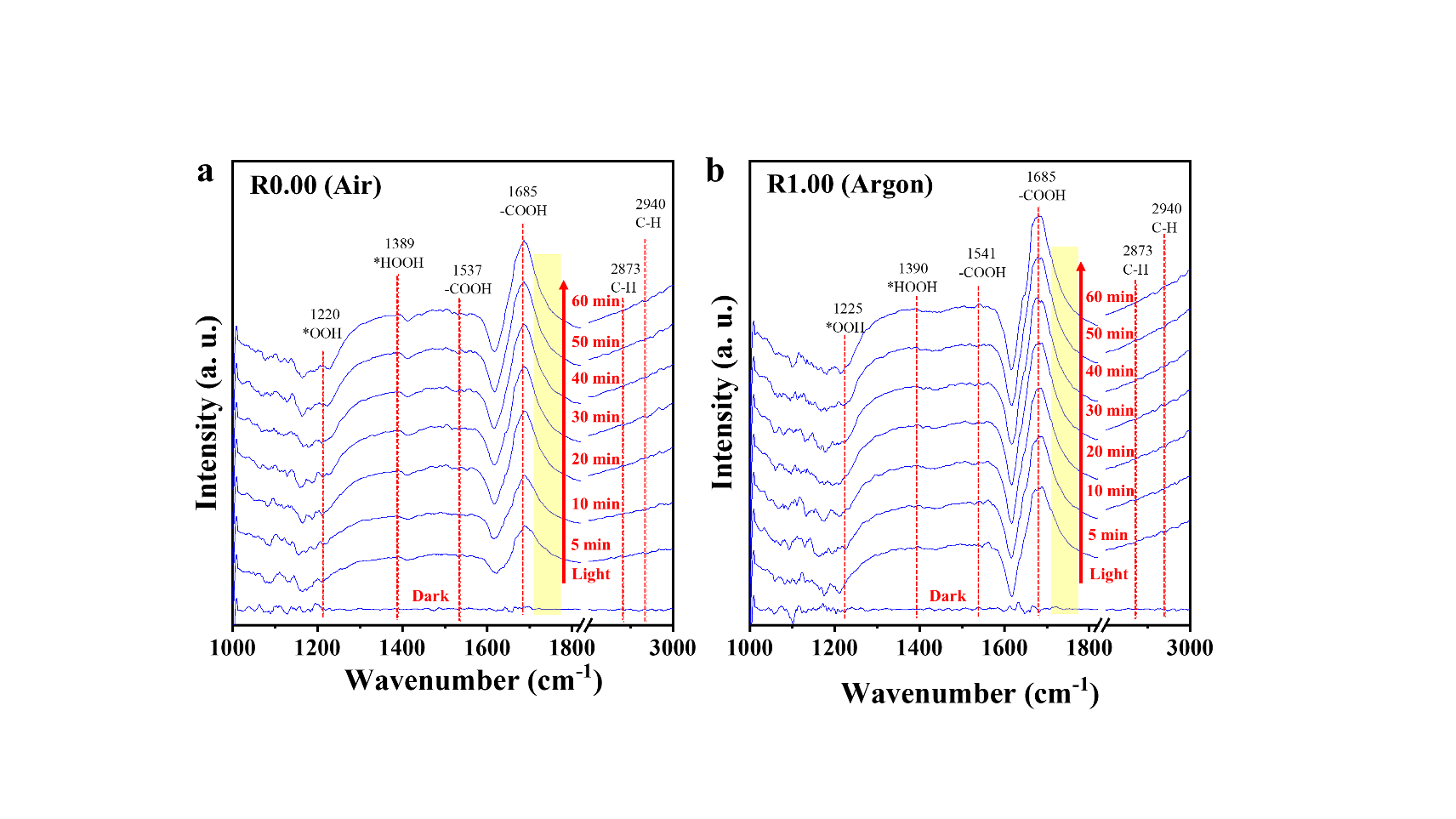


**Figure S30.** In situ IR spectra of photocatalytic reforming PP over (a) R0.0 in air and (b) R1.00 in argon.


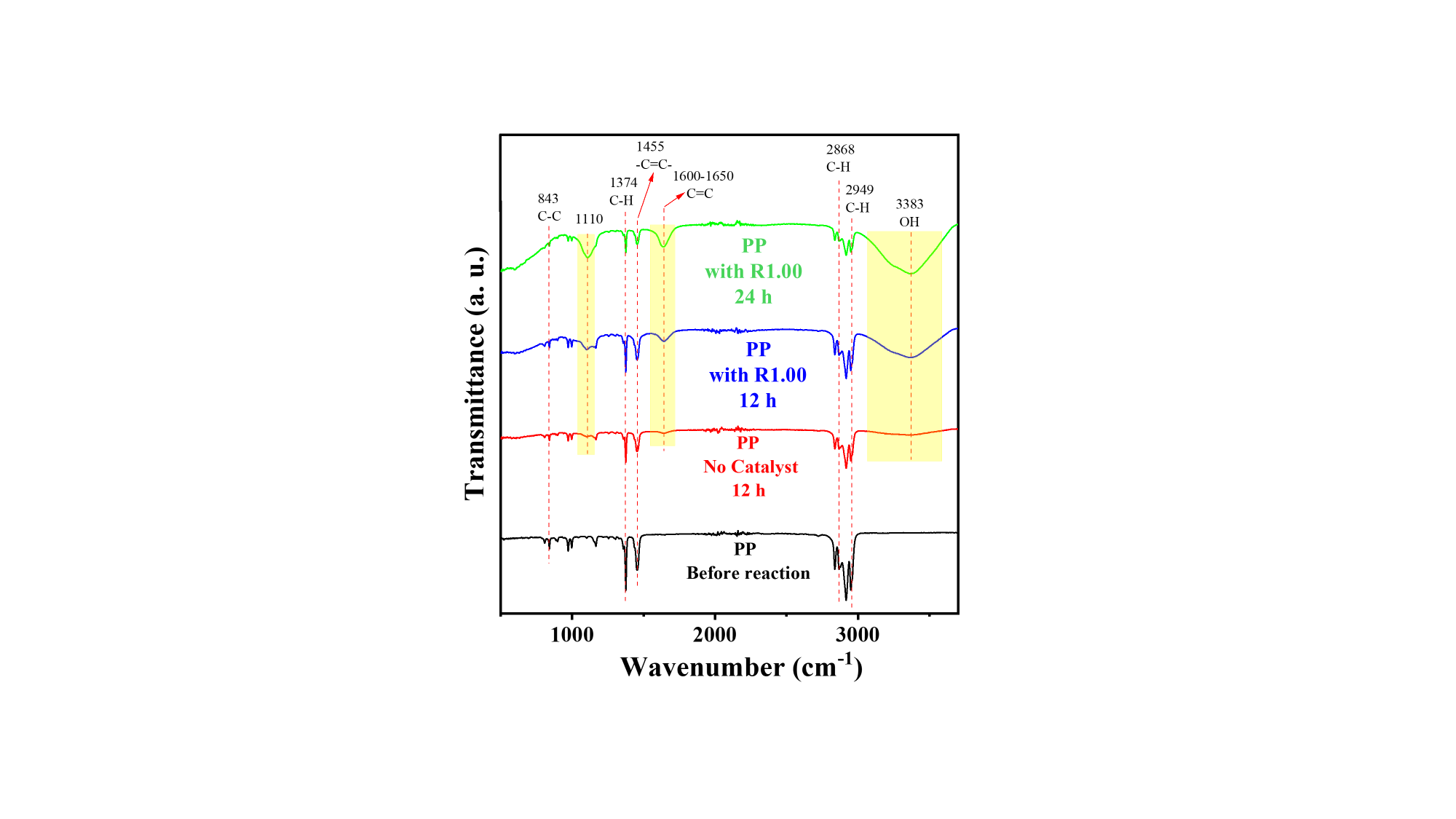


**Figure S31.** IR spectra of PP plastic before reaction, Reacted PP plastic with no catalyst after 12 h reaction, Reacted PP plastic with R1.00 after 12 h reaction and Reacted PP plastic with R1.00 after 24 h reaction.


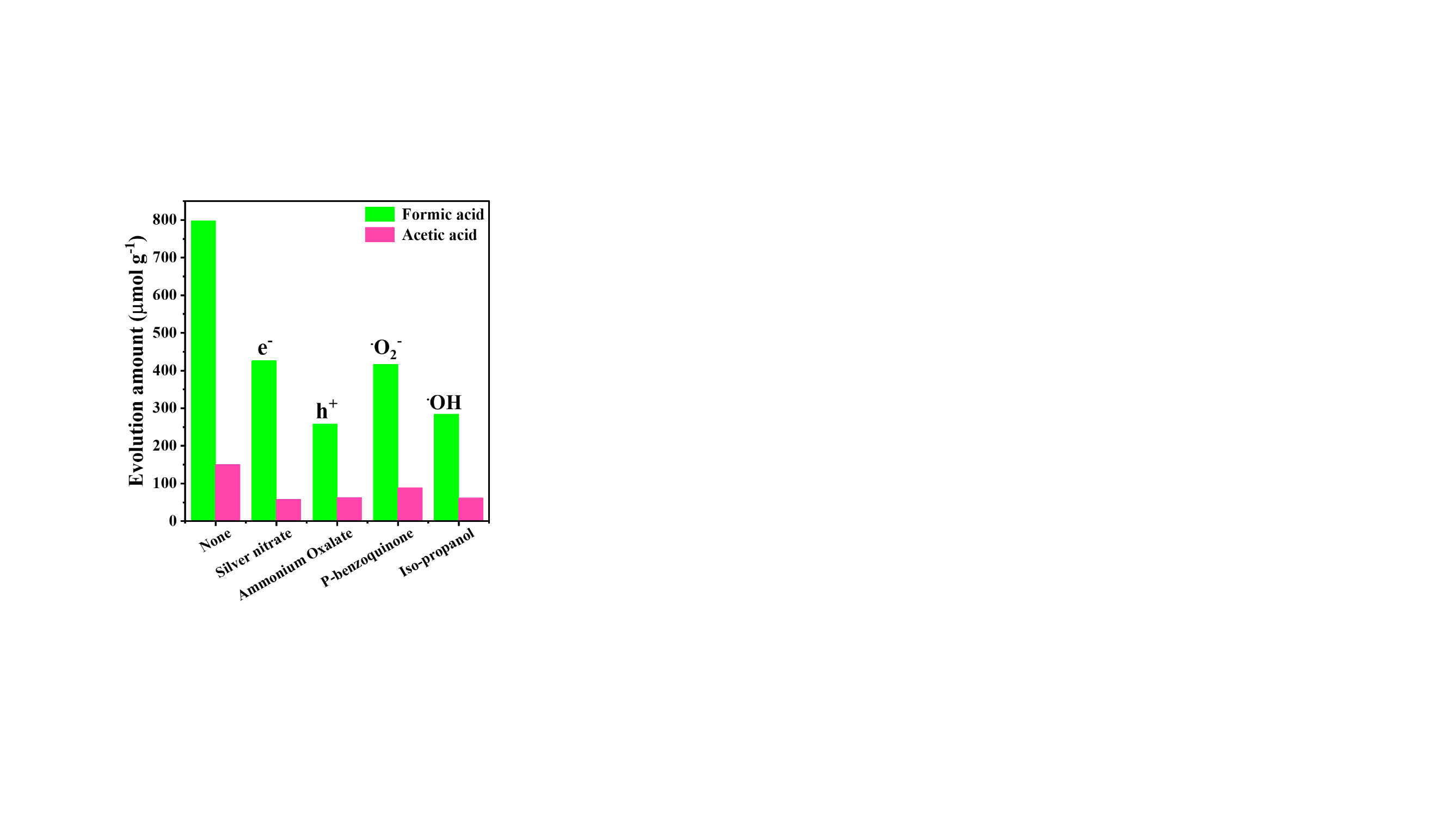


**Figure S32.** The liquid products (formic acid and acetic acid) production amounts over R1.00 after adding silver nitrate, ammonium oxalate, p-benzoquinone, and iso-propanol, as the scavenger of e^−^, h^+^, ∙O_2_^−^, and ∙OH, respectively.


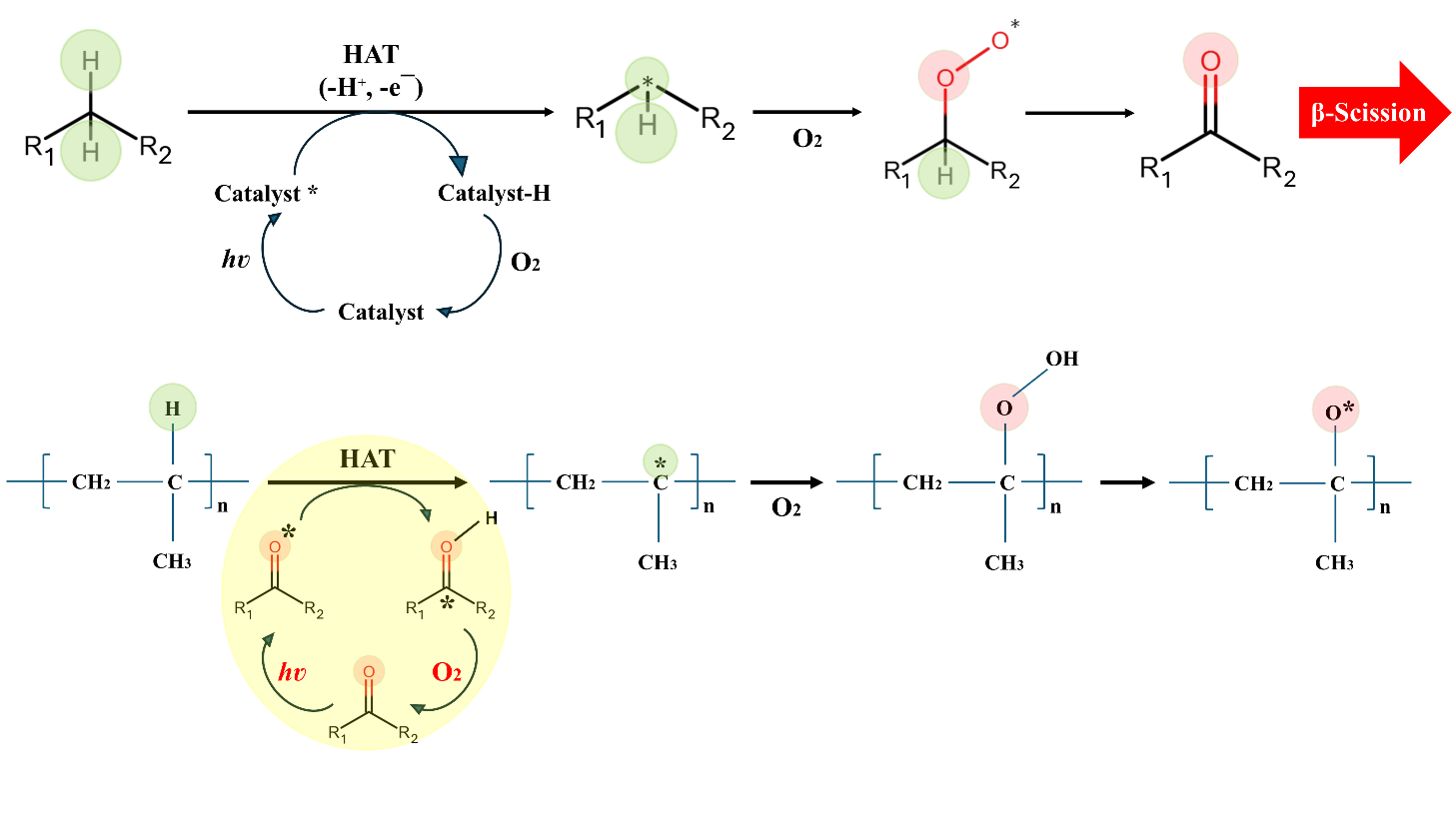


**Figure S33.** C-H oxidation process of polymers by HAT phenomenon.

**
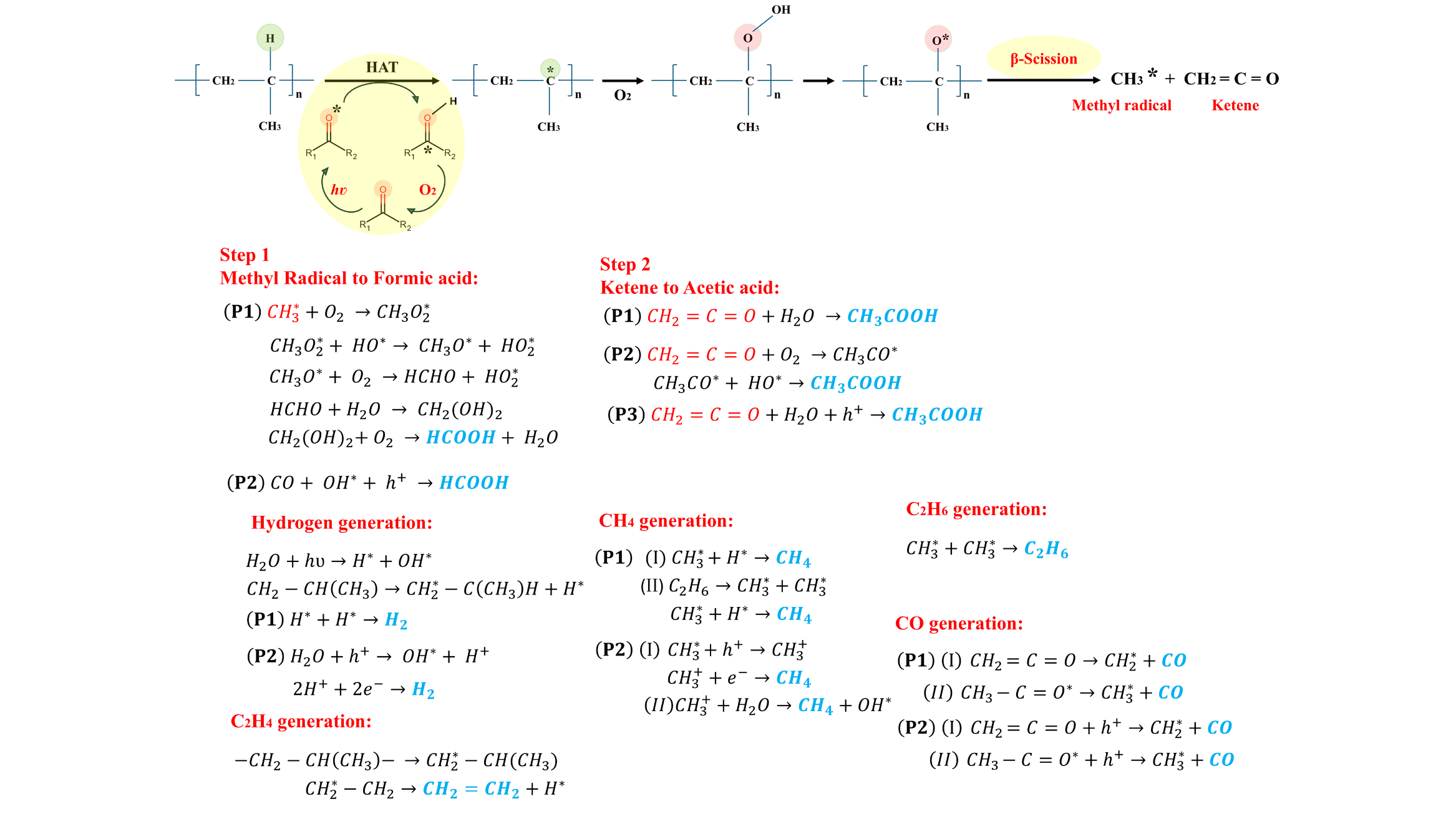
**

**Figure S34.** Raised photocatalytic reaction pathways for PP upcycling.

**Table S1**. A comparison of photocatalytic PP and PE plastic upcycling in the reported references and this research.

| **Photocatalysts**  **(mg)** | **Reaction conditions** | **Substrates** | **Value-added chemicals**  **(µmol g^-1^)** | **Ref** |
| --- | --- | --- | --- | --- |
| R1.00  (10 mg) | 300 W Xenon Light,  10 ml Seawater, Air, 36 h | 20 mg PP | Liquid products = 1022.5 µmol g^-1^  Gas products = 129.3 µmol g^-1^ | Our Research |
| Nb_2_O_5_  (50 mg) | 300 W Xenon Light, DI water,  Oxygen, 9 h | PP  (150 mg) | CO_2_= 16.3 mg g^-1^ h^-1^  Acetic acid = 39.6 µg g_cat_^-1^ h^-1^ | [2] |
|  |  | Polyethylene (PE)  (150 mg) | CO_2_= 25.5 mg g^-1^ h^-1^  Acetic acid = 42.6 µg g_cat_^-1^ h^-1^ |  |
| CN_x_/Ni_2_P  (1.6 mg ml^-1^) | AM 1.5G,  100 mW cm^-2^ (λ > 420 nm)  25 °C, N_2_, 4 h | Pretreated PP  (50 mg/mL in 2M KOH at 40 °C for 24 h) | H_2_ = 6.80  (µmol_H2_ g_cat_^-1^ h^-1^) | [3] |
| Co-Ga_2_O_3_  (50 mg) | 300 W Xenon Light,  100 mL DI Water, Oxygen, 24 h | PP  (100 mg) | H_2_ = 647.8 µmol g^-1^ h^-1^  Co = 158.3 µmol g^-1^ h^-1^ | [4] |
| VPOM/g-C_3_N_4_  (10 mg) | 300 W Xenon Light  (λ > 420 nm),  10 mL acetonitrile, O_2_, 36 h | PP  (20 mg) | Formic acid = 26.68  µmol g^-1^ h^-1^ | [5] |
| Ru/TiO_2_  (20 mg) | 300 W Xenon Light, Ar, 300 °C,  20 h | PP  (80 mg) | Selectivity to liquid/waxy products (C_5+_) = 95% & gaseous products (C_1_-C_4_) = 5% | [6] |
| RuMn/CeO_2_  (80 mg) | 10 bar H_2_, 250 °C, 8h | LDPE  (800 mg) | Yield CH_4_= 99.2 % | [7] |
| Pd_1_-TiO_2_  (10 mg) | 300 W Xenon Light | Pretreated PE (10 ml)  (Nitric acid 7% at 160 °C, 8 h) | C2 products (C_2_H_4_ & C_2_H_6_) = 1033 µmol g^-1^ h^-1^  Propanoic acid= 164.4 µmol h^-1^ | [8] |
| Zr-CoFe_2_O_4_ QDs  (20 mg) | 300 W Xenon Light, 20 ml DI water, 8 ml H_2_O_2_ | PE  (100 mg) | Acetic acid= 1.1 mmol g^-1^ h^-1^ | [9] |
| MoS_2_/CdS  (50 mg) | 300 W Xenon Light, 5h | Pretreated PE  (Nitric acid 6% at 180 °C, 5 h) | Methane= 196.2 µmol g^-1^ h^-1^  Ethane + Propane + n-pentane=  10.24 µmol g^-1^ h^-1^  H_2_ = 0.9 mmol g^-1^ h^-1^ | [10] |
| Floatable hybrid TiO_2_ | 300 W Xenon Light, mixing of 50 mg catalyst and 150 mg plastic | PE | <38 µmol g^-1^ h^-1^ (C_2_H_4_O, C_2_H_5_OH, CH_3_OH) | [11] |
|  |  | PP | < 55 µmol g^-1^ h^-1^ (Major: C_2_H_5_OH, C_2_H_4_O, CH_3_OH and Minor: CO, CH_4_, C_2_H_4_, C_3_H_6_, H_2_) |  |

**References**

[1] C. Kang, L. Jing, T. Guo, H. Cui, J. Zhou, H. Fu, Mesoporous SiO2-Modified Nanocrystalline TiO2 with High Anatase Thermal Stability and Large Surface Area as Efficient Photocatalyst. *J. Phys. Chem. C.* **2009**, *113*, 1006.

[2] X. Jiao, K. Zheng, Q. Chen, X. Li, Y. Li, W. Shao, J. Xu, J. Zhu, Y. Pan, Y. Sun, Y. Xie, Photocatalytic Conversion of Waste Plastics into C2 Fuels under Simulated Natural Environment Conditions. *Angew. Chem. Int. Ed.* **2020**, *59*, 15497–15501.

[3] T. Uekert, H. Kasap, E. Reisner, Photoreforming of Nonrecyclable Plastic Waste over a Carbon Nitride/Nickel Phosphide Catalyst. *J. Am. Chem. Soc.* **2019**, *141*, 15201−15210.

[4] J. Xu, X. Jiao, K. Zheng, W. Shao, S. Zhu, X. Li, J. Zhu, Y. Pan, Y. Sun, Y. Xie, Plastics-to-syngas photocatalysed by Co–Ga2O3 nanosheets. *National Sci. Rev.* **2022**, *9*, nwac011.

[5] C. Xing, G. Yu, J. Zhoua, Q. Liu, T. Chen, H. Liu, X. Li, Solar energy-driven upcycling of plastic waste on direct Z-scheme heterostructure of V-substituted phosphomolybdic acid/g-C3N4 nanosheets. *Appl. Catal. B. Environ.* **2022**, *315*, 121496.

[6] Y. Miao, Y. Zhao, G. I. N. Waterhouse, R. Shi, L. Wu, T. Zhang, Photothermal recycling of waste polyolefin plastics into liquid fuels with high selectivity under solvent-free conditions. *Nat. Commun.* **2023**, *14*, 4242.

[7] M. Zhao, X. Chu, F. Wang, Y. Fang, L. Sun, Q. Xie, L. Zhan, S. Song, H. Zhang, X. Wang. Enhancing the Conversion Efficiency of Polyethylene to Methane through Codoping of Mn Atoms into Ru Centers and CeO2 Supports. *J. Am. Chem. Soc.* **2024**, *146*, 33104-33111.

[8] S. Zhang, B. Xia, Y. Qu, L. Jing, M. Jaroniec, J. Ran, S-Z, Qiao. Photocatalytic production of ethylene and propionic acid from plastic waste by titania-supported atomically dispersed Pd species. *Sci. Adv.* **2023**, *9*, eadk2407.

[9] X. Jiao, Z. Hu, K. Zheng, J. Zhu, Y. Wu, X. Zhang, J, Hu, W. Yan, J. Zhu, Y. Sun, Y. Xie. Direct Polyethylene Photoreforming into Exclusive Liquid Fuel over Charge-Asymmetrical Dual Sites under Mild Conditions. *Nano. Lett.* **2022**, *22*, 10066-10072.

[10] M. Du, Y. Zhang, S. Kang, X. Guo, Y. Ma, M. Xing, Y. Zhu, Y. Chai, B. Qiu. Trash to Treasure: Photoreforming of Plastic Waste into Commodity Chemicals and Hydrogen over MoS2-Tipped CdS Nanorods. *ACS Catal.* **2022**, *12*, 12823-12832.

[11] M. Jiang, J. Li, X. Wan, J. Qiu, T. Yao, W. Zhang, S. Ma, H. Tan, A. Han, C. Chen, G. Liu. Floatable organic-inorganic hybrid-TiO2 unlocks superoxide radicals for plastic photoreforming in neutral solution. *Nat. Commun.* **2025**, *16*, 4136.
